# Analysis of durations and frequencies of mouthing behaviors in children 6 months to 6 years: an advanced videotaping and translation approach

**DOI:** 10.3389/fpubh.2026.1817680

**Published:** 2026-05-12

**Authors:** Foluke Adelabu, Cristina Fayad-Martinez, Olusola Olabisi Ogunseye, Jenna Honan, Maribeth Gidley, Paloma I. Beamer, Helena Solo-Gabriele, Alesia Ferguson

**Affiliations:** 1 Department of Built Environment, North Carolina Agricultural and Technical State University, Greensboro, NC, United States; 2 The Cooperative Institute For Marine And Atmospheric Studies, University of Miami, Miami, FL, United States; 3 Department of Community, Environmental and Policy, Mel and Enid Zuckerman College of Public Health, University of Arizona, Tucson, AZ, United States

**Keywords:** children, exposure assessment, hand-to-mouth contact, indoor environment, MLATs, mouthing behavior, object-to-mouth contact, video observation

## Abstract

**Background:**

Children are susceptible to harmful substances found in indoor dust through frequent hand-to-mouth and object-to-mouth contacts. Despite the importance of this exposure route, few studies have quantified the frequency, duration, or patterns of mouthing behaviors in indoor residential settings, creating uncertainty in exposure and risk models.

**Objective:**

This study quantified the frequency and duration of indoor mouthing contacts for children 6 months to 6 years across sociodemographic variables including age, sex, ethnicity, race and geographic region using advanced videotaping and video translation methodologies.

**Methods:**

Ninety-nine children were videotaped for 3–4 h during natural indoor play across three U.S. regions (Arizona, Florida, and North Carolina). 360-degree cameras were mounted in different rooms to capture natural behavior without researcher presence. Footage was tracked and merged sequentially, then translated into Micro-Level Activity Time Series (MLATS) using the Virtual Timing Device (VTD), documenting each mouthing contact with specific surfaces and household locations to generate frequency (contacts/h) and duration (minutes/h) data.

**Results:**

Mouthing duration showed significant age-related differences (Kruskal–Wallis *χ*^2^ = 12.0, *p* = 0.007), with median duration declining from 18.67 min/h in children 6–<12 months to 5.02 min/h in children 3–<6 years. Four surfaces accounted for the majority of mouthing duration: “other food” (4.25%), “pacifiers” (3.91%), “food containers” (3.41%), and “hands” (2.92%). Pacifier use showed complete discontinuation by age 3–<6 years (*p* = 0.008). No significant differences in total mouthing duration or frequency were observed across sex, race, ethnicity, or geographic region, suggesting that age-stratified exposure factors may be sufficient without additional demographic stratification.

**Conclusion:**

This study provides age-specific mouthing contact data to improve children’s exposure and risk estimates. The finding that duration decreases significantly with age while frequency remains stable suggests that exposure models should incorporate both parameters. The 360-degree camera methodology reduced observer interference while capturing comprehensive behavioral data across household microenvironments.

## 1 Introduction

Young children’s natural exploratory behaviors and developmental characteristics make them uniquely vulnerable to environmental contaminants. As they explore their surroundings, young children routinely put objects and their hands in their mouths, creating direct pathways for exposure to hazardous substances found in household environments (1–4). These mouthing behaviors can expose children to multiple classes of contaminants, including heavy metals (lead, arsenic, cadmium, mercury), flame retardants (polybrominated diphenyl ethers), per- and polyfluoroalkyl substances (PFAS), pesticides, and polycyclic aromatic hydrocarbons (5, 6).

Research has demonstrated links between environmental exposures, elevated biomarker contaminant levels, neurodevelopmental impairments, and behavioral disorders during critical developmental periods (7–9). Exposure risk is particularly pronounced in indoor environments, where household dust, a reservoir to contaminants, accumulates on everyday objects, for example, lead from deteriorating paint, PFAS from treated furniture and carpets, flame retardants from upholstered products and electronics, and pesticide residues from residential applications (3, 10, 11).

Characterizing and quantifying mouthing behaviors is essential for assessing dermal absorption and non-dietary ingestion of contaminants for indoor settings (12–14). While these behaviors create exposure risks, they also serve important developmental functions in early childhood. Infants derive comfort from sucking on pacifiers or fingers (3), while toddlers use mouthing as exploratory behavior to investigate their environment (4). This behavior can intensify during teething, providing relief from associated discomfort (13). Additionally, mouthing activities vary considerably among children, influenced by age, cultural practices, environmental setting, and individual temperament, complicating generalizations regarding exposure risks (12). This natural variability underscores the importance of collecting behavioral data across diverse populations and age groups to inform accurate risk assessments. A growing body of research has expanded beyond the earlier U.S.-focused studies reviewed by the EPA. Cross-cultural studies in Bangladesh (1, 15), Taiwan (12, 16), the Netherlands (17), and Korea (18) have documented mouthing frequencies across diverse populations. Recent advances in computer vision have introduced automated methods for quantifying microactivities (19, 20), offering alternatives to labor-intensive manual video coding.

Recognizing the critical need for standardized exposure data to inform risk assessments, the 1996 Food Quality Protection Act (FQPA) fundamentally changed how the U.S. Environmental Protection Agency (EPA) regulates pesticides, requiring consideration of children’s unique susceptibilities and aggregate exposures from multiple sources (21). In response to FQPA mandates, EPA identified dermal and non-dietary ingestion exposure assessments as high priorities, recognizing that these routes are difficult to measure due to complex human behavior and exposure mechanisms (22). This regulatory need drove the development of detailed micro-activity data collection methods and exposure models, including Micro-Level Activity Time Series (MLATS). MLATS were developed to preserve the sequence of contact events and locations visited by children, providing the foundation for exposure calculations used in pesticide registration and risk assessment (23, 24). MLATS data document each hand, mouth, or any body part contact with objects and assign exposure mechanisms to specific contact events, enabling more accurate estimation of aggregate and cumulative pesticide exposures through dietary, residential, and environmental pathways.

The need for accurate behavioral data extends beyond pesticide regulation to broader exposure assessment applications. EPA’s Exposure Factors Handbook provides recommended values for soil and dust ingestion, but as noted in the 2011 edition and subsequent updates, considerable uncertainty remains in these estimates, particularly for young age groups (25). Recognizing these data gaps, EPA recently issued a call for research to improve estimates of children’s soil and dust ingestion rates across different ages, geographical regions, and sociodemographic backgrounds (26). The agency has emphasized the need for expanded micro-level activity data to reduce uncertainty in exposure factor distributions and improve the scientific basis for updating the Exposure Factors Handbook (27). Current modeling efforts, including the Stochastic Human Exposure and Dose Simulation (SHEDS) framework, rely heavily on mouthing frequency and duration data to estimate non-dietary ingestion rates, yet available datasets remain limited in sample size, socio-demographic and geographic diversity (28, 29). This gap between regulatory needs and available data highlights the importance of comprehensive studies that capture behavioral patterns across diverse populations and settings.

Recent modeling efforts have refined these estimates. Özkaynak et al. used the SHEDS-Soil/Dust model to predict age-specific soil and dust ingestion rates across ten age ranges from birth to 21 years, estimating overall mean rates ranging from approximately 35 mg/day for infants to approximately 60 mg/day for toddlers and young children (30). Cohen et al. conducted a meta-analysis of available soil and dust ingestion studies for children and adults in the United States and Canada, advancing beyond the simple averaging of estimates used in EPA’s Exposure Factors Handbook (31). Li et al. introduced a mechanistic model for mouthing-mediated chemical ingestion that highlighted the dependence of exposure estimates on mouthing parameters and indoor dust dynamics (32).

Previous mouthing behavior studies reviewed by the EPA Exposure Factors Handbook have notable limitations in sample size, observation duration, and population diversity. While these studies provided foundational estimates, EPA has acknowledged that confidence in its current recommended microactivity frequency estimates remains low (19). Video-based studies, for example, ranged from 4 to 52 children. Zartarian et al. studied only 4 children (33), and EPA noted the “sample of children studied was very small and not likely to be representative of the national population.” Reed et al. included (34) “only a small number of children” (*n* = 30), with some “not selected randomly.” Freeman et al. videotaped only 19 of 168 surveyed children (35). Black et al. studied 52 children (36), but EPA noted the “sample was fairly small and was from a limited area.” Observation durations were also limited, with Tulve et al. using only 15-min observation periods (37). For parent-observation studies, EPA noted additional methodological concerns, Juberg et al. studied 385 children (38), but “the children apparently were not selected randomly” and “there is no description of the socioeconomic status or racial and ethnic identities of the study participants.” More recent work has expanded sample sizes; Lopez-Galvez et al. analyzed micro-level activity data for 80 children aged 1–12 years in outdoor turf and playground settings, representing the largest video-based mouthing study prior to the current work (4). However, that study focused on a single farmworker community in California, and like earlier studies, did not report room-level location data.

In direct response to EPA’s call for expanded micro-activity datasets, this study employed enhanced methodologies to capture and analyze mouthing behaviors among children aged 6 months to 6 years, creating a comprehensive data repository stratified by demographic categories. Specifically, the current study employed mounted 360-degree video cameras across multiple rooms in a home to capture young children’s natural behaviors in their home environments across three geographically and environmentally distinct U.S. regions and other demographic variables. Videotaping methods have been previously employed to observe and document children’s mouthing behavior (4, 34, 39). By integrating this less-intrusive methodology with systematic video translation techniques, as used by Ferguson et al. (39), we aimed to more accurately quantify mouthing frequency and duration across different age groups and other demographic variables. This research contributes to understanding of exposure routes linked to indoor environments and supports refined risk assessments for soil/dust ingestion modeling.

## 2 Methods

### 2.1 Study design and population

This study collected and analyzed ingestion related behaviors as part of the DIRT (Dust Ingestion childRen sTudy) project. The study involved survey collection and fieldwork. As part of the larger study, 450 families with at least one child between the ages of 6 months to 6 years completed a demographic survey. From these, 101 children participated in fieldwork (i.e., during research team home visits) that entailed videotaping, to capture indoor play behavior, and soil/dust collection around the house. In multi-child households where multiple eligible children resided, priority was given to the youngest child for videotaping. Following data quality review, two children were excluded due to insufficient quality of videotapes, resulting in a final study population of 99 participants (i.e., children).

Recruitment was conducted across three geographic regions through established community partnerships including the Debbie Institute Mailman Center for Child Development in Miami, Florida; the Child Development Center at North Carolina Agricultural and Technical State University in Greensboro, North Carolina; and El Rio Community Health Center and the Sonoran Environmental Research Institute in Tucson, Arizona. Additional recruitment occurred at daycare centers, libraries, health department clinics, and through social media platforms to ensure demographic diversity. The recruitment procedures and overall study design of DIRT are described in detail by Ferguson et al. (27).

Prior to participant recruitment, approval was obtained from the Institutional Review Board at North Carolina Agricultural and Technical State University (IRB #21-0032), under which the University of Arizona and University of Miami operated through reliance agreements. Written informed consent was obtained from all participating parents or guardians prior to data collection.

### 2.2 Data collection and videotaping

Following informed consent procedures, the comprehensive DIRT fieldwork protocol required approximately 5 h per household visit, encompassing parental surveys, dust collection, hand rinsing procedures, and videotaping (3–4 h of total visit duration). The current study focuses only on videotaping data.

To optimize viewing angles and minimize researcher interference, the study mounted up to six Insta360 One X2 cameras across multiple rooms in each household. The number of cameras varied depending on size of the house, and access to power outlets. Each camera was uniquely labeled (1 through 6) for identification and connected directly to power outlets to enable extended recording periods without battery limitations. Cameras were strategically positioned throughout participant homes, with placement prioritizing rooms where children spent the most time and positioning designed to capture sequential footage as children moved between spaces. Camera mounting employed multiple methods including command strips, furniture clamps on bookshelves and tabletops, and tripod stands positioned behind furniture to prevent accidental disconnection, and limit damage to surfaces.

All recording sessions took place during daytime and evening waking hours; no overnight recordings were conducted. Following camera setup, research teams departed the premises to allow families to maintain normal routines without researcher presence or interference. Teams remained accessible by phone for technical assistance and returned after 3–4 h to stop recording, retrieve equipment and complete all other field tasks. Video footage was immediately saved to SD cards and transferred to external drives upon return to laboratory facilities, with all footage backed up immediately following collection. Original videos were saved to 12-terabyte external hard drives, with two drives for 33 children (at each location) and two additional backup drives (12 external hard drives in total before processing). Drives from Florida and Arizona teams were forwarded to North Carolina for centralized processing and analysis. Participants received $100 compensation upon completion of all field study procedures.

### 2.3 Video processing

The video processing phase consisted of three distinct stages: video tracking, video merging, and segmentation for translation. The initial processing stage utilized Insta360 Studio software to deep track individual participants as they moved throughout their home environments. The tracking process involved selecting each child as the subject for deep tracking, removing portions of footage where the child was not visible and exporting processed footage in H.264 format with appropriate encoding settings (i.e., Artificial Intelligence effects “Remove Grain” enabled, bitrate set to 75). This process was repeated systematically until all camera feeds had been processed.

Following individual camera tracking, footage was merged sequentially using Adobe Premiere Pro to create activity recordings for each participant. Tracked footage from each camera was imported and arranged in chronological order based on actual child movement patterns observed during recording. When multiple cameras captured the same room from different angles, the camera providing optimal viewing conditions for mouth and hand activities was selected for the merged sequence. The complete video processing workflow generated 277.35 h of raw footage across all 99 participants.

### 2.4 Video translation

The translation of segmented video footage into MLATS was conducted using the Virtual Timing Device (VTD), a computerized platform designed for detailed analysis of children’s activity patterns from video observations (24, 39). The VTD employs a customized palette featuring three integrated grids: possible activity levels (repetitive or constant), household locations, and objects/surfaces categories. Once a sub-grid is activated in each grid, a clock registers the seconds of combined selections. These categories were based on previous studies (24, 29, 40, 41) and further modified for this study (See Supplementary Figure S1).

The translation process requires sustained attention to detail as translators must identify brief mouthing contacts that may last only seconds while simultaneously tracking the child’s location within the household. Translators were trained research assistants who viewed recorded footage and systematically coded each observed behavior using the VTD platform. Translator training utilized three 15 min video segments representing varying activity intensities: low, moderate, and high child activity levels. Training commenced with memorization of the study palette (Supplementary Figure S1) until translators demonstrated mastery in achieving 90% inter-observer agreement with the lead trainer’s reference translations for surface type, location, and activity level.

Additionally, surfaces and objects were classified into high, medium, and low soil and dust accumulation categories based on typical characteristics that would lead to accumulation of soil or dust on surfaces. Food-related surfaces were subdivided into “other food” (all food types except sticky foods, e.g., crackers, bread, vegetables), “food container” (utensils, feeding bottles, candy bag, sippy cups) and “sticky food” (candy, lollipops, fruits). The complete surface and object categorization table is provided in Supplementary Table S1. When two objects were mouthed simultaneously, translators selected the surface most likely to have the highest loading of soil or dust based on this conservative ranking system (i.e., considered the object/surface with the greater soil or dust accumulation). When ambiguity existed about the nature of the object being contacted, the conservative choice was also made. Household locations were coded across 11 categories including living room, bedroom, kitchen, dining room, bathroom, playroom, corridors, balcony/porch, den, garage, and laundry room (Supplementary Figure S1). Lastly, activity levels were coded as either constant or repetitive to describe the rate at which children mouthed items. Constant activity referred to continuous, non-stop mouthing of an object, while repetitive activity referred to intermittent mouthing patterns with brief pauses between contacts, similar to repeatedly bringing an object to the mouth and then removing it.

Systematic quality control measures were implemented throughout the translation process. Five trained translators contributed to the final coded dataset. Merged footage was divided into 586 segments of approximately 30 min each prior to video translation to minimize potential translator fatigue and maintain accuracy during the detailed coding process. Following every ten completed translations, the lead translator, or other translators independently, conducted a spot check by re-translating a randomly selected segment and comparing the resulting codes against the original translator’s output. This independent re-coding procedure was applied to 58 segments (10% of the total dataset). Agreement was assessed using percent agreement across all coded dimensions (surface type, location, and contact timing). Of the spot-checked segments, 30% (*n* = 18) required retranslation due to discrepancies. When disagreements were identified, the lead translator and original translator reviewed the segment together. Discrepancies were resolved through discussion, and the segment underwent complete re-translation to achieve 100% agreement before the translator continued. If multiple segments from the same translator demonstrated agreement issues, additional retraining was implemented before translation continuation. When the child’s mouth was not fully visible to the camera but the ongoing mouthing activity could be reliably inferred from observable contextual cues (e.g., visible chewing motion, an object held to the mouth that remained identifiable, or continuation of an ongoing eating or pacifier use episode), the contact was coded according to the inferred surface and activity. Contacts were coded as “Not-In-View” only when neither the mouth nor sufficient contextual cues were visible to determine the activity with confidence. The translation process generated detailed MLATS data (i.e., text files) for subsequent statistical analysis.

### 2.5 Data processing

Video translation data were processed separately for surface/object mouthing analyses and location analyses. Two distinct “Not-In-View” categories were identified: (1) Surface “Not-In-View” occurred when the child’s mouth was not visible on camera (e.g., child facing away, mouth obscured), and (2) Location “Not-In-View” occurred when the child moved to a location without camera coverage. A detailed summary of data quality metrics, including total recorded versus usable footage, proportions of Surface and Location Not-In-View time, and technical data loss, is provided in Supplementary Table S2.

For surface/object mouthing analyses, Surface “Not-In-View” periods were excluded from calculations. The “Nothing” surface category represented time when the mouth was visible, but no mouthing contact occurred. Hourly rates for mouthing duration (minutes per hour) and frequency (contacts per hour) were calculated by dividing total time spent mouthing specific surfaces (or total number of contacts) by total observation time (excluding Surface “Not-In-View”). This denominator includes “Nothing” time to provide accurate exposure rate estimates. For location analyses, Location “Not-In-View” periods were excluded from calculations. Time spent in each location and location frequency were calculated using total observation time (excluding Location “Not-In-View”).

For analyses examining the overlap of surface mouthing within specific locations, only periods when both the location had camera coverage and the child’s mouth was visible were included (i.e., excluding both Surface and Location “Not-In-View”). “Nothing” was excluded to focus on active mouthing behaviors in specific rooms.

### 2.6 Statistical analysis

All statistical analyses were conducted using R software (version 4.5.2). Descriptive statistics including means, standard deviations, medians, interquartile ranges, and ranges for mouthing contact frequencies and durations were calculated across demographic groups, surface types, and locations. Data distributions were assessed using the Shapiro–Wilk normality test, which indicated that mouthing data were not normally distributed across all demographic categories, surface types, and location types (all *p* < 0.05). Consequently, non-parametric statistical tests were employed for all behavioral comparisons.

For demographic variables with two categories (ethnicity: Hispanic versus non-Hispanic), the Mann–Whitney U test was used to compare mouthing behaviors between groups. For demographic variables with more than two categories (age groups: 6 to <12 months, 1 to <2 years, 2 to <3 years, and 3 to <6 years; sex: Female, Male, Other; race: Asian, Black or African American, Mixed, Other, White; study regions: Arizona, Florida, North Carolina), the Kruskal–Wallis test was applied to assess differences in mouthing behaviors across groups. Following significant Kruskal–Wallis tests (*p* < 0.05), *post hoc* pairwise comparisons were conducted using Wilcoxon rank-sum tests with Bonferroni correction to control for multiple comparisons and maintain the family-wise error rate at *α* = 0.05. Chi-square tests of independence were used to examine the association between surface type and household location. All statistical tests used α = 0.05 as the significance threshold. Sample sizes for each comparison are reported with test statistics in figure captions and supplementary tables. This study involved a large number of statistical comparisons across demographic variables, surface types, and household locations, which increases the chance of false positive results. To address this, Bonferroni correction was applied to all pairwise comparisons that followed a significant Kruskal–Wallis test (e.g., comparing each pair of age groups after finding an overall age effect). However, no correction was applied for the total number of separate Kruskal–Wallis or Mann–Whitney tests conducted across different demographic variables and locations, as each addressed a distinct research question. As a result, some significant findings at the location level (e.g., dining room differences by region) may reflect chance rather than true effects. Additionally, the absence of significant differences for some demographic variables should be interpreted with caution, as small subgroup sizes (e.g., Asian *n* = 6, Other race *n* = 3) may have limited the statistical power to detect real differences.

## 3 Results

The sample of 99 children was distributed across four EPA age groups: 6 to <12 months (*n* = 17, 17.2%), 1 to <2 years (*n* = 25, 25.3%), 2 to <3 years (*n* = 24, 24.2%), and 3 to <6 years (*n* = 33, 33.3%) (EPA 2005), evenly recruited among Arizona (*n* = 33), Florida (*n* = 33), and North Carolina (*n* = 33). The sample included 47 females (47.5%), 51 males (51.5%), and 1 participant who identified as other sex (1.0%). Racial distribution consisted of White (*n* = 50, 50.5%), Black or African American (*n* = 25, 25.3%), Mixed race (*n* = 15, 15.2%), Asian (*n* = 6, 6.1%), and Other (*n* = 3, 3.0%), with 39 participants (39.4%) identifying as Hispanic and 60 (60.6%) as non-Hispanic (Table 1).

**Table 1:** Demographic characteristics of study participants (*n* = 99).

Variable	Category	*n*	Obs. with NIV (hours)	Obs. without NIV (hours)	Nothing (%)	Active (%)	Mouthing duration (min/h)	Mouthing frequency (contacts/h)
Age group	12 to <24 months	25	2.67 ± 0.78	2.39 ± 0.79	80.7 ± 17.4	19.3 ± 17.4	11.59 ± 10.41	35.76 ± 25.44
	24 to <36 months	24	2.82 ± 0.76	2.57 ± 0.75	80.2 ± 21.5	19.8 ± 21.5	11.89 ± 12.9	26.7 ± 19.36
	36 to <72 months	33	2.7 ± 0.83	2.57 ± 0.84	85.6 ± 15.3	14.4 ± 15.3	8.66 ± 9.19	26.95 ± 22.61
	6 to <12 months	17	3.16 ± 0.76	2.87 ± 0.82	71.9 ± 14.3	28.1 ± 14.3	16.83 ± 8.55	28.62 ± 25.02
Region	Arizona	33	2.91 ± 0.85	2.74 ± 0.84	84.4 ± 12.7	15.6 ± 12.7	9.37 ± 7.63	24.67 ± 15.61
	Florida	33	2.81 ± 0.83	2.54 ± 0.84	78.9 ± 20.5	21.1 ± 20.5	12.67 ± 12.28	30.28 ± 24.43
	North Carolina	33	2.69 ± 0.72	2.45 ± 0.73	78.8 ± 18.9	21.2 ± 18.9	12.72 ± 11.35	33.25 ± 27.26
Sex	F	47	2.73 ± 0.85	2.52 ± 0.87	80.5 ± 17	19.5 ± 17	11.7 ± 10.2	25.46 ± 18.05
	M	51	2.9 ± 0.71	2.66 ± 0.71	80.8 ± 18.7	19.2 ± 18.7	11.5 ± 11.19	33.26 ± 26.57
	O	1	0.94 ± NA	0.85 ± NA	81.6 ± NA	18.4 ± NA	11.05 ± NA	17.67 ± NA
Race	Asian	6	3.12 ± 0.24	2.87 ± 0.58	87.7 ± 7.7	12.3 ± 7.7	7.39 ± 4.61	38.13 ± 18.01
	Black or African American	25	2.75 ± 0.78	2.54 ± 0.73	81.2 ± 16.6	18.8 ± 16.6	11.31 ± 9.95	32.62 ± 27.15
	Mixed	15	2.62 ± 0.95	2.45 ± 1	81.5 ± 15.9	18.5 ± 15.9	11.12 ± 9.55	22.61 ± 15.39
	Other	3	2.82 ± 0.65	2.64 ± 0.72	90.7 ± 4.7	9.3 ± 4.7	5.56 ± 2.8	31.08 ± 29.3
	White	50	2.85 ± 0.81	2.59 ± 0.82	78.8 ± 19.9	21.2 ± 19.9	12.73 ± 11.95	28.67 ± 23.09
Ethnicity	Hispanic	39	2.84 ± 0.87	2.61 ± 0.91	78.5 ± 20.6	21.5 ± 20.6	12.88 ± 12.39	28.2 ± 25.06
	Non-Hispanic	60	2.78 ± 0.75	2.55 ± 0.73	82.1 ± 15.5	17.9 ± 15.5	10.75 ± 9.31	30.18 ± 21.77
Overall		99	2.8 ± 0.8	2.58 ± 0.8	80.7 ± 17.7	19.3 ± 17.7	11.59 ± 10.62	29.4 ± 23.02

NIV, not in view; Obs., observation; SD, standard deviation. One child identifying as other sex was included in overall analyses but excluded from sex-stratified comparisons.

Mean total videotaped time ranged from 2.67 to 3.16 h across age groups, which reduced to 2.39–2.87 h after excluding periods when the child’s mouth was not visible on camera (Surface “Not-In-View”). Children spent the majority of their observation time with no active mouthing (71.9–85.6%), with active mean mouthing time decreasing with age from 28.1% in younger children aged 6 to <12 months to 14.4% in older children aged 3 to <6 years. Surface Not-In-View accounted for a median of 2.4% (IQR: 0.7–6.6%) of each child’s translated footage, while Location Not-In-View accounted for a median of 0.15% (IQR: 0.0–1.5%) (Supplementary Table S2).

Mouthing frequency, however, showed no significant differences across any demographic variable: age (Kruskal–Wallis *χ*^2^ = 2.382, df = 3, *p* = 0.497), region (Kruskal–Wallis *χ*^2^ = 0.632, df = 2, *p* = 0.729), sex (Kruskal–Wallis *χ*^2^ = 1.777, df = 2, *p* = 0.411), race (Kruskal–Wallis *χ*^2^ = 2.984, df = 4, *p* = 0.560), or ethnicity (Mann–Whitney U = 1,058, *p* = 0.425), with an overall mean of 29.37 ± 22.95 contacts/h and median values relatively consistent across age groups (21.26 to 27.79 contacts/h). Detailed distributions by all demographic variables are presented in Table 1, and a comprehensive comparison with previous mouthing behavior studies is provided in Supplementary Table S3.

### 3.1 Object/surface mouthing contact duration

Four objects/surfaces accounted for the majority of mouthing duration. These included “other food” (651 min, 4.25% of in-view observation time), “pacifiers” (598 min, 3.91%), “food containers” (522 min, 3.41%), and “hands” (447 min, 2.92%), together representing 2,218 of 2,867 total active mouthing minutes across all subjects. The second tier included “hair and body” (252 min, 1.64%), “sticky food” such as candy, fruits, and lollipops (125 min, 0.81%), and “porous plastic toy” such as crayons, balloons, and plastic pencils (82 min, 0.54%), while remaining surface types collectively accounted for 1.25% of in-view observation time (Table 2).

**Table 2:** Object/surface observation time breakdown.

Surface	n subjects	Total minutes	Total hours	Mean Min/Hour	SD Min/Hour	Median Min/Hour	% of total
Animal	3	0.15	0.00	0.00	0.00	0.00	0.00
Bedding/Towels	21	65.32	1.09	0.32	1.68	0.00	0.43
Beverage	25	40.23	0.67	0.17	0.52	0.00	0.26
Carpet/Mat	1	0.20	0.00	0.00	0.01	0.00	0.00
Clothes	29	32.00	0.53	0.14	0.94	0.00	0.21
Electronics	7	2.85	0.05	0.01	0.07	0.00	0.02
Fabric_Toy	14	7.98	0.13	0.03	0.11	0.00	0.05
Fabric_Wall/Furn	20	5.40	0.09	0.02	0.07	0.00	0.04
Food-Cont	81	521.62	8.69	2.05	3.6	0.78	3.41
Footwear	2	0.08	0.00	0.00	0.00	0.00	0.00
Hair/Body	34	251.67	4.19	0.96	3.6	0.00	1.64
Hands	89	446.97	7.45	1.75	3.07	0.66	2.92
Hard_Toy	10	4.97	0.08	0.02	0.07	0.00	0.03
Metal_Tool/Appl	1	0.00	0.00	0.00	0.00	0.00	0.00
Metal_Wall/Furn	3	0.63	0.01	0.00	0.01	0.00	0.00
Nose	2	0.00	0.00	0.00	0.00	0.00	0.00
Nothing	99	12432.82	207.21	48.41	10.62	51.26	81.26
Other_Food	80	651.12	10.85	2.68	3.07	1.67	4.26
Pacifier	12	597.63	9.96	2.42	8.40	0.00	3.91
Paper/Wrapper	25	21.92	0.37	0.08	0.40	0.00	0.14
Plastic-Tool/Appl	10	6.78	0.11	0.03	0.19	0.00	0.04
Plastic_Wall/Furn	2	0.33	0.01	0.00	0.01	0.00	0.00
Porous-Plastic-Toy	41	82.30	1.37	0.30	1.13	0.00	0.54
Sticky_Food	27	124.62	2.08	0.59	2.07	0.00	0.81
Water	5	0.20	0.00	0.00	0.01	0.00	0.00
Wood_Toy	1	0.17	0.00	0.00	0.01	0.00	0.00
Wood_Wall/Furn	1	1.87	0.03	0.01	0.07	0.00	0.01

All observations correspond to 99 subjects. Total observation time = 255.00 h (Surface Not-In-View excluded). Percentages represent proportion of total surface observation time.

The category “other food” demonstrated the highest median mouthing duration (1.67 min/h, IQR: 0.32–3.84), while pacifier mouthing showed substantial variability with a median of 0.00 min/h but a 95th percentile of 17.68 min/h, reflecting that most children did not use pacifiers while some showed extensive use. Mouthing duration differed significantly across objects/surfaces (Kruskal–Wallis, *p* < 0.001), with *post-hoc* comparisons revealing that “other food,” “food containers,” and “hands” did not differ from each other (all adjusted *p* = 1.0) but each differed significantly from other surface types (all adjusted *p* < 0.001) (Table 3).

**Table 3:** Indoor hourly mouthing duration by object/surface (*n* = 99 subjects).

Objects/Surfaces	Mean	Min	5th	25th	50th	75th	95th	Max	SD
Animal	0.00	0.00	0.00	0.00	0.00	0.00	0.00	0.05	0.00
Bedding/Towels	0.32	0.00	0.00	0.00	0.00	0.00	0.61	11.96	1.68
Beverage	0.17	0.00	0.00	0.00	0.00	0.00	1.15	3.30	0.52
Carpet/Mat	0.00	0.00	0.00	0.00	0.00	0.00	0.00	0.14	0.01
Clothes	0.14	0.00	0.00	0.00	0.00	0.01	0.18	9.04	0.94
Electronics	0.01	0.00	0.00	0.00	0.00	0.00	0.01	0.61	0.07
Fabric_Toy	0.03	0.00	0.00	0.00	0.00	0.00	0.20	0.63	0.11
Fabric_Wall/Furn	0.02	0.00	0.00	0.00	0.00	0.00	0.10	0.52	0.07
Food-Cont	2.05	0.00	0.00	0.09	0.78	2.03	11.38	17.97	3.60
Footwear	0.00	0.00	0.00	0.00	0.00	0.00	0.00	0.02	0.00
Hair/Body	0.96	0.00	0.00	0.00	0.00	0.01	4.48	24.27	3.60
Hands	1.75	0.00	0.00	0.11	0.66	2.03	7.50	17.50	3.07
Hard_Toy	0.02	0.00	0.00	0.00	0.00	0.00	0.10	0.51	0.07
Metal_Tool/Appl	0.00	0.00	0.00	0.00	0.00	0.00	0.00	0.00	0.00
Metal_Wall/Furn	0.00	0.00	0.00	0.00	0.00	0.00	0.00	0.14	0.01
Nose	0.00	0.00	0.00	0.00	0.00	0.00	0.00	0.00	0.00
Nothing	48.41	8.42	25.2	45.34	51.26	55.56	58.57	59.95	10.62
Other_Food	2.68	0.00	0.00	0.32	1.67	3.84	8.82	15.62	3.07
Pacifier	2.42	0.00	0.00	0.00	0.00	0.00	17.68	48.09	8.4
Paper/Wrapper	0.08	0.00	0.00	0.00	0.00	0.00	0.27	3.52	0.4
Plastic_Wall/Furn	0.00	0.00	0.00	0.00	0.00	0.00	0.00	0.09	0.01
Plastic-Tool/Appl	0.03	0.00	0.00	0.00	0.00	0.00	0.09	1.67	0.19
Porous-Plastic-Toy	0.30	0.00	0.00	0.00	0.00	0.10	1.20	9.92	1.13
Sticky_Food	0.59	0.00	0.00	0.00	0.00	0.01	2.33	12.72	2.07
Water	0.00	0.00	0.00	0.00	0.00	0.00	0.00	0.11	0.01
Wood_Toy	0.00	0.00	0.00	0.00	0.00	0.00	0.00	0.06	0.01
Wood_Wall/Furn	0.01	0.00	0.00	0.00	0.00	0.00	0.00	0.65	0.07

Values in min/h. Observation time = 255.00 h (Surface Not-In-View excluded).

### 3.2 Object/surface mouthing duration by demographics

**Figure 1:**
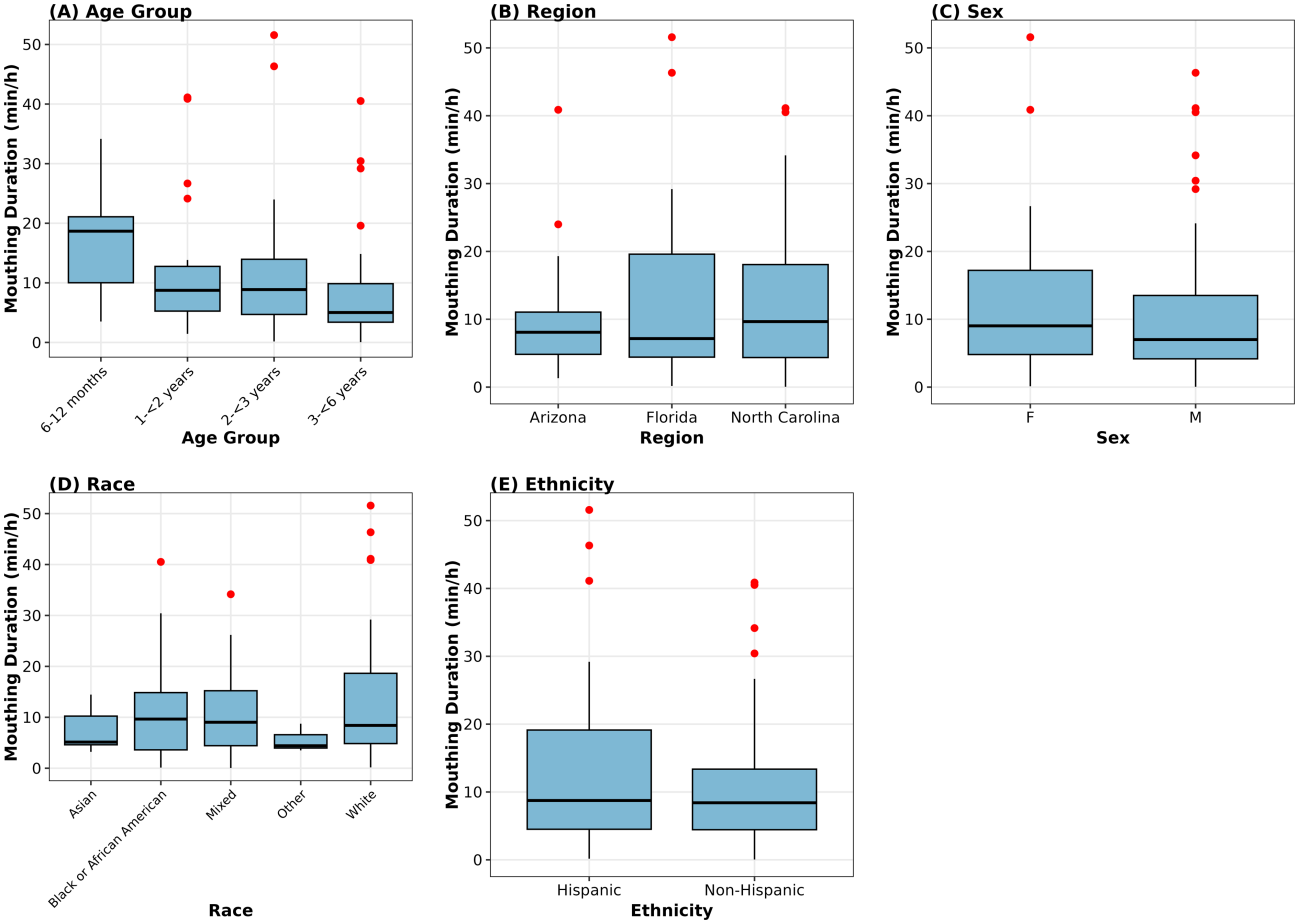
Total active mouthing duration (min/h) by demographic variables (*n* = 99). Box plots show median (horizontal line), interquartile range (box), 1.5 times IQR (whiskers), and outliers (dots) across **(A)** age categories showing significant differences (Kruskal Wallis *χ*^²^ = 12.028, df = 3, *p* = 0.007), **(B)** geographic regions (*χ*^²^ = 0.727, df = 2, *p* = 0.695), **(C)** sex (*χ*^²^ = 0.541, df = 2, *p* = 0.763), **(D)** race (*χ*^²^ = 2.169, df = 4, *p* = 0.705), and **(E)** ethnicity (Mann Whitney U = 1247, *p* = 0.584). Sample sizes: Age groups (*n* = 17, 25, 24, 33); Regions (Arizona = 33, Florida = 33, North Carolina = 33); Sex (F = 47, M = 51, O =1); Race (Asian = 6, Black or African American = 25, Mixed = 15, Other = 3, White = 50); Ethnicity (Hispanic = 39, Non-Hispanic = 60). One participant reporting ‘Other’ sex (*n* = 1) was included in statistical testing but excluded from the sex panel for visualization due to insufficient sample size.

Mouthing duration showed significant age-related differences (Kruskal–Wallis *χ*^2^ = 12.028, df = 3, *p* = 0.007). Median mouthing duration declined from 18.67 min/h in children 6 to <12 months to 8.75 min/h in children 1 to <2 years, 8.87 min/h in children 2 to <3 years, and 5.02 min/h in children 3 to <6 years (Figure 1A). *Post-hoc* pairwise comparisons with Bonferroni correction revealed that only the comparison between children 6 to <12 months and 3 to <6 years reached significance (*p* = 0.004); no other pairwise comparisons were statistically significant. In contrast, no significant differences in total mouthing duration were observed across sex (*χ*^2^ = 0.541, df = 2, *p* = 0.763; Figure 1C), race (*χ*^2^ = 2.169, df = 4, *p* = 0.705; Figure 1D), ethnicity (Mann–Whitney U = 1,247, *p* = 0.584; Figure 1E), or geographic region (*χ*^2^ = 0.727, df = 2, *p* = 0.695; Figure 1B).

#### 3.2.1 Surface/object mouthing duration by age

Given the significant age-related differences in total mouthing duration, we examined patterns for surfaces that accounted for the majority of active mouthing time (Table 4). All durations are presented over the in-view time of the child’s mouth.

**Table 4:** Surface mouthing duration (min/h) by age group.

Surface	6 to <12 months	1 to <2 years	2 to <3 years	3 to <6 years	*p*-value
Other_Food	0.32 (0.54 ± 0.66)	2.96 (2.65 ± 1.91)	2.08 (2.48 ± 2.36)	2.48 (3.94 ± 4.23)	0.001
Hands	1.66 (2.4 ± 4.08)	0.70 (1.27 ± 1.78)	0.72 (1.71 ± 2.13)	0.27 (1.82 ± 3.82)	0.492
Food-Cont	2.47 (5.23 ± 5.91)	0.93 (1.85 ± 3.33)	0.80 (1.99 ± 2.77)	0.22 (0.59 ± 0.96)	<0.001
Pacifier	0.00 (4.29 ± 7.47)	0.00 (2.73 ± 8.38)	0.00 (4.10 ± 13.21)	0.00 (0.00 ± 0.00)	0.007
Hair/Body	0.00 (2.86 ± 6.83)	0.00 (1.68 ± 4.05)	0.00 (0.15 ± 0.64)	0.00 (0.03 ± 0.13)	0.063
Porous-Plastic-Toy	0.03 (0.82 ± 2.38)	0.01 (0.19 ± 0.37)	0.00 (0.45 ± 1.02)	0.00 (0.01 ± 0.04)	0.002
Fabric_Toy	0.00 (0.09 ± 0.21)	0.00 (0.01 ± 0.04)	0.00 (0.03 ± 0.11)	0.00 (0.00 ± 0.00)	0.014
Sticky_Food	0.00 (0.08 ± 0.28)	0.00 (0.71 ± 2.07)	0.00 (0.07 ± 0.19)	0.00 (1.15 ± 3.02)	0.071
Beverage	0.00 (0.01 ± 0.03)	0.00 (0.06 ± 0.23)	0.00 (0.34 ± 0.77)	0.00 (0.22 ± 0.55)	0.170
Total Active Mouthing	18.67 (16.83 ± 8.55)	8.75 (11.59 ± 10.41)	8.87 (11.89 ± 12.9)	5.02 (8.66 ± 9.19)	0.007

Values shown as median (mean ± SD). Kruskal–Wallis test across four age groups. Observation time = 255.00 h (Surface Not-In-View excluded).

Food container duration showed the most pronounced age-related decline (Kruskal–Wallis *χ*^2^ = 22.163, df = 3, *p* < 0.001), with median duration decreasing from 2.47 min/h in children 6 to <12 months to 0.22 min/h in children 3 to <6 years (*post-hoc p* < 0.001). In contrast, other food duration showed a significant age-related increase (*χ*^2^ = 15.853, df = 3, *p* = 0.001), with median duration rising from 0.32 min/h in children 6 to <12 months to 2.48 min/h in children 3 to <6 years (*post-hoc p* = 0.002), reflecting a transition from container-based to independent feeding with age.

Pacifier duration showed significant age-related decline (*χ*^2^ = 11.933, df = 3, *p* = 0.008), with complete discontinuation by age 3 to <6 years (*post-hoc p* = 0.002 vs. 6 to <12 months). Similarly, porous plastic toy duration declined significantly (*χ*^2^ = 14.671, df = 3, *p* = 0.002), with children 3 to <6 years showing significantly lower duration compared to both children 6 to <12 months (*p* = 0.006) and children 1 to <2 years (*p* = 0.005). Fabric toy duration also declined with age (*χ*^2^ = 10.628, df = 3, *p* = 0.014), with significantly lower duration in children 3 to <6 years compared to children 6 to <12 months (*p* = 0.008) and children 1 to <2 years (*p* = 0.021).

#### 3.2.2 Object/surface mouthing duration by sex, race, ethnicity, and region

Object/surface analyses revealed limited demographic differences beyond age. Beverage mouthing duration showed a trend toward regional differences but did not reach statistical significance in *post-hoc* comparisons between North Carolina and Arizona (*p* = 0.091). No significant pairwise differences were found for race or sex after Bonferroni correction. Complete distributions are provided in Supplementary Tables S4–S10.

### 3.3 Object/surface mouthing contact frequency by demographics

The three primary objects/surfaces (“other food,” “food containers,” and “hands”) dominated contact frequency, representing 5,235 out of 7,232 mouthing contacts. The “other food” category (all other types of food except sticky foods) showed the highest median mouthing frequency (5.23 contacts/h), followed by “hands” (4.78 contacts/h), and “food containers” (3.82 contacts/h). These three objects/surfaces accounted for the majority of mouthing contact events across all subjects (Table 5; see Supplementary Table S4 for full details).

**Table 5:** Surface mouthing frequency (contacts/h) by age group.

Surface	6 to <12 months	1 to <2 years	2 to <3 years	3 to <6 years	*p*-value
Other_Food	3.37 (5.5 ± 7.65)	7.42 (9.41 ± 7.65)	5.50 (7.26 ± 8.14)	4.65 (7.32 ± 8.23)	0.194
Hands	5.24 (5.84 ± 4.6)	6.78 (9.55 ± 9.52)	5.49 (6.51 ± 6.22)	3.78 (6.7 ± 9.13)	0.686
Food-Cont	5.24 (8.75 ± 8.8)	5.27 (6.49 ± 6.23)	3.97 (5.78 ± 6.78)	2.03 (4.79 ± 7.22)	0.142
Pacifier	0.00 (1.10 ± 2.25)	0.00 (2.67 ± 8.26)	0.00 (0.84 ± 2.94)	0.00 (0.00 ± 0.00)	0.009
Hair/Body	0.00 (0.64 ± 1.08)	0.00 (1.33 ± 2.20)	0.00 (0.44 ± 1.25)	0.00 (0.22 ± 0.44)	0.123
Porous-Plastic-Toy	0.37 (3.50 ± 8.47)	0.34 (1.54 ± 3.34)	0.00 (1.26 ± 2.26)	0.00 (0.08 ± 0.18)	0.001
Fabric_Toy	0.00 (0.29 ± 0.60)	0.00 (0.18 ± 0.35)	0.00 (0.20 ± 0.54)	0.00 (0.00 ± 0.00)	0.018
Sticky_Food	0.00 (0.64 ± 2.21)	0.00 (1.18 ± 2.80)	0.00 (0.17 ± 0.48)	0.00 (3.69 ± 9.14)	0.048
Beverage	0.00 (0.07 ± 0.24)	0.00 (0.61 ± 2.36)	0.00 (2.54 ± 5.97)	0.00 (0.99 ± 1.90)	0.161
Plastic-Tool/Appl	0.00 (0.21 ± 0.48)	0.00 (0.46 ± 1.42)	0.00 (0.03 ± 0.09)	0.00 (0.00 ± 0.00)	0.050
Total Active Mouthing	23.46 (28.62 ± 25.02)	27.79 (35.76 ± 25.44)	22.4 (26.7 ± 19.36)	21.27 (26.95 ± 22.61)	0.484

Values shown as median (mean ± SD). Kruskal–Wallis test across four age groups. Observation time = 255.00 h (Surface Not-In-View excluded).

Object/surface frequency patterns showed significant age-related variation for select categories (Table 5). Pacifier frequency declined significantly with age (Kruskal–Wallis *χ*^2^ = 11.695, df = 3, *p* = 0.009), with complete discontinuation in children 3 to <6 years. Similarly, porous plastic toy frequency showed significant age-related decline (*χ*^2^ = 15.126, df = 3, *p* = 0.002), as did fabric toy (*χ*^2^ = 10.075, df = 3, *p* = 0.018), sticky food (*χ*^2^ = 7.898, df = 3, *p* = 0.048), and plastic tool and appliance (*χ*^2^ = 7.832, df = 3, *p* = 0.050). In each case, *post-hoc* comparisons indicated significantly lower frequency in children 3 to <6 years compared to younger age groups.

Mouthing frequency for food containers, other food, hands, hair and body, and beverage showed no significant differences by age, race, or sex after Bonferroni correction (all *p* > 0.05). Beverage frequency was the only category showing a significant regional difference, with higher rates in North Carolina compared to Arizona (*p* = 0.045).

### 3.4 Time spent in indoor locations (microenvironments)

**Figure 2:**
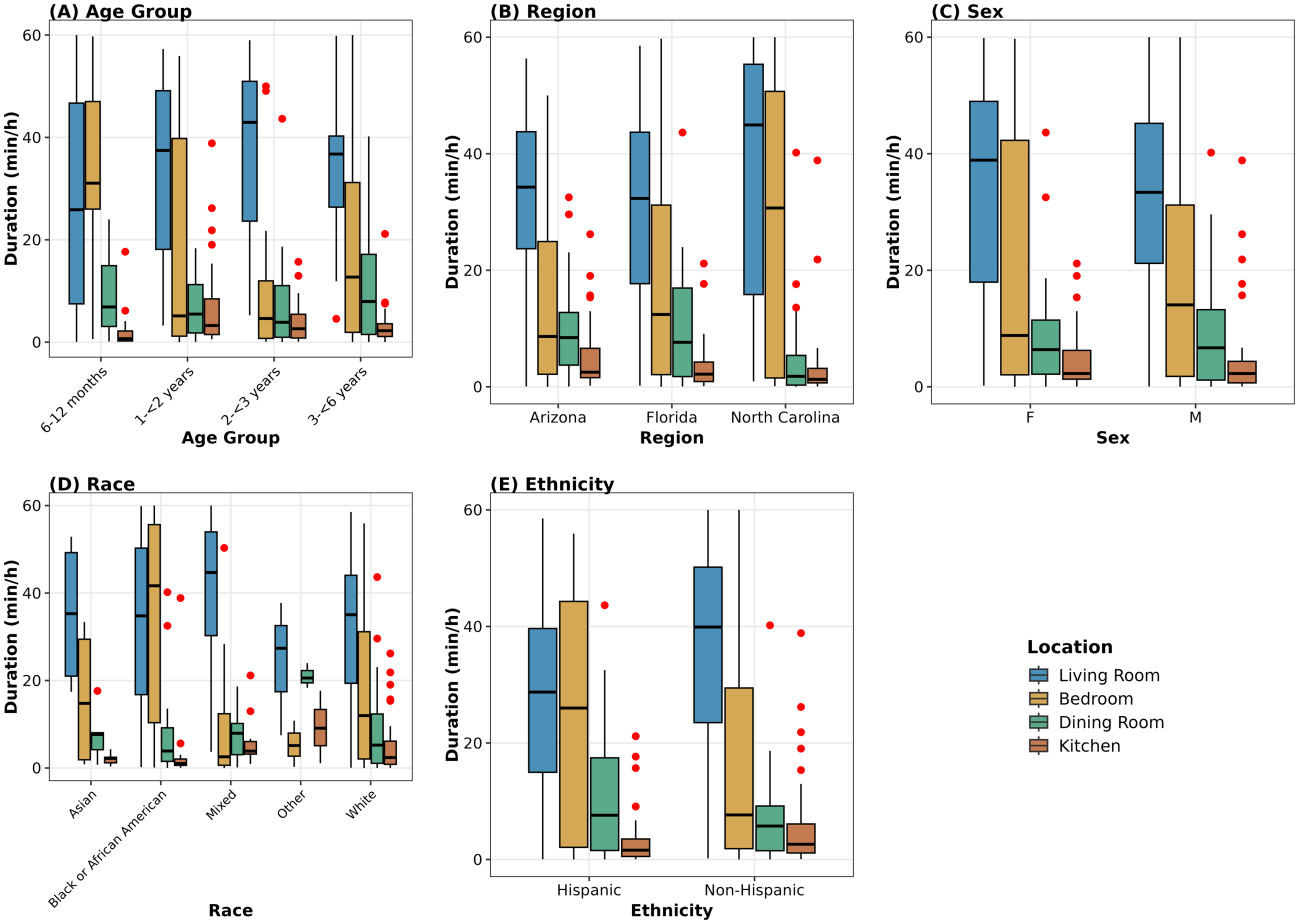
Time spent in household locations (*n* = 99). Box plots show duration (min/h) across the four primary locations (Living Room, Bedroom, Dining Room, Kitchen) stratified by **(A)** age group, **(B)** region, **(C)** sex, **(D)** race, and **(E)** ethnicity. Boxes represent median and IQR (whiskers); extend to 1.5 × IQR; red dots indicate outliers. Living rooms consistently showed the highest duration across all demographic groups. Sample sizes: Age (*n* = 17, 25, 24, 33); Region (*n* = 33 each); Sex (F = 47, M = 51, O = 1); Race (Asian = 6, Black/African American = 25, Mixed = 15, Other = 3, White = 50); Ethnicity (Hispanic = 39, Non-Hispanic = 60). Duration represents total observation time in each location (includes all activities regardless of object contact). Other = 3, White = 50); Ethnicity (Hispanic = 39, Non-Hispanic = 60). One participant reporting ‘Other’ sex (*n* = 1) was included in statistical testing but excluded from the sex panel for visualization due to insufficient sample size.

Children spent the vast majority of observed time in four household locations, which together accounted for 94.57% of in-view observation time. Living rooms were the primary activity area, with a median of 56.78% of in-view observation time per child (mean = 53.56%, SD = 30.39%; 8,595 total minutes). Bedrooms accounted for a median 4.14% of total observation time (4,181 min), dining rooms had a median of 2.89% (1,577 min), and kitchens 3.71% (1,157 min). The remaining time was distributed across playrooms (median = 0.00%, 514 min), corridors (median = 0.60%, 249 min), and other locations (Table 6; Figure 2).

**Table 6:** Location time spent statistical tests by demographics.

Demographic	Location	Statistic	df	*p*-value	Sig
Age	Living Room	3.537	3	0.3159	ns
	Bedroom	9.326	3	0.0253	*
	Dining Room	5.517	3	0.1377	ns
	Kitchen	6.259	3	0.0997	ns
	Corridors	13.971	3	0.0029	**
	Playroom	0.582	3	0.9005	ns
	Balcony/Porch	5.442	3	0.1422	ns
Region	Living Room	1.348	2	0.5096	ns
	Bedroom	3.06	2	0.2166	ns
	Dining Room	10.472	2	0.0053	**
	Kitchen	9.935	2	0.0070	**
	Corridors	10.562	2	0.0051	**
	Playroom	1.616	2	0.4458	ns
	Balcony/Porch	1.308	2	0.5200	ns
Sex	Living Room	2.034	2	0.3617	ns
	Bedroom	2.504	2	0.286	ns
	Dining Room	2.756	2	0.252	ns
	Kitchen	4.817	2	0.0899	ns
	Corridors	3.248	2	0.1971	ns
	Playroom	1.222	2	0.5428	ns
	Balcony/Porch	1.342	2	0.5111	ns
Race	Living Room	4.168	4	0.3838	ns
	Bedroom	3.821	4	0.4308	ns
	Dining Room	10.897	4	0.0277	*
	Kitchen	12.797	4	0.0123	*
	Corridors	4.446	4	0.3490	ns
	Playroom	1.199	4	0.8783	ns
	Balcony/Porch	4.359	4	0.3596	ns
Ethnicity	Living Room	844.5	—	0.0199	*
	Bedroom	1,300	—	0.3476	ns
	Dining Room	1512.5	—	0.0130	*
	Kitchen	1,068	—	0.4670	ns
	Corridors	963	—	0.1367	ns
	Playroom	1,228	—	0.4676	ns
	Balcony/Porch	1,176	—	0.9400	ns

Kruskal–Wallis test for ≥3 groups; Mann–Whitney U for 2 groups. *p < 0.05, **p < 0.01, ***p < 0.001.

Time spent indoors differed significantly across locations (Kruskal–Wallis, *p* < 0.001), with living rooms differing significantly from all other locations (all adjusted *p* < 0.001).

### 3.5 Time spent (durations) in indoor locations by demographics

Significant age-related differences were observed for bedroom duration (Kruskal–Wallis *χ*^2^ = 9.326, df = 3, *p* = 0.025) and corridor usage (*χ*^2^ = 13.971, df = 3, *p* = 0.003). Median bedroom duration was 28.26 min/h in children 6 to <12 months compared to 0.33 to 5.50 min/h in older age groups. However, no significant differences between specific age groups were observed (all adjusted *p* > 0.099), suggesting that children 6 to <12 months spent more time in bedrooms, likely reflecting daytime napping. It should be noted that video recordings were conducted during daytime waking hours only; some children napped during recording sessions, but nighttime sleep periods were not captured, which may account for the relatively low bedroom durations observed in older age groups. No significant differences were observed for bedroom duration by sex, race, ethnicity, or region (all *p* > 0.05).

Corridor usage increased significantly with age (*p* = 0.003), with median duration increasing from 0.01 min/h in children 6 to <12 months to 0.44–0.56 min/h in older age groups, reflecting increased mobility and independent movement through the home. *Post-hoc* comparisons confirmed significantly higher corridor duration in all older age groups compared to children 6 to <12 months (all *p* < 0.036). Corridor duration also showed significant regional differences (Kruskal–Wallis *χ*^2^ = 10.562, df = 2, *p* = 0.005), with Arizona (median = 0.72 min/h) significantly higher than both Florida (median = 0.12 min/h, *p* = 0.009) and North Carolina (median = 0.18 min/h, *p* = 0.026). Because age distributions across sites were not stratified in this analysis, the regional difference may be partially attributable to differences in the age composition of children enrolled at each site, in addition to potential differences in home layout.

Living room usage showed no significant age differences (Kruskal–Wallis *χ*^2^ = 3.537, df = 3, *p* = 0.316) but differed significantly by ethnicity (Mann–Whitney U = 844.5, *p* = 0.020), with Hispanic children (median = 28.19 min/h) spending less time in living rooms than Non-Hispanic children (median = 39.69 min/h). Hispanic children instead spent significantly more time in dining rooms (median = 4.01 min/h) compared to non-Hispanic children (median = 0.94 min/h), suggesting cultural differences in how household spaces are used for children’s activities.

Dining room duration also differed by race (Kruskal–Wallis *χ*^2^ = 10.897, df = 4, *p* = 0.028) and region (*χ*^2^ = 10.472, df = 2, *p* = 0.005). However, no individual race pairwise comparisons reached significance after Bonferroni correction, likely due to small sample sizes in some racial categories (Other *n* = 3, Asian *n* = 6); median duration ranged from 0.02 min/h in Black or African American children to 20.30 min/h in children classified as Other race. For region, children in North Carolina (median = 0.05 min/h) spent significantly less time than children in Arizona (median = 6.95 min/h, *p* = 0.003). Kitchen duration showed significant differences by race (*χ*^2^ = 12.797, df = 4, *p* = 0.012) and region (*χ*^2^ = 9.935, df = 2, *p* = 0.007). *Post-hoc* comparisons for race revealed that Mixed race children (median = 3.75 min/h) spent significantly more time in kitchens than Black or African American children (median = 0.90 min/h, *p* = 0.047). For region, North Carolina children again showed lower duration than Arizona (*p* = 0.007). Complete distributions are provided in Supplementary Tables S11–S18.

### 3.6 Location frequency

**Figure 3:**
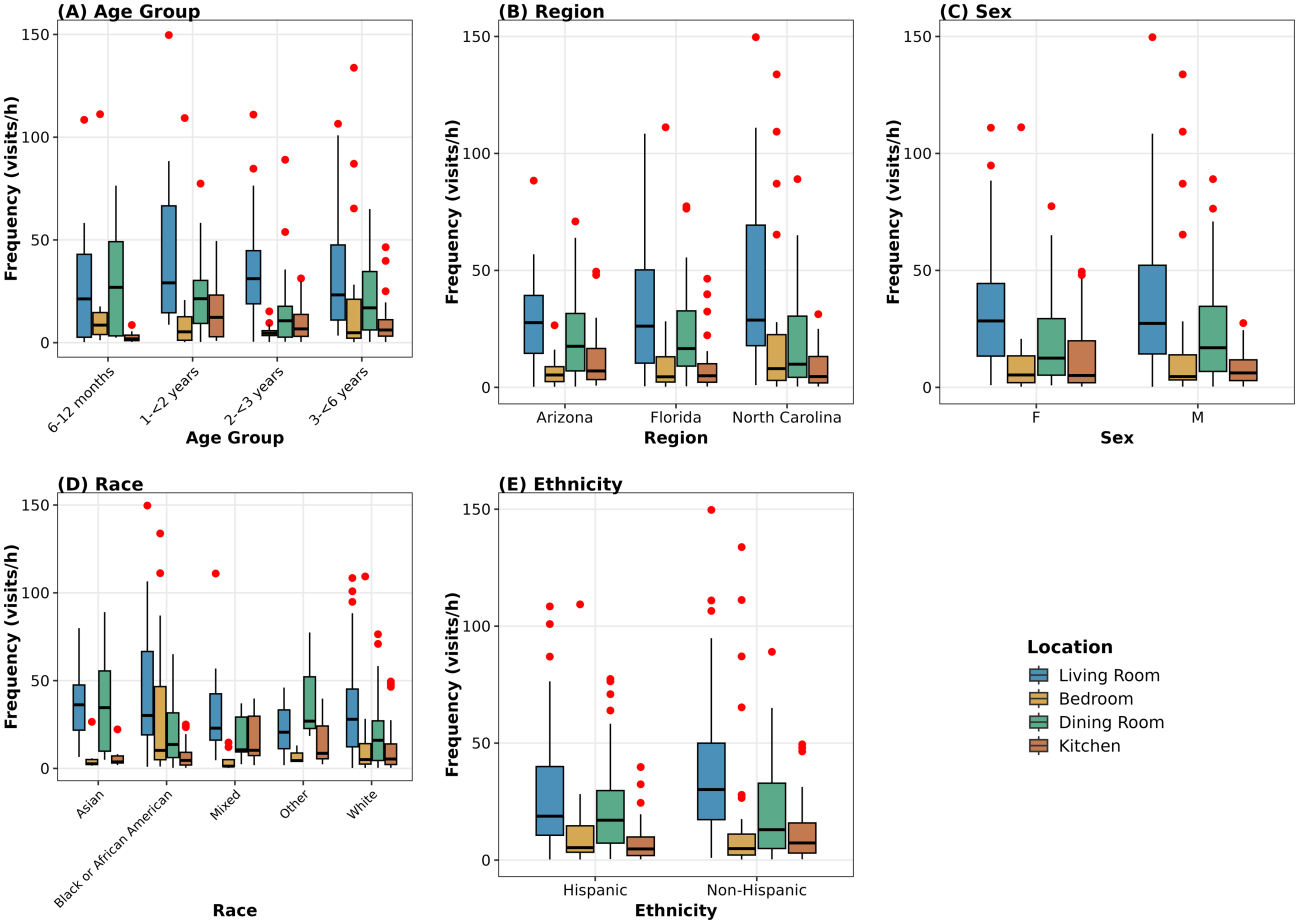
Visitation frequency by household location (*n* = 99). Box plots show visitation frequency (visits/h) across the four primary locations (Living Room, Bedroom, Dining Room, Kitchen) stratified by **(A)** age group, **(B)** region, **(C)** sex, **(D)** race, and **(E)** ethnicity. Boxes represent median and IQR; whiskers extend to 1.5 × IQR; red dots indicate outliers. Living rooms consistently showed the highest visitation frequency across demographic groups. Sample sizes: Age (*n* = 17, 25, 24, 33); Region (*n* = 33 each); Sex (F = 47, M = 51, O = 1); Race (Asian = 6, Black/African American = 25, Mixed = 15, Other = 3, White = 50); Ethnicity (Hispanic = 39, Non-Hispanic = 60). Frequency represents distinct entries into each location. Other = 3, White = 50); Ethnicity (Hispanic = 39, Non-Hispanic = 60). One participant reporting ‘Other’ sex (*n* = 1) was included in statistical testing but excluded from the sex panel for visualization due to insufficient sample size.

Living rooms showed the highest location frequency (median = 27.67 visits/h, mean = 35.13 visits/h, SD = 29.83), followed by dining rooms (median = 7.59 visits/h, mean = 15.26 visits/h, SD = 19.85), kitchens (median = 5.25 visits/h, mean = 9.71 visits/h, SD = 11.95), and bedrooms (median = 3.35 visits/h, mean = 9.94 visits/h, SD = 21.12) (Table 7; Figure 3). The four primary locations accounted for 8,765 visits to living rooms, 3,989 visits to dining rooms, 2,883 visits to bedrooms, and 2,444 visits to kitchens across all children over observation time periods.

**Table 7:** Location visit frequency statistical tests by demographics.

Demographic	Location	Statistic	df	*p*-value	Sig
Age	Living room	3.886	3	0.274	ns
	Bedroom	8.776	3	0.0324	*
	Dining room	4.879	3	0.1809	ns
	Kitchen	13.633	3	0.0034	**
	Corridors	18.73	3	< 0.001	***
	Playroom	0.535	3	0.911	ns
	Balcony/Porch	8.978	3	0.0296	*
Region	Living room	0.738	2	0.6913	ns
	Bedroom	2.588	2	0.2742	ns
	Dining room	5.842	2	0.0539	ns
	Kitchen	5.434	2	0.0661	ns
	Corridors	5.202	2	0.0742	ns
	Playroom	1.49	2	0.4748	ns
	Balcony/Porch	0.144	2	0.9305	ns
Sex	Living room	0.702	2	0.704	ns
	Bedroom	2.456	2	0.2929	ns
	Dining room	3.115	2	0.2107	ns
	Kitchen	3.006	2	0.2224	ns
	Corridors	2.293	2	0.3178	ns
	Playroom	1.248	2	0.5358	ns
	Balcony/Porch	0.67	2	0.7153	ns
Race	Living room	0.511	4	0.9725	ns
	Bedroom	5.087	4	0.2785	ns
	Dining room	9.09	4	0.0589	ns
	Kitchen	5.509	4	0.2389	ns
	Corridors	1.717	4	0.7876	ns
	Playroom	1.334	4	0.8556	ns
	Balcony/Porch	1.724	4	0.7864	ns
Ethnicity	Living room	911.5	—	0.0647	ns
	Bedroom	1,281	—	0.4235	ns
	Dining room	1441.5	—	0.049	*
	Kitchen	1,040	—	0.3534	ns
	Corridors	1,040	—	0.3507	ns
	Playroom	1,225	—	0.4912	ns
	Balcony/Porch	1,168	—	0.9854	ns

Kruskal–Wallis test for ≥3 groups; Mann–Whitney U for 2 groups. *p < 0.05, **p < 0.01, ***p < 0.001.

### 3.7 Location frequency by demographics

Location-specific frequency patterns showed significant age-related differences for bedroom (Kruskal–Wallis *χ*^2^ = 8.776, df = 3, *p* = 0.032), kitchen (*χ*^2^ = 13.633, df = 3, *p* = 0.003), corridor (*χ*^2^ = 18.730, df = 3, *p* < 0.001), and balcony or porch (*χ*^2^ = 8.978, df = 3, *p* = 0.030).

Corridor frequency showed the strongest age effect, increasing from median 0.34 visits/h in children 6 to <12 months to 8.33 visits/h in children 3 to <6 years, reflecting increased independent mobility with age. *Post-hoc* comparisons confirmed significantly higher frequency in all older age groups compared to children 6 to <12 months (all *p* < 0.031). Kitchen frequency was also significantly higher in older children compared to children 6 to <12 months (all pairwise *p* < 0.027). Balcony or porch frequency showed significant differences between children 3 to <6 years and children 2 to <3 years (*p* = 0.037). Dining room frequency differed by ethnicity (Mann–Whitney U = 1441.5, *p* = 0.049), with Hispanic children (median = 13.04 visits/h) making more frequent visits than non-Hispanic children (median = 3.93 visits/h). No significant differences in location-specific frequency were observed for living room, playroom, den, bathroom, or laundry room by any demographic variable (all *p* > 0.05). Complete distributions are provided in Supplementary Tables S11–S19.

### 3.8 Duration of object and surface contact within locations

**Figure 4:**
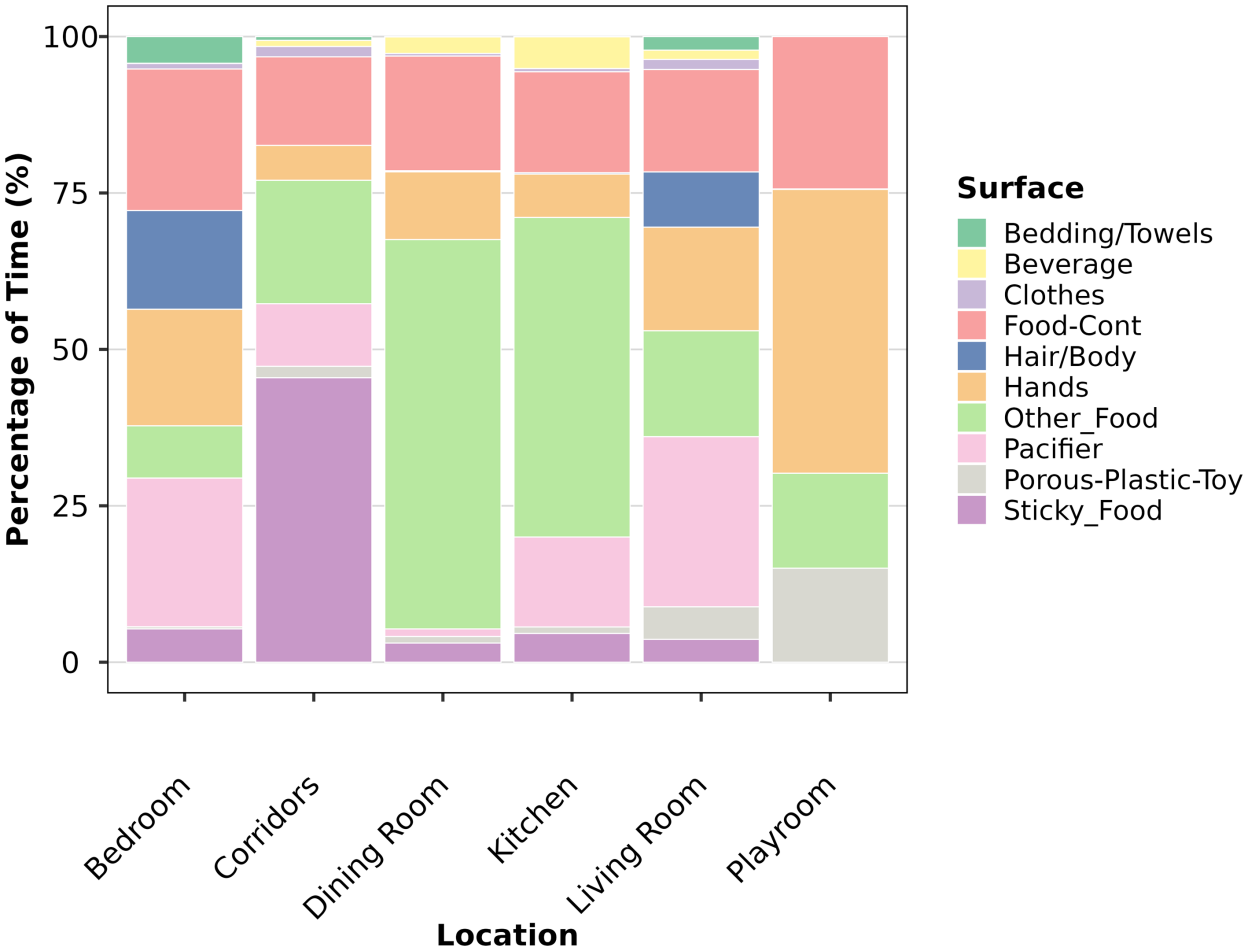
Surface-specific mouthing duration by household location (*n* = 99). Stacked bar chart shows the distribution of mouthing time across surfaces within each household location. Within each major location, surfaces differed significantly in mouthing time: living room (Kruskal-Wallis *χ*^²^ = 273.054, df = 9, *p* < 0.001), bedroom (*χ*^²^ = 103.981, df = 9, *p* < 0.001), dining room (*χ*^²^ = 255.32, df = 9, *p* < 0.001), kitchen (*χ*^²^ = 118.15, df = 9, *p* < 0.001), and corridors (*χ*^²^ = 16.54, df = 8, *p* = 0.035). Each color represents a different surface category. Food-cont = Food containers. Sample size: *n* = 99 children.

Chi-square analysis revealed that surface mouthing patterns were significantly associated with household location (*χ*^2^ = 1398.68, df = 45, *p* < 0.001), indicating that children mouth different surfaces depending on which room they occupy (Table 8; Figure 4). Within each major location, surfaces differed significantly in mouthing duration (all *p* < 0.05 except playroom, *p* = 0.100). Living rooms showed the highest total mouthing duration and greatest surface diversity, with pacifier (365 min), other food (227 min), hands (222 min), and food containers (220 min) as the dominant surfaces. Bedrooms showed substantial pacifier use (199 min) and food container contact (189 min). Dining rooms and kitchens were dominated by food-related surfaces, with “other food” accounting for the highest single-location food mouthing duration in dining rooms (253 min). Corridors and playrooms showed minimal overall mouthing duration (see Supplementary Tables S20, S21 for statistical tests of surface mouthing differences across locations, and Supplementary Table S22 for statistical tests of location mouthing differences across surfaces).

**Table 8:** Top 15 surface × location combinations by total mouthing duration.

Rank	Surface	Location	Duration (min)	% of Total Time	Frequency	*N* subjects
1	Pacifier	Living Room	365.15	2.23	113	10
2	Other_Food	Dining Room	253.05	1.54	757	44
3	Other_Food	Living Room	227.27	1.39	672	55
4	Hands	Living Room	222.18	1.36	881	73
5	Food-Cont	Living Room	219.80	1.34	654	51
6	Pacifier	Bedroom	199.00	1.21	54	7
7	Food-Cont	Bedroom	189.35	1.16	142	19
8	Hands	Bedroom	155.95	0.95	458	34
9	Hair/Body	Bedroom	131.93	0.81	58	13
10	Hair/Body	Living Room	118.38	0.72	106	21
11	Other_Food	Kitchen	93.10	0.57	214	33
12	Food-Cont	Dining Room	74.57	0.46	597	35
13	Other_Food	Bedroom	69.83	0.43	170	12
14	Porous-Plastic-Toy	Living Room	69.82	0.43	226	28
15	Sticky_Food	Living Room	49.05	0.3	164	15

Duration and frequency calculated from active mouthing events (excluding Nothing and Not-In-View).

## 4 Discussion

### 4.1 Summary of key findings

This study provides comprehensive quantitative data on indoor mouthing behaviors in a large sample of children 6 months to 6 years, across three geographically distinct U.S. regions using advanced 360 degree camera technology. Our study’s use of multiple 360-degree cameras for extended observation periods using mounted and unattended cameras in home settings, combined with systematic video review using standardized translation protocols, provides comprehensive detection of mouthing behaviors across diverse household locations and activities.

The methodology captured natural play behaviors without direct researcher presence, addressing some limitations of traditional videotaping approach of following subjects with cameras (24, 39). Across 99 participants, 255 h of usable video footage were analyzed, revealing that children spent 18.74% of in view observation time mouthing surfaces or objects (with the remaining 81.26% classified as “nothing,” indicating the child’s mouth was visible but no mouthing contact occurred).

The key finding of significant age-related differences in mouthing duration (*p* = 0.007) but not frequency (*p* = 0.497) has important implications for exposure assessment. Median mouthing duration declined from 18.7 min/h in children 6 to <12 months to 5.02 min/h in children 3 to <6 years, while median frequency remained relatively stable across age groups (21.3 to 27.8 contacts/h). This pattern suggests that younger children maintain longer contact with mouthed objects despite similar contact frequencies across ages. This pattern of declining duration with stable frequency is consistent with findings across multiple populations. Lopez-Galvez et al. reported significant age-related differences in mouthing duration but not always in frequency among children aged 1–12 years (4). Beamer et al. found markedly reduced mouthing duration (median 0.4 min/h for hands) but persistent mouthing frequency (12.6 events/h) in children aged 7–12 years outdoors (42). Kwong et al. (15), in a longitudinal study of 30 Bangladeshi children observed over 3 years, also documented age-related variation in mouthing frequency. This consistency across diverse populations and methodologies reinforces the robustness of this developmental trend. This indicates and emphasizes that duration may be more relevant for assessing chemical absorption, as contaminants such as flame retardants and heavy metals are released gradually from surfaces through sustained contact while frequency may better predict pathogen transfer, as microbial exposure occurs rapidly upon initial contact with each new touch representing a discrete exposure event (29, 41).

The observed age-related decline in mouthing duration, combined with the persistence of mouthing frequency, also reflects well-established developmental trajectories. Mouthing serves as a dual role in early childhood: it is both an exposure pathway for environmental contaminants and a normative developmental behavior that infants and young toddlers explore the sensory properties of objects in their environment (42, 44). During the first year of life, oral exploration is a primary mode of learning about object texture, shape, and temperature. Additionally, mouthing episodes tend to be prolonged as infants engage in sustained sensory investigation. As children mature, the development of fine motor skills, language, and visual-manual exploration provides alternative means of investigating objects, reducing the need for prolonged oral contact while maintaining brief, habitual hand-to-mouth and object-to-mouth contacts (43, 44). This developmental context helps explain why duration declines sharply with age while frequency remains relatively stable. Older preschool children continue to touch objects to their mouths at similar rates, but these contacts become increasingly brief and habitual rather than exploratory in nature. Recognizing this developmental dimension is important for exposure assessment because it suggests that the elevated mouthing durations observed in the youngest children are not merely statistical outliers but reflect a biologically driven behavioral pattern that predictably intensifies contaminant exposure during a critical window of physiological vulnerability.

### 4.2 Mouthing frequency comparison with previous studies

Previous mouthing behavior studies, both those reviewed by the EPA Exposure Factors Handbook (2011) (45) and more recent investigations (12, 15, 20, 46), had notable limitations in sample size, observation duration, or population diversity. Our study (*n* = 99) addresses these EPA-identified limitations with the largest sample size among video-based indoor mouthing studies, observation periods of 2.4 to 2.9 h of usable footage per child (mean observation time without periods when children were not in view: 2.87 ± 0.82 h for children 6 to <12 months; 2.39 ± 0.79 h for children 1 to <2 years; 2.57 ± 0.75 h for children 2 to <3 years; and 2.57 ± 0.84 h for children 3 to <6 years), and geographic diversity across three U.S. regions (Arizona, Florida, North Carolina).

Our median total mouthing contact frequencies for children across age groups ranged from 21.3 to 27.8 contacts/h (mean values: 26.8–35.5 contacts/h), which can be directly compared with findings from previous studies using diverse methodologies and populations (Supplementary Table S3). For children 6 to <12 months, we observed a median frequency of 23.5 contacts/h and a mean of 28.6 ± 25.0 contacts/h. Our mean is comparable to the median value of 34.4 contacts/h reported by Kwong et al. for 6–12 month old children in rural Bangladesh using 5-h structured observations (i.e., notetaking) (1). Beamer et al. reported object-to-mouth frequencies for 23 farmworker children aged 6–27 months in Salinas Valley, California, with an overall median of 27.2 contacts/h and mean of 29.2 contacts/h, and an overall hand-to-mouth median of 15.2 contacts/h and mean of 18.4 contacts/h. Age-stratified object-to-mouth frequencies were higher for infants (6–13 months: mean = 37.8, median = 35.2 contacts/h) than toddlers (20–27 months: mean = 18.0, median = 15.9 contacts/h). Direct comparison with our total frequencies requires caution, as our values include food, hands, and beverages alongside non-dietary objects, whereas Beamer et al. reported these categories separately. The higher object-to-mouth frequencies in Beamer et al. may reflect their focus on farmworker children in agricultural settings, where increased environmental exposures and outdoor play could promote more frequent mouthing behaviors (40).

For children 1 to <2 years, our median of 27.8 contacts/h (mean = 35.5 ± 25.1 contacts/h) is higher than values reported by Freeman et al., who found a median of 3.5 contacts/h for hand-to-mouth and 3 contacts/h for object-to-mouth (approximately 6.5 contacts/h combined) for children 3 to 4 years, though direct comparison is limited by their older age grouping (35).

For children 2 to <3 years, our median of 22.5 contacts/h (mean = 26.8 ± 19.4 contacts/h) is higher than values reported in several studies. AuYeung et al. reported substantially lower contact frequencies for children >24 months in primarily outdoor settings (median = 14.6 contacts/h), likely reflecting the outdoor focus of their observations (47). Black et al. reported a median of 15.4 contacts/h for 25–36 month old children in an agricultural community on the US/Mexico border in south Texas. Differences between our findings and Black et al. may reflect methodological variations, including observation setting (indoor-only versus indoor and outdoor), camera technology, and data collection approaches (36).

For children 3 to <6 years, our median of 21.3 contacts/h (mean = 27.00 ± 22.70 contacts/h) is higher than most previous reports for this age group. Reed et al. reported a mean of 9.5 contacts/h (median 8.5 contacts/h) for hand-to-mouth contacts among children 2–6 years in daycare and residential settings in New Jersey (34). Freeman et al. found a median of 3 contacts/h for hand-to-mouth (with 0 object-to-mouth contacts) for 7–8 year old children in Minnesota, substantially lower than our findings for younger preschool children (35). The higher frequencies we observed may reflect our video review methodology’s superior detection of brief mouthing events compared to real-time observation methods used in some earlier studies, as well as potential differences in children’s behavior during natural home observation versus more structured daycare settings.

International studies provide additional context for our frequency findings. Kwong et al., in a longitudinal study of 30 Bangladeshi children under 4 years observed for 6 h annually over 3 years (15), reported median hand-mouthing frequencies of 43–72 contacts/h and object-mouthing frequencies of 17–34 contacts/h, substantially higher than our U.S.-based estimates. In contrast, Tsou et al. reported lower median indoor frequencies among 24 Taiwanese children aged 3 to <6 years (hand-to-mouth = 10 contacts/h, object-to-mouth = 4.3 contacts/h) (16). Wilson et al. observed hand-to-face contacts among 263 individuals including children in daycare, finding that contact patterns differed significantly between eating and non-eating activities (46). Using a computer vision approach, Lupolt et al. reported a mean object-to-mouth contact rate of 27 contacts/h among 25 children aged 6–18 months in Baltimore, comparable to our estimates for the youngest age group (20). These comparisons highlight the influence of population, setting, and methodology on mouthing frequency estimates.

### 4.3 Mouthing duration comparisons with previous studies

Quantifying mouthing duration presents distinct methodological challenges compared to frequency measurement. Earlier parent-observation studies reviewed by the U.S. EPA Exposure Factors Handbook (2011) suffered from imprecise timing methods, including the use of minute-level rather than second-level resolution (38) and technical problems with stopwatches affecting 39% of parent observations (48). More recent studies have addressed some of these limitations but introduced others. Kwong et al. conducted 6-h longitudinal video observations of 30 Bangladeshi children, providing duration data across a broader developmental window, but results may not generalize to U.S. populations given differences in indoor environments and play materials (15). Tsou et al. contributed duration data for 24 Taiwanese children aged 3 to <6 years using video-based methods comparable to those used in U.S. studies, but sample sizes remained small (16). Lupolt et al. advanced duration measurement by applying computer vision to automatically detect and time microactivity contacts for 61 children aged 6 to ≤18 months, achieving frame-level temporal resolution that eliminates human coding error, though their observations were limited to 20-min recording sessions (19). Sugeng et al. linked mouthing behavior frequency to flame retardant hand wipe levels in Dutch toddlers, demonstrating the exposure relevance of mouthing duration data but without reporting comprehensive duration estimates (17). Collectively, these studies highlight the persistent need for large-sample, extended-observation duration data across diverse age groups, a gap our study was specifically designed to address.

Among the earliest video-based duration studies, Zartarian et al. reported children mouthed non-dietary objects an average of 4.35% of total observation time (approximately 2.6 min per hour) but studied only 4 children (33). Our study substantially expands on the existing literature, with median total mouthing durations ranging from 5.02 to 18.67 min per hour across age groups (mean values: 8.66–16.83 min per hour), measured across extended multi-hour recording sessions using systematic video translation protocols that ensure consistent timing accuracy through frame-by-frame review (Supplementary Table S1).

For children 6 to <12 months, our median duration of 18.7 min per hour (mean = 16.8 ± 8.55 min per hour) is substantially higher than Beamer et al., who reported non-dietary object mouthing duration for infants aged 6–13 months of mean (4.5 min/h) and median (2.8 min/h). As with frequency comparisons, direct comparison requires caution since our total mouthing duration includes food, hands, and beverages, whereas Beamer et al. reported non-dietary objects separately (40). For children 12 to <24 months (1 to <2 years), our median duration of 8.75 min per hour (mean = 11.59 ± 10.41 min per hour), was lower than those reported by Groot et al. who reported mean values of 44.0 min per day for 6–12 month children and 16.4 min per day for 12–18 month children. However, direct comparison with Groot et al. is complicated by their extrapolation methodology and exclusion of pacifier use (48).

For children 24 to <36 months (2 to <3 years), our median duration of 8.87 min per hour (mean = 11.89 ± 12.90 min per hour) exceeds Beamer et al., who reported non-dietary object mouthing duration for toddlers aged 20–27 months of mean = 2.1 min/h and median = 1.3 min/h. However, when all objects including food and hands are considered, Beamer et al. reported a mean total mouthing duration of 9.6 min/h across all children aged 6–27 months (40), which is more comparable to our values that also include food, hands, and beverages. Similarly, for children 3 to <6 years, our median duration of 5.02 min per hour (mean = 8.66 ± 9.19 min per hour) is generally higher than previous reports. AuYeung et al. reported median outdoor mouthing durations of 0.6 min per hour for children >24 months (47), substantially lower than our findings, likely reflecting their outdoor focus where mouthing behaviors are briefer than in indoor home environments.

### 4.4 Objects/surfaces specific patterns

Mouthing duration differed significantly across surfaces/objects (Kruskal–Wallis *χ*^2^ = 1442.578, df = 26, *p* < 0.001). Four surfaces accounted for the majority of mouthing duration: “other food,” “pacifiers,” “food containers,” and “hands.” This concentration suggests that interventions focused on these pathways may address a substantial portion of non-dietary ingestion exposure (14). The developmental progression from “food containers” and pacifier reliance in younger children to increased “other food” mouthing in older children indicates that age-appropriate interventions should target different exposure pathways. Food containers and utensils can accumulate household dust and residues that transfer to children’s mouths during contact, making this pathway particularly important for infant exposure models (3). The shift toward direct food mouthing in older children represents a change in the dominant exposure pathway (12).

Additionally, toy mouthing duration and frequency both declined with age, reflecting developmental changes in exploratory behaviors (10), while hand mouthing remained stable throughout early childhood (41). Pacifier use showed complete discontinuation by age 3 to <6 years, consistent with typical weaning patterns (13).

### 4.5 Location specific patterns

Previous micro-activity studies have tracked locations visited by children during videotaped observations (40), identifying nine location categories including indoor spaces (bathroom, bedroom, kitchen, living room/den) and outdoor areas (garage, patio, street/sidewalk, vehicle, yard). Our study examined mouthing patterns across 11 indoor location categories, providing finer resolution of exposure-relevant environments.

Living rooms served as the primary location where children spent time during observations (median = 56.78% of observation time, mean = 53.56%), consistent with living spaces being central activity areas in residential settings. Location-specific patterns showed significant age-related differences in bedroom usage (*p* = 0.025) and corridor usage (*p* = 0.003), highlighting the importance of microenvironment-specific exposure assessments (28). These findings can guide targeted dust sampling strategies and inform where exposure reduction efforts should be concentrated, with living rooms accounting for the majority of children’s time during daytime observation periods (11).

The significant association between surface mouthing patterns and household location (*χ*^2^ = 1398.68, df = 45, *p* < 0.001) indicates that children mouth different surfaces depending on which room they occupy. Dining rooms and kitchens were dominated by food-related surfaces, while living rooms and bedrooms showed more diverse surface profiles. This finding has practical implications for exposure assessment, suggesting that contaminant sampling should account for room-specific mouthing patterns rather than assuming uniform exposure across household locations (10).

Overall mouthing duration and frequency showed limited demographic variation in this sample beyond age, with no significant differences detected by sex, race, ethnicity, or geographic region for total mouthing metrics. However, these null findings should be interpreted cautiously given the limited statistical power for some demographic subgroups (Asian *n* = 6, Other race *n* = 3) and the number of comparisons conducted. Location-specific analyses revealed some demographic differences that may warrant further investigation. Dining room duration differed by race (*p* = 0.028) and region (*p* = 0.005), kitchen duration differed by race (*p* = 0.012) and region (*p* = 0.007), and living room duration differed by ethnicity (*p* = 0.020). These location-specific patterns should be interpreted with particular caution, as they may reflect the specific floor plans, room configurations, or household routines of a small number of participating families rather than broad demographic behavioral trends (16, 38). Larger studies with more balanced demographic representation would be needed to determine whether these location-specific patterns are robust and generalizable. Until such data are available, age-stratified exposure factors without additional demographic stratification may be a reasonable default for most exposure modeling applications, while acknowledging that location-specific demographic heterogeneity remains plausible and relevant for micro environmental exposure modeling.

### 4.6 Individual variation

The substantial individual variation observed (maximum values reaching 51.58 min/h duration and 104.78 contacts/h frequency) indicates that population level estimates may inadequately characterize high exposure individuals who warrant special attention in risk assessments (22). Some children exhibited mouthing durations more than five times higher than the mean for their age group. This variability has important implications for protecting the most vulnerable children and suggests that exposure assessments should consider not only central tendency measures but also upper percentile values when establishing safety standards (2, 49).

### 4.7 Limitations

This study has several limitations that should be considered when interpreting the findings. First, the videotaping methodology, while minimizing direct researcher interference, may have introduced some degree of observational bias. Families were aware of camera placement and recording, which could have influenced children’s behavior or parental supervision patterns, potentially leading to more cautious behavior than would occur during completely unobserved play (24). However, the 3–4 h recording duration was designed to allow families to acclimate to the camera presence and resume normal routines.

Technical limitations of the camera equipment presented several challenges. The Insta360 One X2 cameras recorded in 90 min intervals rather than continuously, creating potential gaps in data collection if cameras failed to restart properly. Additionally, the cameras lacked built in lighting capabilities, which compromised video quality in dimly lit environments. Some parts of footage were rendered unusable due to inadequate lighting conditions, potentially underestimating mouthing behaviors that occurred in darker environments such as bedrooms during nap times. This limitation may result in systematic under-sampling of bedroom environments, particularly during nap times and bedtime periods when prolonged pacifier use, thumb-sucking, or blanket mouthing are most likely to occur. Consequently, the bedroom mouthing durations reported in this study may underestimate true values, and the relative contribution of bedrooms to total mouthing exposure may be greater than our data suggest. Future studies should employ cameras with infrared or low-light capabilities to capture behavior in dimly lit environments more completely.

Equipment disconnection posed another significant challenge. In some households, camera power plugs were accidentally or intentionally disconnected during the observation period, resulting in loss of footage. These interruptions were unpredictable and varied across households, leading to unequal observation times.

An important methodological consideration concerns the exclusion of “Not-In-View” periods from the denominator when calculating hourly mouthing rates. This approach assumes that mouthing behavior during periods when the child’s mouth was not visible to the camera was comparable to behavior during visible periods. If children were more likely to turn away from the camera during certain activities (e.g., eating while facing a wall, exploring restricted areas), the excluded periods could systematically differ from observed periods. In particular, if children engaged in more mouthing when turned away (e.g., mouthing forbidden objects) or less mouthing (e.g., during passive activities like watching television from an angle not captured by the camera), the estimated rates could be biased in either direction. However, given that 360-degree cameras were used and multiple cameras were placed throughout the home to maximize coverage, the proportion of Not-In-View time attributable to systematic behavioral selection rather than random camera positioning is likely limited. Nevertheless, future studies should examine whether mouthing rates differ systematically as a function of the proportion of Not-In-View time per child to assess the sensitivity of results to this assumption.

The sample size, while adequate for detecting major age-related differences, may have been insufficient to detect smaller demographic effects. The small numbers in some demographic subgroups (Asian *n* = 6, Other race *n* = 3, Other sex *n* = 1) limit the generalizability of findings for these populations and preclude meaningful statistical comparisons. Location-specific demographic findings (e.g., racial or ethnic differences in dining room or kitchen usage) should be interpreted with particular caution, as these patterns may reflect the specific floor plans and household configurations of a few participating families rather than generalizable behavioral trends. With only 33 households per region and uneven racial/ethnic distributions within regions, apparent demographic effects on room-level behavior could be confounded with housing characteristics.

Lastly, the cross-sectional design captures mouthing behavior at a single time point, which may not represent typical behavior patterns for individual children (28). Day-to-day variability in activity patterns, food consumption, and play preferences likely affect mouthing behaviors, and longitudinal observations would be needed to characterize within-child variation and identify stable behavioral patterns versus episode-specific contacts.

### 4.8 Recommendations

Based on the results obtained in this study, we offer several recommendations for exposure assessment and future research. First, exposure assessment models should incorporate age specific mouthing duration parameters in addition to frequency data. Previous meta-analyses have focused primarily on contact frequency (29, 41), but our findings demonstrate that duration and frequency provide distinct information relevant to different exposure scenarios. The distinction is critical: prolonged contact duration may facilitate greater chemical absorption through dermal or oral routes, while contact frequency may be more relevant for pathogen transfer or acute exposures (14).

Second, the dominance of specific surfaces with “other food,” “pacifiers,” “food containers,” and “hands” collectively accounting for the majority of total mouthing time suggests that surface specific parameters should be incorporated into exposure calculations rather than assuming uniform contact patterns across all mouthed objects (10). Furthermore, exposure assessors should explicitly distinguish dietary from non-dietary mouthing pathways when selecting input parameters, as these pathways may be defined differently depending on the modeling framework (50); the dietary and non-dietary surface contributions reported in Supplementary Tables S5–S9 provide demographic-specific values that can be selected to match the chosen definition.

Third, location specific exposure assessments should be considered, particularly for contaminants that accumulate differentially across household microenvironments (11). Living rooms, as the primary activity location (median 56.78% of total observation time), should be prioritized for dust sampling in exposure studies.

Fourth, future research should address the technical limitations encountered with camera equipment. Studies should employ systems with extended continuous recording capability, integrated lighting or infrared sensors, and secure power solutions to prevent data loss from accidental disconnection.

Fifth, research should expand beyond three U.S. regions to capture greater geographic and cultural diversity in mouthing behaviors. Studies from Bangladesh, Taiwan, the Netherlands, and Korea have documented mouthing behaviors across diverse settings, suggesting that U.S.-derived parameters may not be generalizable to all populations (1, 12, 15–17, 51). Advances in computer vision methodology offer promising approaches for reducing the labor-intensive burden of manual video coding and scaling data collection across populations (19, 20). Longitudinal designs with multi-day observations would be better characterized within individual variability (21).

Sixth, future analyses of this dataset should exploit the sequential MLATS structure to examine mouthing bout organization, transition patterns between rooms and surfaces, and within-child temporal clustering of contacts. Lastly, future studies should validate these behavioral observations against biomarker measurements to confirm their relevance for actual contaminant exposure (12, 13). Integration with environmental sampling data would strengthen the utility of these findings for risk assessment applications.

## 5 Conclusion

This study provides comprehensive, age-specific mouthing contact data for children 6 months to 6 years that addresses critical gaps in exposure assessment research. Key findings include: (1) mouthing duration decreases significantly with age (median declining from 18.67 min/h in infants to 5.02 min/h in preschoolers) while frequency remains stable across age groups (median 21.3 to 27.8 contacts/h); (2) four surfaces, “Other food,” “Pacifiers,” “Food containers,” and “Hands,” account for the majority of mouthing duration; (3) pacifier use shows complete discontinuation by age 3 to <6 years; and (4) no significant differences in total mouthing duration or frequency were observed across sex, race, ethnicity, or geographic region, suggesting age-stratified exposure factors may be sufficient for most applications.

The distinction between duration and frequency has important implications for exposure modeling. Our finding that duration decreases significantly with age while frequency remains stable suggests that current models using frequency alone as a proxy for exposure may not adequately capture age-related differences in actual contaminant intake (29, 41). Models should incorporate both parameters, with duration potentially weighted more heavily for exposures involving chemical absorption and frequency weighted more heavily for pathogen transfer or acute exposures. These findings, consistent with patterns reported across diverse populations and methodologies (4, 15, 19), contribute essential empirical data for reducing uncertainty in exposure models such as the SHEDS-Soil/Dust model (30) and for updating the Exposure Factors Handbook.

## Data Availability

The raw data supporting the conclusions of this article will be made available by the authors upon request.
