# Supplementary material for: Analysis of durations and frequencies of mouthing behaviors in children 6 months to 6 years: an advanced videotaping and translation approach
Source: Front Public Health. 2026 May 12;14:1817680. doi: 10.3389/fpubh.2026.1817680 (PMC13220778; doi:10.3389/fpubh.2026.1817680)
Supplement: Supplementary file 1 [file Supplementary_file_1.docx]

**S1 Figure: Mouth Translation Palette on Virtual Timing Device (VTD)**

**Supplemental Table S1. Categorization of objects and surfaces**

| **Categories** | **Objects/Surfaces** |
| --- | --- |
| Hard Toy | Coins/Pens, Bike, Ride-in Cars, Magnet/Sunglasses/Hair Clips |
| Porous Plastic Toy | Crayon, Balloon, Plastic Pencil, Plastic Toys |
| Fabric Toy | Baseball Glove (leather), Baseball (synthetic leather w/seams), Paper Products Used as Toys |
| Wood Toy | Wood Toy |
| Plastic Tool/Appliance | Scotch Tape, Duct Tape, Rubbermaid Tub, Electric Cord, Home System, Fake Plants, Fridge, Stove, Body Wash Bottles, Shampoo Bottles, Trowel Handle, Sandpaper, Cardboard Boxes, Ply Board, Drop Cloth, Wood Plank/Board, Broom, Clothing Hangers, Sledge Hammer |
| Metal Tool/Appliance | Metal Tool/Appliance |
| Plastic Wall/Furniture | Kiddy Pool, Swing Seat, Volley Ball Net Poles, Toy Box and Lid, Plastic Counter, Plastic Slide/Play Structure, Strings Holding Game Net Down, Volley Ball Netting (plastic coated), Rubbermaid Container Cover, Plastic Coating on Chain Swing, Plastic Plant Hanger, Tent |
| Metal Wall/Furniture | Metal Play Structure, Screen Door, Handle to Turn Hose On, Trampoline Frame, Glass Doors, Railings of Staircase, Handle of Drawers, Arm Rest on Bench, Glass on Cabinet, Glass Doorknob, Tile Table Top, Metal Nails on Play Gym, Sink, Dog Cage, Glass Table Top, Tile Sink Top, Metal Post |
| Fabric Wall/Furniture | Leather/Fabric Couches, Rope Swing, Fabric Seat on Chair, Play Tent/House Rope, Paint Drop Cloth, Soccer Net (rope), Basketball Net |
| Wood Wall/Furniture | Wood Siding on Play Center, Wood Door, Dining Table, Wood Jungle Gym, Picnic Table, Tree House, Wood Chair, Wood Bench, Tree Stumps (used as seats) |
| Rock/Brick Wall/Furniture | Marble Counter Top, Brick Wall, Concrete Planters, Concrete Wall, Columns/Pillars |
| Carpet/Mat | Carpet/Mat |

**Supplemental Table S1, Continued**

| **Categories** | **Objects/Surfaces** |
| --- | --- |
| Food Container | Straw/Wrapper/Hamburger Wrapper/Candy Wrapper, Lollipop Stick, Ziploc Sandwich Bag, Salad Dressing Seasoning Pack, Sipping Cups, Gym Bottles, Drink Cans, Shaker Bottles, Feeding Bottles, Halloween Candy Bag |
| Other Food | All Other Types of Food Except Sticky Foods |
| Sticky Food | Candy, Lollipop, Fruits |
| Beverage | Soda |
| Hair/Body | Skin (not nose or eyes), Face (not nose or eyes) |
| Hands | Hands |
| Nose | Nose |
| Pacifier | Pacifier |
| Bedding/Towels | Curtains, Towels, Kids Blanket, Comforters, Bedsheet, Luffa |
| Clothes | Shoes, Hangers, Socks, Shoelaces, Gym Bag, Clothing |
| Footwear | Footwear |
| Paper/Wrapper | Tissue Paper, Book/Notebook, Greeting Card, Band-Aids, Paper Towel, Stickers/Matches, Paper Money, Playing Cards/Newspapers, Paper Bag |
| Nothing | No object/surface contact observed |
| Animal | Dog Leash, Dog Bone, Dog Toy, Dog Collar, Dog Feces, Sock Dog Plays With |
| Vegetation | Flower, Plants, Leaves |
| Electronics | Television, Computers, iPad, Computerized Toys, Game Systems, Phone, Sign on Play-center |
| Water | Hand Sanitizers, Washing Hands, Soap, Bubbles |

*Note: Categories correspond to the object/surface types coded during 360° video translation.*

*Items within each category represent specific objects observed during in-home observations.*

## **Supplemental Table S2. Per-Child Observation Time and Not-In-View Summary (n = 99)**

Surface analysis: Total time available for surface coding (hours), in-view time when mouth was visible and

surface coding was possible (hours), and Surface Not-In-View time when mouth was not visible (hours, %).

Location analysis: Total observation time (hours), In-View time when child was in a room with camera coverage (hours),

and Location Not-In-View time when child was in a room without camera (hours, %).

| **Child** | **Surface Total (h)** | **Surface IV (h)** | **Surface NIV (h)** | **Surface NIV %** | **Location Total (h)** | **Location IV (h)** | **Location NIV (h)** | **Location NIV %** |
| --- | --- | --- | --- | --- | --- | --- | --- | --- |
| 1 | 2.97 | 2.95 | 0.014 | 0.5 | 2.97 | 2.97 | 0.000 | 0.0 |
| 2 | 2.95 | 1.99 | 0.963 | 32.6 | 3.37 | 2.95 | 0.415 | 12.3 |
| 3 | 2.42 | 2.40 | 0.016 | 0.7 | 2.44 | 2.42 | 0.027 | 1.1 |
| 4 | 3.06 | 2.93 | 0.128 | 4.2 | 3.24 | 3.05 | 0.185 | 5.7 |
| 5 | 0.41 | 0.40 | 0.015 | 3.6 | 0.42 | 0.41 | 0.009 | 2.0 |
| 6 | 2.88 | 2.73 | 0.157 | 5.4 | 2.88 | 2.88 | 0.000 | 0.0 |
| 7 | 1.76 | 1.73 | 0.029 | 1.6 | 1.77 | 1.76 | 0.018 | 1.0 |
| 8 | 1.84 | 1.79 | 0.052 | 2.8 | 1.87 | 1.84 | 0.032 | 1.7 |
| 9 | 2.91 | 2.72 | 0.183 | 6.3 | 2.91 | 2.91 | 0.000 | 0.0 |
| 10 | 2.74 | 2.37 | 0.374 | 13.6 | 2.78 | 2.74 | 0.033 | 1.2 |
| 11 | 2.81 | 2.56 | 0.247 | 8.8 | 2.81 | 2.81 | 0.000 | 0.0 |
| 12 | 2.11 | 2.11 | 0.000 | 0.0 | 2.11 | 2.11 | 0.000 | 0.0 |
| 13 | 2.64 | 2.58 | 0.056 | 2.1 | 2.72 | 2.64 | 0.076 | 2.8 |
| 14 | 1.05 | 0.99 | 0.060 | 5.7 | 1.45 | 1.05 | 0.399 | 27.6 |
| 15 | 2.33 | 2.25 | 0.081 | 3.5 | 2.48 | 2.33 | 0.143 | 5.8 |
| 16 | 2.97 | 2.93 | 0.044 | 1.5 | 3.01 | 2.97 | 0.040 | 1.3 |
| 17 | 2.62 | 2.62 | 0.003 | 0.1 | 2.68 | 2.62 | 0.059 | 2.2 |
| 18 | 2.99 | 2.89 | 0.098 | 3.3 | 3.01 | 2.99 | 0.015 | 0.5 |
| 19 | 1.45 | 1.33 | 0.121 | 8.3 | 1.45 | 1.45 | 0.000 | 0.0 |
| 20 | 3.34 | 3.23 | 0.114 | 3.4 | 3.34 | 3.34 | 0.000 | 0.0 |
| 21 | 3.13 | 3.05 | 0.078 | 2.5 | 3.13 | 3.13 | 0.000 | 0.0 |
| 22 | 2.97 | 2.95 | 0.014 | 0.5 | 2.97 | 2.97 | 0.000 | 0.0 |
| 23 | 2.64 | 2.56 | 0.084 | 3.2 | 3.02 | 2.64 | 0.375 | 12.4 |
| 24 | 3.10 | 3.08 | 0.020 | 0.6 | 3.25 | 3.10 | 0.150 | 4.6 |
| 25 | 2.82 | 2.52 | 0.299 | 10.6 | 2.84 | 2.82 | 0.024 | 0.8 |
| 26 | 1.41 | 1.41 | 0.000 | 0.0 | 1.41 | 1.41 | 0.000 | 0.0 |
| 27 | 2.85 | 2.77 | 0.082 | 2.9 | 2.95 | 2.85 | 0.094 | 3.2 |
| 28 | 3.03 | 2.99 | 0.043 | 1.4 | 3.03 | 3.03 | 0.003 | 0.1 |
| 29 | 3.14 | 1.34 | 1.796 | 57.2 | 3.14 | 3.14 | 0.000 | 0.0 |
| 30 | 3.48 | 3.16 | 0.318 | 9.2 | 3.48 | 3.48 | 0.000 | 0.0 |
| 31 | 3.06 | 3.02 | 0.046 | 1.5 | 3.06 | 3.06 | 0.000 | 0.0 |
| 32 | 2.99 | 2.78 | 0.211 | 7.1 | 2.99 | 2.99 | 0.000 | 0.0 |
| 33 | 3.75 | 3.75 | 0.000 | 0.0 | 3.76 | 3.76 | 0.000 | 0.0 |
| 34 | 2.96 | 2.83 | 0.132 | 4.5 | 2.96 | 2.96 | 0.000 | 0.0 |
| 35 | 2.89 | 2.17 | 0.724 | 25.0 | 2.90 | 2.89 | 0.004 | 0.2 |
| 36 | 3.79 | 2.87 | 0.922 | 24.3 | 3.49 | 3.49 | 0.001 | 0.0 |
| 37 | 3.49 | 3.49 | 0.001 | 0.0 | 3.79 | 3.79 | 0.003 | 0.1 |
| 38 | 1.61 | 1.59 | 0.021 | 1.3 | 1.61 | 1.61 | 0.000 | 0.0 |
| 39 | 2.85 | 2.80 | 0.051 | 1.8 | 2.88 | 2.85 | 0.033 | 1.2 |
| 40 | 3.76 | 3.76 | 0.008 | 0.2 | 3.77 | 3.76 | 0.006 | 0.2 |
| 41 | 1.62 | 1.54 | 0.076 | 4.7 | 1.78 | 1.62 | 0.163 | 9.1 |
| 42 | 3.17 | 2.71 | 0.453 | 14.3 | 3.17 | 3.17 | 0.000 | 0.0 |
| 43 | 2.11 | 1.91 | 0.201 | 9.5 | 2.12 | 2.11 | 0.013 | 0.6 |
| 44 | 1.10 | 1.09 | 0.011 | 1.0 | 1.12 | 1.10 | 0.022 | 1.9 |
| 45 | 1.06 | 1.06 | 0.001 | 0.1 | 1.13 | 1.06 | 0.070 | 6.2 |
| 46 | 0.85 | 0.74 | 0.113 | 13.3 | 0.88 | 0.85 | 0.029 | 3.3 |
| 47 | 2.84 | 2.72 | 0.124 | 4.4 | 2.96 | 2.84 | 0.121 | 4.1 |
| 48 | 3.31 | 3.29 | 0.019 | 0.6 | 3.38 | 3.31 | 0.069 | 2.1 |
| 49 | 3.71 | 3.70 | 0.009 | 0.2 | 3.71 | 3.71 | 0.000 | 0.0 |
| 50 | 1.42 | 1.42 | 0.007 | 0.5 | 1.43 | 1.42 | 0.004 | 0.3 |
| 51 | 3.40 | 3.35 | 0.054 | 1.6 | 3.40 | 3.40 | 0.000 | 0.0 |
| 52 | 3.13 | 2.71 | 0.418 | 13.4 | 3.29 | 3.13 | 0.168 | 5.1 |
| 53 | 2.81 | 1.75 | 1.059 | 37.7 | 2.81 | 2.81 | 0.000 | 0.0 |
| 54 | 3.06 | 2.99 | 0.068 | 2.2 | 3.17 | 3.06 | 0.115 | 3.6 |
| 55 | 3.18 | 3.15 | 0.024 | 0.8 | 3.22 | 3.18 | 0.045 | 1.4 |
| 56 | 2.18 | 2.17 | 0.007 | 0.3 | 2.21 | 2.18 | 0.033 | 1.5 |
| 57 | 3.21 | 3.09 | 0.117 | 3.6 | 3.21 | 3.21 | 0.000 | 0.0 |
| 58 | 3.46 | 3.33 | 0.131 | 3.8 | 3.46 | 3.46 | 0.000 | 0.0 |
| 59 | 3.24 | 3.20 | 0.035 | 1.1 | 3.26 | 3.24 | 0.018 | 0.5 |
| 60 | 3.17 | 3.15 | 0.023 | 0.7 | 3.17 | 3.17 | 0.000 | 0.0 |
| 61 | 2.74 | 2.70 | 0.046 | 1.7 | 2.75 | 2.74 | 0.003 | 0.1 |
| 62 | 3.29 | 3.28 | 0.007 | 0.2 | 3.29 | 3.29 | 0.000 | 0.0 |
| 63 | 2.95 | 2.67 | 0.275 | 9.3 | 2.95 | 2.95 | 0.000 | 0.0 |
| 64 | 2.21 | 1.13 | 1.073 | 48.6 | 2.21 | 2.21 | 0.000 | 0.0 |
| 65 | 3.80 | 2.43 | 1.369 | 36.0 | 3.85 | 3.80 | 0.049 | 1.3 |
| 66 | 3.36 | 3.00 | 0.360 | 10.7 | 3.37 | 3.36 | 0.002 | 0.1 |
| 67 | 3.44 | 3.39 | 0.059 | 1.7 | 3.46 | 3.44 | 0.012 | 0.3 |
| 68 | 2.34 | 2.32 | 0.021 | 0.9 | 2.35 | 2.34 | 0.012 | 0.5 |
| 69 | 2.85 | 2.83 | 0.027 | 1.0 | 2.98 | 2.85 | 0.130 | 4.4 |
| 70 | 3.91 | 3.41 | 0.497 | 12.7 | 3.92 | 3.91 | 0.005 | 0.1 |
| 71 | 2.77 | 2.64 | 0.136 | 4.9 | 2.78 | 2.77 | 0.003 | 0.1 |
| 72 | 2.88 | 2.79 | 0.093 | 3.2 | 2.89 | 2.88 | 0.009 | 0.3 |
| 73 | 4.07 | 4.01 | 0.052 | 1.3 | 4.07 | 4.07 | 0.000 | 0.0 |
| 74 | 2.86 | 2.55 | 0.311 | 10.9 | 2.86 | 2.86 | 0.000 | 0.0 |
| 75 | 1.78 | 1.60 | 0.182 | 10.2 | 2.02 | 1.78 | 0.237 | 11.8 |
| 76 | 2.83 | 2.53 | 0.296 | 10.5 | 2.83 | 2.83 | 0.007 | 0.3 |
| 77 | 3.03 | 2.98 | 0.056 | 1.9 | 3.05 | 3.03 | 0.018 | 0.6 |
| 78 | 3.36 | 3.35 | 0.006 | 0.2 | 3.41 | 3.36 | 0.054 | 1.6 |
| 79 | 3.18 | 3.18 | 0.001 | 0.0 | 3.18 | 3.18 | 0.000 | 0.0 |
| 80 | 3.06 | 2.95 | 0.118 | 3.8 | 3.06 | 3.06 | 0.000 | 0.0 |
| 81 | 2.82 | 2.71 | 0.111 | 3.9 | 2.88 | 2.82 | 0.059 | 2.1 |
| 82 | 2.35 | 2.30 | 0.048 | 2.0 | 2.35 | 2.35 | 0.001 | 0.0 |
| 83 | 2.42 | 2.42 | 0.000 | 0.0 | 2.42 | 2.42 | 0.000 | 0.0 |
| 84 | 1.01 | 0.97 | 0.043 | 4.3 | 1.02 | 1.01 | 0.005 | 0.5 |
| 85 | 2.98 | 2.98 | 0.002 | 0.1 | 2.98 | 2.98 | 0.003 | 0.1 |
| 86 | 3.83 | 3.82 | 0.013 | 0.3 | 3.83 | 3.83 | 0.000 | 0.0 |
| 87 | 3.98 | 3.97 | 0.006 | 0.2 | 4.00 | 3.98 | 0.024 | 0.6 |
| 88 | 2.95 | 2.94 | 0.013 | 0.4 | 2.96 | 2.95 | 0.008 | 0.3 |
| 89 | 3.17 | 3.01 | 0.167 | 5.3 | 3.17 | 3.17 | 0.000 | 0.0 |
| 90 | 3.91 | 3.86 | 0.050 | 1.3 | 3.95 | 3.91 | 0.037 | 0.9 |
| 91 | 4.19 | 4.06 | 0.132 | 3.1 | 4.19 | 4.19 | 0.000 | 0.0 |
| 92 | 3.05 | 3.00 | 0.048 | 1.6 | 3.08 | 3.05 | 0.035 | 1.1 |
| 93 | 0.94 | 0.85 | 0.086 | 9.2 | 0.94 | 0.94 | 0.000 | 0.0 |
| 94 | 1.17 | 1.17 | 0.000 | 0.0 | 1.17 | 1.17 | 0.000 | 0.0 |
| 95 | 3.09 | 3.09 | 0.002 | 0.1 | 3.09 | 3.09 | 0.000 | 0.0 |
| 96 | 3.52 | 1.84 | 1.683 | 47.8 | 4.02 | 3.52 | 0.501 | 12.4 |
| 97 | 2.85 | 2.82 | 0.025 | 0.9 | 2.84 | 2.84 | 0.000 | 0.0 |
| 98 | 2.36 | 2.32 | 0.041 | 1.7 | 2.36 | 2.36 | 0.000 | 0.0 |
| 99 | 1.79 | 1.69 | 0.092 | 5.2 | 1.81 | 1.79 | 0.023 | 1.3 |
| **Total** | **273.10** | **255.00** | **18.10** |  | **277.35** | **273.10** | **4.25** |  |
| **Mean (SD)** | **2.76 (0.80)** | **2.58 (0.80)** | **0.183 (0.337)** | **6.4 (10.8)** | **2.80 (0.80)** | **2.76 (0.80)** | **0.043 (0.092)** | **1.7 (3.8)** |
| **Median (IQR)** | **2.95** | **2.73** | **0.056** | **2.5 (0.7–7.1)** | **2.97** | **2.95** | **0.004** | **0.2 (0.0–1.5)** |
| **Range** | **0.41–4.19** | **0.40–4.06** | **0.000–1.796** | **0.0–57.2** | **0.42–4.19** | **0.41–4.19** | **0.000–0.501** | **0.0–27.6** |

**Note:** Surface Total = Surface IV + Surface NIV. (IV = In-View, NIV (Not-In-View)

Surface IV (255.0 hours total) represents time when the child’s mouth was visible on camera and surface/object coding was possible.

Surface NIV occurred when the mouth was not visible (e.g., child facing away, mouth obscured).

Location Total = Location IV + Location NIV. Location In-View (273.1 hours total) represents time when the child was in a room with camera coverage.

**Supplemental Table S3. Comparison of mouthing behavior findings across studies.**

| **Reference** | **Age Range** | **n** | **Location** | **Method** | **Age Group** | **Mouthing Frequency (contacts/h)** | **Mouthing Duration** | **Notes** |
| --- | --- | --- | --- | --- | --- | --- | --- | --- |
| DIRT (Current Study) | 6 mo – 6 yrs | 99 | NC, AZ, FL, USA | Video (360°), 2.4–2.9 h | 6 to <12 mo | 23.46 (median), 28.62 ± 25.02 (mean ± SD) | 18.67 (median), 16.83 ± 8.55 (mean ± SD) min/h | Total mouthing (all surfaces excl. Nothing) |
|  |  |  |  |  | 1 to <2 yrs | 27.79 (median), 35.76 ± 25.13 (mean ± SD) | 8.74 (median), 11.78 ± 10.65 (mean ± SD) min/h |  |
|  |  |  |  |  | 2 to <3 yrs | 22.53 (median), 26.77 ± 19.43 (mean ± SD) | 8.87 (median), 11.89 ± 12.90 (mean ± SD) min/h |  |
|  |  |  |  |  | 3 to <6 yrs | 21.26 (median), 27.00 ± 22.70 (mean ± SD) | 5.02 (median), 8.66 ± 9.19 (mean ± SD) min/h |  |
| Juberg et al. (2001) | 0–36 mo | 168 | NY, USA | Parent diary, 1–5 d | 0–18 mo | — | 33 (mean), 16 (median) min/day | Duration only; non-pacifier mouthing |
|  |  |  |  |  | 19–36 mo | — | 5 (mean), 0 (median) min/day |  |
| Greene (2002) | 3–81 mo | 169 | TX, IL, USA | Trained observers, 2 h × 2 d | <12 mo | — | 10.5 (mean) min/h | Duration only |
|  |  |  |  |  | 1 to <2 yrs | — | 8.7 (mean) min/h |  |
|  |  |  |  |  | 2 to <3 yrs | — | 5.3 (mean) min/h |  |
| Beamer et al. (2008) | 6–27 mo | 23 | CA, USA | Video, 4 h | 6–13 mo | OtM: 37.8 (mean), 35.2 (median); HtM: 18.4 (mean), 15.2 (median) | OtM: 4.5 (mean), 2.8 (median) min/h | Object-to-mouth (OtM) and hand-to-mouth (HtM) reported separately |
|  |  |  |  |  | 20–27 mo | OtM: 18.0 (mean), 15.9 (median) | OtM: 2.1 (mean), 1.3 (median) min/h |  |
|  |  |  |  |  | Overall | OtM: 29.2 (mean), 27.2 (median); HtM: 18.4 (mean), 15.2 (median) | OtM: 2.2 min/h; HtM: 1.2 min/h (hourly) |  |
| Zartarian et al. (1998) | 2–4 yrs | 4 | CA, USA | Video, 6–10 h | 2–4 yrs | 11 (mean), 9 (median) | ~2.6 (mean) min/h | 4.35% of observation time |
| Groot et al. (1998) | 3–36 mo | 42 | Netherlands | Parent + stopwatch, 2.5 h/d | 3–6 mo | — | 36.9 (mean) min/day | Duration only; excludes pacifier |
|  |  |  |  |  | 6–12 mo | — | 44.0 (mean) min/day |  |
|  |  |  |  |  | 12–18 mo | — | 16.4 (mean) min/day |  |
|  |  |  |  |  | 18–36 mo | — | 9.3 (mean) min/day |  |
| Black et al. (2005) | 7–53 mo | 52 | TX, USA | Video, 4 h | 7–12 mo | 32.1 (median, HtM+OtM) | — | Sum of median HtM + median OtM frequencies |
|  |  |  |  |  | 13–24 mo | 21.7 (median, HtM+OtM) | — |  |
|  |  |  |  |  | 25–36 mo | 15.4 (median, HtM+OtM) | — |  |
|  |  |  |  |  | 37–53 mo | 27.8 (median, HtM+OtM) | — |  |
| Freeman et al. (2001) | 3–13 yrs | 19 | MN, USA | Video, 4 h | 3–4 yrs | HtM: 3.5 (median), 4 (mean); OtM: 3 (median), 6 (mean) | — | Hand-to-mouth (HtM) and object-to-mouth (OtM) reported separately |
|  |  |  |  |  | 5–6 yrs | HtM: 2.5 (median), 8 (mean); OtM: 0 (median), 1 (mean) | — |  |
|  |  |  |  |  | 7–8 yrs | HtM: 3 (median), 5 (mean); OtM: 0 (median), 1 (mean) | — |  |
|  |  |  |  |  | 10–12 yrs | HtM: 2 (median), 4 (mean); OtM: 0 (median), 1 (mean) | — |  |
| Reed et al. (1999) | 2–6 yrs | 30 | NJ, USA | Video, ~1 day | 2–6 yrs | 9.5 (mean), 8.5 (median) | — | Hand-to-mouth only |
| Tulve et al. (2002) | 11–60 mo | 72 | WA, USA | Trained observers, 15-min periods | ≤24 mo | 81 (mean), 73 (median) | — | Total mouthing (hand + body + surface + toy) |
|  |  |  |  |  | >24 mo | 42 (mean), 31 (median) | — |  |
| Kwong et al. (2016) | 3–18 mo | 148 | Bangladesh | Structured observation, 5 h | 3–6 mo | 37.3 (median) | — | Hand-mouthing only |
|  |  |  |  |  | 6–12 mo | 34.4 (median) | — |  |
|  |  |  |  |  | 12–18 mo | 29.7 (median) | — |  |

*Values are reported as median or mean ± SD as specified. Dashes (—) indicate the metric was not reported. HtM = hand-to-mouth; OtM = object-to-mouth.*

*NC = North Carolina, AZ = Arizona, FL = Florida, CA = California, NY = New York, TX = Texas, IL = Illinois, MN = Minnesota, NJ = New Jersey, WA = Washington.*

**Supplemental Table S4. Overall distribution of mouthing frequency (objects/surfaces).**

**All observations correspond to 99 subjects**

| **Object/Surface** | **Total_Contacts** | **Mean** | **SD** | **Min** | **P5** | **P25** | **P50** | **P75** | **P95** | **Max** |
| --- | --- | --- | --- | --- | --- | --- | --- | --- | --- | --- |
| **Nothing** | 11002 | 47.57 | 29.76 | 3.23 | 12.62 | 27.92 | 41.63 | 56.57 | 101.84 | 168.44 |
| **Other_Food** | 1850 | 7.52 | 7.95 | 0 | 0 | 1.29 | 5.23 | 10.83 | 22.74 | 39.97 |
| **Hands** | 1794 | 7.23 | 7.99 | 0 | 0 | 1.74 | 4.78 | 9.82 | 23.89 | 43.02 |
| **Food-Container** | 1580 | 6.14 | 7.2 | 0 | 0 | 0.86 | 3.82 | 9.26 | 23.03 | 30.47 |
| **Sticky_Food** | 341 | 1.68 | 5.67 | 0 | 0 | 0 | 0 | 0.35 | 9.29 | 43.76 |
| **Porous-Plastic-Toy** | 334 | 1.32 | 4.13 | 0 | 0 | 0 | 0 | 0.89 | 5.07 | 34.52 |
| **Beverage** | 263 | 1.11 | 3.42 | 0 | 0 | 0 | 0 | 0.13 | 6.59 | 26.66 |
| **Pacifier** | 240 | 1.07 | 4.54 | 0 | 0 | 0 | 0 | 0 | 3.71 | 33.46 |
| **Bedding/Towels** | 193 | 0.94 | 3.98 | 0 | 0 | 0 | 0 | 0 | 3.19 | 28.51 |
| **Hair/Body** | 175 | 0.63 | 1.41 | 0 | 0 | 0 | 0 | 0.38 | 3.69 | 7.37 |
| **Paper/Wrapper** | 127 | 0.5 | 1.53 | 0 | 0 | 0 | 0 | 0.13 | 2.37 | 8.99 |
| **Clothes** | 91 | 0.38 | 0.99 | 0 | 0 | 0 | 0 | 0.34 | 2.34 | 7.75 |
| **Fabric_Wall/Furniture** | 52 | 0.2 | 0.61 | 0 | 0 | 0 | 0 | 0 | 0.93 | 4.68 |
| **Hard_Toy** | 48 | 0.18 | 0.66 | 0 | 0 | 0 | 0 | 0 | 1.65 | 3.41 |
| **Plastic-Tool/Appliance** | 42 | 0.16 | 0.75 | 0 | 0 | 0 | 0 | 0 | 0.9 | 6.6 |
| **Fabric_Toy** | 37 | 0.14 | 0.41 | 0 | 0 | 0 | 0 | 0 | 1.18 | 2.23 |
| **Electronics** | 13 | 0.05 | 0.2 | 0 | 0 | 0 | 0 | 0 | 0.43 | 1.15 |
| **Water** | 9 | 0.05 | 0.3 | 0 | 0 | 0 | 0 | 0 | 0.03 | 2.83 |
| **Metal_Wall/Furniture** | 9 | 0.03 | 0.19 | 0 | 0 | 0 | 0 | 0 | 0 | 1.76 |
| **Footwear** | 5 | 0.02 | 0.15 | 0 | 0 | 0 | 0 | 0 | 0 | 1.47 |
| **Animal** | 3 | 0.01 | 0.08 | 0 | 0 | 0 | 0 | 0 | 0 | 0.58 |
| **Carpet/Mat** | 1 | 0.01 | 0.07 | 0 | 0 | 0 | 0 | 0 | 0 | 0.71 |
| **Metal_Tool/Appliance** | 1 | 0.01 | 0.07 | 0 | 0 | 0 | 0 | 0 | 0 | 0.74 |
| **Nose** | 2 | 0.01 | 0.05 | 0 | 0 | 0 | 0 | 0 | 0 | 0.37 |
| **Plastic_Wall/Furniture** | 2 | 0.01 | 0.05 | 0 | 0 | 0 | 0 | 0 | 0 | 0.38 |
| **Wood_Wall/Furniture** | 3 | 0.01 | 0.11 | 0 | 0 | 0 | 0 | 0 | 0 | 1.05 |
| **Wood_Toy** | 1 | 0 | 0.04 | 0 | 0 | 0 | 0 | 0 | 0 | 0.35 |

*Values in contacts/h. Sorted by mean frequency (descending). Observation time = 255.00 hours (Surface Not-In-View excluded).*

**Supplemental Table S5. Distribution of indoor object/surface hourly mouthing duration and frequency by Ethnicity.**

| **Group** | **Object/Surface** | **Med Dur** | **Q25 Dur** | **Q75 Dur** | **Mean Dur** | **SD Dur** | **Med Freq** | **Q25 Freq** | **Q75 Freq** | **Mean Freq** | **SD Freq** |
| --- | --- | --- | --- | --- | --- | --- | --- | --- | --- | --- | --- |
| ***Hispanic (n=39)*** | Animal | 0 | 0 | 0 | 0 | 0 | 0 | 0 | 0 | 0.01 | 0.07 |
|  | Bedding/Towels | 0 | 0 | 0 | 0.03 | 0.12 | 0 | 0 | 0 | 0.42 | 1.25 |
|  | Beverage | 0 | 0 | 0 | 0.2 | 0.69 | 0 | 0 | 0 | 0.73 | 2 |
|  | Carpet/Mat | 0 | 0 | 0 | 0 | 0 | 0 | 0 | 0 | 0 | 0 |
|  | Clothes | 0 | 0 | 0 | 0.07 | 0.4 | 0 | 0 | 0.34 | 0.25 | 0.49 |
|  | Electronics | 0 | 0 | 0 | 0.02 | 0.1 | 0 | 0 | 0 | 0.06 | 0.21 |
|  | Fabric_Toy | 0 | 0 | 0 | 0.05 | 0.15 | 0 | 0 | 0 | 0.19 | 0.5 |
|  | Fabric_Wall/Furniture | 0 | 0 | 0 | 0.01 | 0.03 | 0 | 0 | 0 | 0.21 | 0.45 |
|  | Food-Container | 0.78 | 0.11 | 2.24 | 2.47 | 4.15 | 4.21 | 0.92 | 9.46 | 6.4 | 7.61 |
|  | Footwear | 0 | 0 | 0 | 0 | 0 | 0 | 0 | 0 | 0 | 0 |
|  | Hair/Body | 0 | 0 | 0 | 1.25 | 4.58 | 0 | 0 | 0.25 | 0.61 | 1.56 |
|  | Hands | 0.66 | 0.06 | 2 | 1.33 | 1.77 | 3.78 | 1.47 | 8.54 | 5.83 | 6.14 |
|  | Hard_Toy | 0 | 0 | 0 | 0 | 0.02 | 0 | 0 | 0 | 0.11 | 0.52 |
|  | Metal_Tool/Appliance | 0 | 0 | 0 | 0 | 0 | 0 | 0 | 0 | 0.02 | 0.12 |
|  | Metal_Wall/Furniture | 0 | 0 | 0 | 0 | 0 | 0 | 0 | 0 | 0.01 | 0.05 |
|  | Nose | 0 | 0 | 0 | 0 | 0 | 0 | 0 | 0 | 0 | 0 |
|  | Nothing | 51.25 | 40.87 | 55.49 | 47.12 | 12.39 | 37.62 | 26.39 | 55.65 | 43.62 | 26.52 |
|  | Other_Food | 2.17 | 0.32 | 4.49 | 2.94 | 3.46 | 6.03 | 1.29 | 11.97 | 8.08 | 7.76 |
|  | Pacifier | 0 | 0 | 0 | 3.49 | 11.11 | 0 | 0 | 0 | 1.42 | 5.75 |
|  | Paper/Wrapper | 0 | 0 | 0 | 0.02 | 0.05 | 0 | 0 | 0 | 0.17 | 0.47 |
|  | Plastic_Wall/Furniture | 0 | 0 | 0 | 0 | 0.01 | 0 | 0 | 0 | 0.01 | 0.05 |
|  | Plastic-Tool/Appliance | 0 | 0 | 0 | 0.05 | 0.27 | 0 | 0 | 0 | 0.15 | 0.55 |
|  | Porous-Plastic-Toy | 0 | 0 | 0.23 | 0.46 | 1.65 | 0 | 0 | 1.3 | 1.92 | 6 |
|  | Sticky_Food | 0 | 0 | 0 | 0.46 | 2.07 | 0 | 0 | 0 | 1.5 | 7.02 |
|  | Water | 0 | 0 | 0 | 0 | 0.02 | 0 | 0 | 0 | 0.1 | 0.47 |
|  | Wood_Toy | 0 | 0 | 0 | 0 | 0 | 0 | 0 | 0 | 0 | 0 |
|  | Wood_Wall/Furniture | 0 | 0 | 0 | 0 | 0 | 0 | 0 | 0 | 0 | 0 |
|  | **Dietary Total** | 2.36 | 0.32 | 4.49 | 3.6 | 4.82 | 7.99 | 2.05 | 16.28 | 10.33 | 11.74 |
|  | **Non-Dietary Total** | 5.43 | 1.52 | 11.93 | 9.28 | 12.18 | 13.05 | 7.17 | 20.63 | 17.94 | 17.07 |
|  | **Total Active Mouthing** | 8.74 | 4.51 | 19.13 | 12.88 | 12.39 | 23.6 | 12.6 | 35.53 | 28.27 | 25.08 |
| ***Non-Hispanic (n=60)*** | Animal | 0 | 0 | 0 | 0 | 0.01 | 0 | 0 | 0 | 0.02 | 0.09 |
|  | Bedding/Towels | 0 | 0 | 0 | 0.5 | 2.14 | 0 | 0 | 0 | 1.28 | 5 |
|  | Beverage | 0 | 0 | 0.07 | 0.16 | 0.37 | 0 | 0 | 0.8 | 1.36 | 4.08 |
|  | Carpet/Mat | 0 | 0 | 0 | 0 | 0.02 | 0 | 0 | 0 | 0.01 | 0.09 |
|  | Clothes | 0 | 0 | 0.01 | 0.18 | 1.17 | 0 | 0 | 0.34 | 0.46 | 1.21 |
|  | Electronics | 0 | 0 | 0 | 0.01 | 0.05 | 0 | 0 | 0 | 0.04 | 0.19 |
|  | Fabric_Toy | 0 | 0 | 0 | 0.01 | 0.07 | 0 | 0 | 0 | 0.12 | 0.35 |
|  | Fabric_Wall/Furniture | 0 | 0 | 0 | 0.02 | 0.09 | 0 | 0 | 0 | 0.18 | 0.69 |
|  | Food-Container | 0.73 | 0.07 | 1.95 | 1.77 | 3.21 | 3.81 | 0.76 | 8.97 | 5.97 | 6.98 |
|  | Footwear | 0 | 0 | 0 | 0 | 0 | 0 | 0 | 0 | 0.03 | 0.19 |
|  | Hair/Body | 0 | 0 | 0.02 | 0.78 | 2.81 | 0 | 0 | 0.47 | 0.64 | 1.32 |
|  | Hands | 0.64 | 0.13 | 2.02 | 2.03 | 3.67 | 5.1 | 1.93 | 11.25 | 8.13 | 8.93 |
|  | Hard_Toy | 0 | 0 | 0 | 0.03 | 0.09 | 0 | 0 | 0 | 0.23 | 0.74 |
|  | Metal_Tool/Appliance | 0 | 0 | 0 | 0 | 0 | 0 | 0 | 0 | 0 | 0 |
|  | Metal_Wall/Furniture | 0 | 0 | 0 | 0 | 0.02 | 0 | 0 | 0 | 0.04 | 0.24 |
|  | Nose | 0 | 0 | 0 | 0 | 0 | 0 | 0 | 0 | 0.01 | 0.06 |
|  | Nothing | 51.59 | 46.63 | 55.56 | 49.25 | 9.31 | 43.67 | 32.44 | 56.55 | 50.14 | 31.64 |
|  | Other_Food | 1.64 | 0.46 | 3.38 | 2.5 | 2.81 | 4.9 | 1.51 | 8.83 | 7.16 | 8.12 |
|  | Pacifier | 0 | 0 | 0 | 1.72 | 6.04 | 0 | 0 | 0 | 0.83 | 3.58 |
|  | Paper/Wrapper | 0 | 0 | 0.01 | 0.12 | 0.51 | 0 | 0 | 0.31 | 0.72 | 1.9 |
|  | Plastic_Wall/Furniture | 0 | 0 | 0 | 0 | 0.01 | 0 | 0 | 0 | 0.01 | 0.05 |
|  | Plastic-Tool/Appliance | 0 | 0 | 0 | 0.02 | 0.12 | 0 | 0 | 0 | 0.16 | 0.87 |
|  | Porous-Plastic-Toy | 0 | 0 | 0.08 | 0.2 | 0.59 | 0 | 0 | 0.69 | 0.93 | 2.16 |
|  | Sticky_Food | 0 | 0 | 0.39 | 0.68 | 2.08 | 0 | 0 | 0.93 | 1.79 | 4.66 |
|  | Water | 0 | 0 | 0 | 0 | 0 | 0 | 0 | 0 | 0.02 | 0.1 |
|  | Wood_Toy | 0 | 0 | 0 | 0 | 0.01 | 0 | 0 | 0 | 0.01 | 0.04 |
|  | Wood_Wall/Furniture | 0 | 0 | 0 | 0.01 | 0.08 | 0 | 0 | 0 | 0.02 | 0.13 |
|  | **Dietary Total** | 2.89 | 0.86 | 4.1 | 3.32 | 3.22 | 7.88 | 3.31 | 14.25 | 10.3 | 9.6 |
|  | **Non-Dietary Total** | 3.68 | 1.15 | 10.56 | 7.5 | 8.95 | 14.81 | 8.95 | 26.61 | 19.78 | 15.98 |
|  | **Total Active Mouthing** | 8.41 | 4.44 | 13.37 | 10.83 | 9.43 | 23.53 | 15.08 | 43.09 | 30.09 | 21.65 |

*Duration in min/h; Frequency in contacts/h. Observation time = 255.00 hours (Surface Not-In-View excluded).*

**Supplemental Table S6. Distribution of indoor object/surface hourly mouthing duration and frequency by Race.**

| **Group** | **Object/Surface** | **Med Dur** | **Q25 Dur** | **Q75 Dur** | **Mean Dur** | **SD Dur** | **Med Freq** | **Q25 Freq** | **Q75 Freq** | **Mean Freq** | **SD Freq** |
| --- | --- | --- | --- | --- | --- | --- | --- | --- | --- | --- | --- |
| ***Asian (n=6)*** | Animal | 0 | 0 | 0 | 0 | 0 | 0 | 0 | 0 | 0 | 0 |
|  | Bedding/Towels | 0 | 0 | 0 | 0.01 | 0.02 | 0 | 0 | 0 | 0.11 | 0.28 |
|  | Beverage | 0 | 0 | 0.01 | 0.09 | 0.21 | 0 | 0 | 0.76 | 0.7 | 1.28 |
|  | Carpet/Mat | 0 | 0 | 0 | 0 | 0 | 0 | 0 | 0 | 0 | 0 |
|  | Clothes | 0 | 0 | 0 | 0 | 0 | 0 | 0 | 0 | 0.05 | 0.12 |
|  | Electronics | 0 | 0 | 0 | 0 | 0 | 0 | 0 | 0 | 0 | 0 |
|  | Fabric_Toy | 0 | 0 | 0 | 0 | 0 | 0 | 0 | 0 | 0 | 0 |
|  | Fabric_Wall/Furniture | 0 | 0 | 0 | 0 | 0.01 | 0 | 0 | 0 | 0.05 | 0.13 |
|  | Food-Container | 0.79 | 0.45 | 1.04 | 1.33 | 1.8 | 11.04 | 3.19 | 18.34 | 12.37 | 11.69 |
|  | Footwear | 0 | 0 | 0 | 0 | 0 | 0 | 0 | 0 | 0 | 0 |
|  | Hair/Body | 0 | 0 | 0 | 0.01 | 0.01 | 0 | 0 | 0.45 | 0.36 | 0.64 |
|  | Hands | 0.54 | 0.33 | 1.11 | 0.76 | 0.67 | 4.73 | 4.46 | 10.12 | 7.91 | 5.92 |
|  | Hard_Toy | 0 | 0 | 0 | 0 | 0 | 0 | 0 | 0 | 0 | 0 |
|  | Metal_Tool/Appliance | 0 | 0 | 0 | 0 | 0 | 0 | 0 | 0 | 0 | 0 |
|  | Metal_Wall/Furniture | 0 | 0 | 0 | 0 | 0 | 0 | 0 | 0 | 0 | 0 |
|  | Nose | 0 | 0 | 0 | 0 | 0 | 0 | 0 | 0 | 0 | 0 |
|  | Nothing | 54.86 | 49.76 | 55.39 | 52.61 | 4.61 | 56.3 | 43.77 | 56.52 | 52.44 | 18.29 |
|  | Other_Food | 3.6 | 3.06 | 6.53 | 4.37 | 2.62 | 13.43 | 7.74 | 18.3 | 13.4 | 7.01 |
|  | Pacifier | 0 | 0 | 0 | 0 | 0 | 0 | 0 | 0 | 0 | 0 |
|  | Paper/Wrapper | 0.03 | 0.01 | 0.03 | 0.6 | 1.43 | 0.95 | 0.18 | 2.17 | 2.21 | 3.4 |
|  | Plastic_Wall/Furniture | 0 | 0 | 0 | 0 | 0 | 0 | 0 | 0 | 0 | 0 |
|  | Plastic-Tool/Appliance | 0 | 0 | 0 | 0 | 0 | 0 | 0 | 0 | 0 | 0 |
|  | Porous-Plastic-Toy | 0 | 0 | 0 | 0 | 0 | 0 | 0 | 0 | 0 | 0 |
|  | Sticky_Food | 0 | 0 | 0.35 | 0.21 | 0.34 | 0.15 | 0 | 1.26 | 0.96 | 1.56 |
|  | Water | 0 | 0 | 0 | 0 | 0 | 0 | 0 | 0 | 0 | 0 |
|  | Wood_Toy | 0 | 0 | 0 | 0 | 0 | 0 | 0 | 0 | 0 | 0 |
|  | Wood_Wall/Furniture | 0 | 0 | 0 | 0 | 0 | 0 | 0 | 0 | 0 | 0 |
|  | **Dietary Total** | 3.6 | 3.06 | 6.69 | 4.67 | 2.45 | 14.38 | 12.25 | 18.37 | 15.06 | 5.95 |
|  | **Non-Dietary Total** | 1.74 | 1.13 | 3.58 | 2.72 | 2.32 | 22.39 | 16.02 | 28.65 | 23.07 | 12.89 |
|  | **Total Active Mouthing** | 5.14 | 4.61 | 10.23 | 7.39 | 4.61 | 35.97 | 23.97 | 47.03 | 38.13 | 18.02 |
| ***Black or African American (n=25)*** | Animal | 0 | 0 | 0 | 0 | 0.01 | 0 | 0 | 0 | 0.01 | 0.07 |
|  | Bedding/Towels | 0 | 0 | 0 | 1 | 3.16 | 0 | 0 | 0 | 2.49 | 7.43 |
|  | Beverage | 0 | 0 | 0 | 0.19 | 0.62 | 0 | 0 | 0 | 0.37 | 1.07 |
|  | Carpet/Mat | 0 | 0 | 0 | 0 | 0 | 0 | 0 | 0 | 0 | 0 |
|  | Clothes | 0 | 0 | 0.03 | 0.41 | 1.8 | 0 | 0 | 0.73 | 0.85 | 1.68 |
|  | Electronics | 0 | 0 | 0 | 0.02 | 0.07 | 0 | 0 | 0 | 0.12 | 0.31 |
|  | Fabric_Toy | 0 | 0 | 0 | 0 | 0.01 | 0 | 0 | 0 | 0.03 | 0.14 |
|  | Fabric_Wall/Furniture | 0 | 0 | 0 | 0.04 | 0.11 | 0 | 0 | 0 | 0.18 | 0.53 |
|  | Food-Container | 0.77 | 0.15 | 1.73 | 1.47 | 2.6 | 3.61 | 1.55 | 7.54 | 5.4 | 6.05 |
|  | Footwear | 0 | 0 | 0 | 0 | 0 | 0 | 0 | 0 | 0.07 | 0.3 |
|  | Hair/Body | 0 | 0 | 0.03 | 0.72 | 3.29 | 0 | 0 | 0.42 | 0.65 | 1.27 |
|  | Hands | 1.46 | 0.24 | 2.88 | 2.78 | 4.21 | 6.24 | 3.69 | 18 | 11.4 | 11.43 |
|  | Hard_Toy | 0 | 0 | 0 | 0.02 | 0.1 | 0 | 0 | 0 | 0.09 | 0.35 |
|  | Metal_Tool/Appliance | 0 | 0 | 0 | 0 | 0 | 0 | 0 | 0 | 0 | 0 |
|  | Metal_Wall/Furniture | 0 | 0 | 0 | 0 | 0 | 0 | 0 | 0 | 0 | 0 |
|  | Nose | 0 | 0 | 0 | 0 | 0 | 0 | 0 | 0 | 0.03 | 0.1 |
|  | Nothing | 50.34 | 45.15 | 56.39 | 48.69 | 9.95 | 42.21 | 33.53 | 54.01 | 50.35 | 34.44 |
|  | Other_Food | 0.9 | 0 | 2.47 | 2.33 | 3.72 | 4.5 | 0 | 8.67 | 6.97 | 9.4 |
|  | Pacifier | 0 | 0 | 0 | 1.09 | 3.93 | 0 | 0 | 0 | 0.42 | 1.66 |
|  | Paper/Wrapper | 0 | 0 | 0 | 0.11 | 0.36 | 0 | 0 | 0 | 0.85 | 2.27 |
|  | Plastic_Wall/Furniture | 0 | 0 | 0 | 0 | 0 | 0 | 0 | 0 | 0 | 0 |
|  | Plastic-Tool/Appliance | 0 | 0 | 0 | 0.04 | 0.18 | 0 | 0 | 0 | 0.31 | 1.32 |
|  | Porous-Plastic-Toy | 0.01 | 0 | 0.24 | 0.39 | 0.87 | 0.33 | 0 | 0.73 | 1.59 | 3.06 |
|  | Sticky_Food | 0 | 0 | 0 | 0.65 | 2.48 | 0 | 0 | 0 | 0.74 | 2.03 |
|  | Water | 0 | 0 | 0 | 0 | 0 | 0 | 0 | 0 | 0 | 0 |
|  | Wood_Toy | 0 | 0 | 0 | 0 | 0.01 | 0 | 0 | 0 | 0.01 | 0.07 |
|  | Wood_Wall/Furniture | 0 | 0 | 0 | 0.03 | 0.13 | 0 | 0 | 0 | 0.04 | 0.21 |
|  | **Dietary Total** | 1.74 | 0.09 | 3.23 | 3.15 | 4.46 | 5.14 | 0.99 | 8.98 | 8.02 | 9.4 |
|  | **Non-Dietary Total** | 4.08 | 2.16 | 12.48 | 8.35 | 8.17 | 16.24 | 9.28 | 38.28 | 24.33 | 20.13 |
|  | **Total Active Mouthing** | 9.66 | 3.61 | 14.85 | 11.5 | 10.2 | 25.78 | 13.21 | 51.32 | 32.35 | 26.88 |
| ***Mixed (n=15)*** | Animal | 0 | 0 | 0 | 0 | 0 | 0 | 0 | 0 | 0.04 | 0.15 |
|  | Bedding/Towels | 0 | 0 | 0.01 | 0.35 | 1.2 | 0 | 0 | 0.31 | 0.83 | 2.37 |
|  | Beverage | 0 | 0 | 0.17 | 0.22 | 0.46 | 0 | 0 | 1.1 | 1.5 | 3.05 |
|  | Carpet/Mat | 0 | 0 | 0 | 0.01 | 0.04 | 0 | 0 | 0 | 0.05 | 0.18 |
|  | Clothes | 0 | 0 | 0 | 0.18 | 0.65 | 0 | 0 | 0 | 0.31 | 0.72 |
|  | Electronics | 0 | 0 | 0 | 0 | 0 | 0 | 0 | 0 | 0 | 0 |
|  | Fabric_Toy | 0 | 0 | 0 | 0.05 | 0.16 | 0 | 0 | 0 | 0.15 | 0.4 |
|  | Fabric_Wall/Furniture | 0 | 0 | 0 | 0.02 | 0.04 | 0 | 0 | 0 | 0.16 | 0.37 |
|  | Food-Container | 0.31 | 0 | 1.42 | 1.49 | 3.05 | 2.21 | 0 | 4.99 | 3.85 | 4.78 |
|  | Footwear | 0 | 0 | 0 | 0 | 0 | 0 | 0 | 0 | 0 | 0 |
|  | Hair/Body | 0 | 0 | 0 | 0.68 | 2.26 | 0 | 0 | 0.15 | 0.52 | 1.48 |
|  | Hands | 0.16 | 0.05 | 0.77 | 1.57 | 4.45 | 2.27 | 1.12 | 7.82 | 5.52 | 7 |
|  | Hard_Toy | 0 | 0 | 0 | 0.02 | 0.08 | 0 | 0 | 0 | 0.12 | 0.48 |
|  | Metal_Tool/Appliance | 0 | 0 | 0 | 0 | 0 | 0 | 0 | 0 | 0 | 0 |
|  | Metal_Wall/Furniture | 0 | 0 | 0 | 0 | 0.01 | 0 | 0 | 0 | 0.05 | 0.19 |
|  | Nose | 0 | 0 | 0 | 0 | 0 | 0 | 0 | 0 | 0 | 0 |
|  | Nothing | 50.93 | 44.78 | 55.57 | 48.88 | 9.55 | 39.45 | 25.81 | 57.25 | 45.79 | 36.32 |
|  | Other_Food | 1.15 | 0.17 | 3.9 | 2.55 | 3.23 | 3.57 | 0.27 | 7.85 | 5.63 | 7.48 |
|  | Pacifier | 0 | 0 | 0 | 3.41 | 7.15 | 0 | 0 | 0 | 0.86 | 2.37 |
|  | Paper/Wrapper | 0 | 0 | 0.01 | 0.02 | 0.05 | 0 | 0 | 0.16 | 0.21 | 0.52 |
|  | Plastic_Wall/Furniture | 0 | 0 | 0 | 0 | 0 | 0 | 0 | 0 | 0 | 0 |
|  | Plastic-Tool/Appliance | 0 | 0 | 0 | 0 | 0 | 0 | 0 | 0 | 0 | 0 |
|  | Porous-Plastic-Toy | 0 | 0 | 0.1 | 0.1 | 0.2 | 0 | 0 | 1.3 | 0.85 | 1.52 |
|  | Sticky_Food | 0 | 0 | 0.81 | 0.46 | 0.68 | 0 | 0 | 2.78 | 1.97 | 3.43 |
|  | Water | 0 | 0 | 0 | 0 | 0 | 0 | 0 | 0 | 0 | 0 |
|  | Wood_Toy | 0 | 0 | 0 | 0 | 0 | 0 | 0 | 0 | 0 | 0 |
|  | Wood_Wall/Furniture | 0 | 0 | 0 | 0 | 0 | 0 | 0 | 0 | 0 | 0 |
|  | **Dietary Total** | 2.21 | 0.78 | 4.42 | 3.22 | 3.2 | 8.67 | 3.54 | 13.65 | 9.16 | 7.86 |
|  | **Non-Dietary Total** | 2.44 | 0.5 | 13.4 | 7.9 | 10.56 | 12.15 | 7.17 | 18.54 | 13.59 | 10.48 |
|  | **Total Active Mouthing** | 9.03 | 4.43 | 15.22 | 11.12 | 9.54 | 21.26 | 14 | 25.74 | 22.75 | 15.49 |
| ***Other (n=3)*** | Animal | 0 | 0 | 0 | 0 | 0 | 0 | 0 | 0 | 0 | 0 |
|  | Bedding/Towels | 0 | 0 | 0.02 | 0.01 | 0.02 | 0 | 0 | 0.75 | 0.5 | 0.86 |
|  | Beverage | 0 | 0 | 0.23 | 0.16 | 0.27 | 0 | 0 | 1.35 | 0.9 | 1.55 |
|  | Carpet/Mat | 0 | 0 | 0 | 0 | 0 | 0 | 0 | 0 | 0 | 0 |
|  | Clothes | 0 | 0 | 0 | 0 | 0 | 0 | 0 | 0 | 0 | 0 |
|  | Electronics | 0 | 0 | 0 | 0 | 0 | 0 | 0 | 0 | 0 | 0 |
|  | Fabric_Toy | 0 | 0 | 0 | 0 | 0.01 | 0 | 0 | 0.26 | 0.17 | 0.3 |
|  | Fabric_Wall/Furniture | 0 | 0 | 0 | 0 | 0.01 | 0 | 0 | 0.45 | 0.3 | 0.52 |
|  | Food-Container | 1.16 | 0.58 | 1.65 | 1.1 | 1.07 | 5.24 | 2.62 | 8.64 | 5.76 | 6.04 |
|  | Footwear | 0 | 0 | 0 | 0 | 0 | 0 | 0 | 0 | 0 | 0 |
|  | Hair/Body | 0 | 0 | 1.02 | 0.68 | 1.18 | 0 | 0 | 2.62 | 1.74 | 3.02 |
|  | Hands | 0.76 | 0.38 | 0.86 | 0.57 | 0.51 | 5.24 | 2.77 | 8.9 | 6.03 | 6.17 |
|  | Hard_Toy | 0 | 0 | 0.02 | 0.02 | 0.03 | 0 | 0 | 0.3 | 0.2 | 0.35 |
|  | Metal_Tool/Appliance | 0 | 0 | 0 | 0 | 0 | 0 | 0 | 0 | 0 | 0 |
|  | Metal_Wall/Furniture | 0 | 0 | 0 | 0 | 0.01 | 0 | 0 | 0.15 | 0.1 | 0.17 |
|  | Nose | 0 | 0 | 0 | 0 | 0 | 0 | 0 | 0 | 0 | 0 |
|  | Nothing | 55.58 | 53.42 | 56.04 | 54.44 | 2.8 | 37.66 | 32.86 | 74.58 | 59.07 | 45.66 |
|  | Other_Food | 3.32 | 1.85 | 3.52 | 2.47 | 1.82 | 4.78 | 4.07 | 11.55 | 8.82 | 8.26 |
|  | Pacifier | 0 | 0 | 0 | 0 | 0 | 0 | 0 | 0 | 0 | 0 |
|  | Paper/Wrapper | 0 | 0 | 0.04 | 0.03 | 0.05 | 0 | 0 | 1.05 | 0.7 | 1.21 |
|  | Plastic_Wall/Furniture | 0 | 0 | 0 | 0 | 0 | 0 | 0 | 0 | 0 | 0 |
|  | Plastic-Tool/Appliance | 0 | 0 | 0 | 0 | 0 | 0 | 0 | 0 | 0 | 0 |
|  | Porous-Plastic-Toy | 0.01 | 0 | 0.74 | 0.49 | 0.84 | 0.37 | 0.19 | 8.3 | 5.53 | 9.26 |
|  | Sticky_Food | 0.02 | 0.01 | 0.03 | 0.02 | 0.02 | 0.37 | 0.19 | 0.49 | 0.32 | 0.3 |
|  | Water | 0 | 0 | 0 | 0 | 0 | 0 | 0 | 0 | 0 | 0 |
|  | Wood_Toy | 0 | 0 | 0 | 0 | 0 | 0 | 0 | 0 | 0 | 0 |
|  | Wood_Wall/Furniture | 0 | 0 | 0 | 0 | 0 | 0 | 0 | 0 | 0 | 0 |
|  | **Dietary Total** | 3.31 | 1.86 | 3.77 | 2.65 | 2 | 8.07 | 5.91 | 13.19 | 10.04 | 7.48 |
|  | **Non-Dietary Total** | 3.1 | 1.64 | 4.27 | 2.91 | 2.63 | 10.85 | 8.26 | 28.7 | 21.03 | 22.26 |
|  | **Total Active Mouthing** | 4.42 | 3.96 | 6.58 | 5.56 | 2.8 | 14.59 | 14.17 | 39.73 | 31.07 | 29.27 |
| ***White (n=50)*** | Animal | 0 | 0 | 0 | 0 | 0 | 0 | 0 | 0 | 0.01 | 0.07 |
|  | Bedding/Towels | 0 | 0 | 0 | 0.03 | 0.09 | 0 | 0 | 0 | 0.33 | 1.11 |
|  | Beverage | 0 | 0 | 0 | 0.16 | 0.52 | 0 | 0 | 0 | 1.43 | 4.41 |
|  | Carpet/Mat | 0 | 0 | 0 | 0 | 0 | 0 | 0 | 0 | 0 | 0 |
|  | Clothes | 0 | 0 | 0 | 0.02 | 0.06 | 0 | 0 | 0.25 | 0.23 | 0.52 |
|  | Electronics | 0 | 0 | 0 | 0.01 | 0.09 | 0 | 0 | 0 | 0.04 | 0.17 |
|  | Fabric_Toy | 0 | 0 | 0 | 0.04 | 0.12 | 0 | 0 | 0 | 0.22 | 0.52 |
|  | Fabric_Wall/Furniture | 0 | 0 | 0 | 0.01 | 0.06 | 0 | 0 | 0 | 0.23 | 0.73 |
|  | Food-Container | 0.84 | 0.11 | 2.64 | 2.64 | 4.35 | 4.2 | 0.94 | 8.66 | 6.48 | 7.55 |
|  | Footwear | 0 | 0 | 0 | 0 | 0 | 0 | 0 | 0 | 0 | 0 |
|  | Hair/Body | 0 | 0 | 0 | 1.3 | 4.33 | 0 | 0 | 0.28 | 0.61 | 1.44 |
|  | Hands | 0.58 | 0.11 | 2.31 | 1.48 | 1.92 | 4.28 | 1.42 | 8 | 5.64 | 5.67 |
|  | Hard_Toy | 0 | 0 | 0 | 0.02 | 0.06 | 0 | 0 | 0 | 0.27 | 0.86 |
|  | Metal_Tool/Appliance | 0 | 0 | 0 | 0 | 0 | 0 | 0 | 0 | 0.01 | 0.11 |
|  | Metal_Wall/Furniture | 0 | 0 | 0 | 0 | 0.02 | 0 | 0 | 0 | 0.04 | 0.25 |
|  | Nose | 0 | 0 | 0 | 0 | 0 | 0 | 0 | 0 | 0 | 0 |
|  | Nothing | 51.59 | 41.38 | 55.15 | 47.27 | 11.95 | 37.96 | 27.92 | 56.04 | 45.45 | 25.95 |
|  | Other_Food | 2.21 | 0.32 | 4.36 | 2.7 | 2.81 | 6.25 | 1.36 | 10.94 | 7.58 | 7.33 |
|  | Pacifier | 0 | 0 | 0 | 3.22 | 10.77 | 0 | 0 | 0 | 1.64 | 6.12 |
|  | Paper/Wrapper | 0 | 0 | 0 | 0.02 | 0.08 | 0 | 0 | 0 | 0.2 | 0.52 |
|  | Plastic_Wall/Furniture | 0 | 0 | 0 | 0 | 0.01 | 0 | 0 | 0 | 0.01 | 0.07 |
|  | Plastic-Tool/Appliance | 0 | 0 | 0 | 0.04 | 0.24 | 0 | 0 | 0 | 0.16 | 0.51 |
|  | Porous-Plastic-Toy | 0 | 0 | 0.14 | 0.35 | 1.46 | 0 | 0 | 0.95 | 1.23 | 4.89 |
|  | Sticky_Food | 0 | 0 | 0 | 0.68 | 2.31 | 0 | 0 | 0 | 2.22 | 7.6 |
|  | Water | 0 | 0 | 0 | 0 | 0.02 | 0 | 0 | 0 | 0.1 | 0.42 |
|  | Wood_Toy | 0 | 0 | 0 | 0 | 0 | 0 | 0 | 0 | 0 | 0 |
|  | Wood_Wall/Furniture | 0 | 0 | 0 | 0 | 0 | 0 | 0 | 0 | 0 | 0 |
|  | **Dietary Total** | 3.01 | 0.9 | 4.55 | 3.54 | 4.1 | 8.12 | 2.92 | 16 | 11.26 | 12.01 |
|  | **Non-Dietary Total** | 5.38 | 1.47 | 11.78 | 9.2 | 11.88 | 13.76 | 7.84 | 21.49 | 17.46 | 15.48 |
|  | **Total Active Mouthing** | 8.41 | 4.85 | 18.64 | 12.73 | 11.95 | 23.6 | 13.98 | 37.61 | 28.72 | 23.14 |

*Duration in min/h; Frequency in contacts/h. Observation time = 255.00 hours.*

**Supplemental Table S7. Distribution of indoor object/surface hourly mouthing duration and frequency by Region.**

| **Group** | **Object/Surface** | **Med Dur** | **Q25 Dur** | **Q75 Dur** | **Mean Dur** | **SD Dur** | **Med Freq** | **Q25 Freq** | **Q75 Freq** | **Mean Freq** | **SD Freq** |
| --- | --- | --- | --- | --- | --- | --- | --- | --- | --- | --- | --- |
| ***Arizona (n=33)*** | Animal | 0 | 0 | 0 | 0 | 0 | 0 | 0 | 0 | 0 | 0 |
|  | Bedding/Towels | 0 | 0 | 0 | 0.01 | 0.04 | 0 | 0 | 0 | 0.11 | 0.33 |
|  | Beverage | 0 | 0 | 0.19 | 0.28 | 0.6 | 0 | 0 | 1.76 | 2.33 | 5.29 |
|  | Carpet/Mat | 0 | 0 | 0 | 0 | 0 | 0 | 0 | 0 | 0 | 0 |
|  | Clothes | 0 | 0 | 0.01 | 0.28 | 1.57 | 0 | 0 | 0.33 | 0.44 | 1.41 |
|  | Electronics | 0 | 0 | 0 | 0.02 | 0.11 | 0 | 0 | 0 | 0.05 | 0.2 |
|  | Fabric_Toy | 0 | 0 | 0 | 0.04 | 0.11 | 0 | 0 | 0 | 0.2 | 0.44 |
|  | Fabric_Wall/Furniture | 0 | 0 | 0 | 0 | 0.01 | 0 | 0 | 0 | 0.06 | 0.15 |
|  | Food-Container | 0.69 | 0.05 | 1.41 | 1.3 | 2.22 | 2.66 | 0.35 | 8.06 | 4.58 | 5.32 |
|  | Footwear | 0 | 0 | 0 | 0 | 0 | 0 | 0 | 0 | 0 | 0 |
|  | Hair/Body | 0 | 0 | 0.01 | 0.9 | 2.54 | 0 | 0 | 0.31 | 0.51 | 1.18 |
|  | Hands | 0.53 | 0.12 | 2.82 | 1.63 | 2.12 | 4.95 | 2.16 | 8.14 | 6.27 | 6.23 |
|  | Hard_Toy | 0 | 0 | 0 | 0.01 | 0.04 | 0 | 0 | 0 | 0.2 | 0.75 |
|  | Metal_Tool/Appliance | 0 | 0 | 0 | 0 | 0 | 0 | 0 | 0 | 0 | 0 |
|  | Metal_Wall/Furniture | 0 | 0 | 0 | 0.01 | 0.03 | 0 | 0 | 0 | 0.07 | 0.33 |
|  | Nose | 0 | 0 | 0 | 0 | 0 | 0 | 0 | 0 | 0 | 0 |
|  | Nothing | 51.91 | 48.95 | 55.17 | 50.63 | 7.63 | 41.93 | 35.34 | 57.55 | 47.93 | 22.9 |
|  | Other_Food | 1.62 | 0.92 | 4.32 | 2.77 | 2.68 | 6.27 | 3.39 | 8.67 | 6.79 | 5.54 |
|  | Pacifier | 0 | 0 | 0 | 1.31 | 6.17 | 0 | 0 | 0 | 0.89 | 4.39 |
|  | Paper/Wrapper | 0 | 0 | 0.01 | 0.03 | 0.09 | 0 | 0 | 0.29 | 0.24 | 0.54 |
|  | Plastic_Wall/Furniture | 0 | 0 | 0 | 0 | 0.02 | 0 | 0 | 0 | 0.01 | 0.07 |
|  | Plastic-Tool/Appliance | 0 | 0 | 0 | 0.01 | 0.04 | 0 | 0 | 0 | 0.12 | 0.34 |
|  | Porous-Plastic-Toy | 0 | 0 | 0.17 | 0.2 | 0.55 | 0 | 0 | 0.78 | 0.52 | 0.75 |
|  | Sticky_Food | 0 | 0 | 0.46 | 0.57 | 1.79 | 0 | 0 | 1.08 | 1.25 | 2.89 |
|  | Water | 0 | 0 | 0 | 0 | 0 | 0 | 0 | 0 | 0.02 | 0.09 |
|  | Wood_Toy | 0 | 0 | 0 | 0 | 0 | 0 | 0 | 0 | 0 | 0 |
|  | Wood_Wall/Furniture | 0 | 0 | 0 | 0 | 0 | 0 | 0 | 0 | 0 | 0 |
|  | **Dietary Total** | 3.09 | 1.28 | 4.61 | 3.61 | 3.24 | 8.67 | 5.92 | 14.14 | 10.38 | 8.02 |
|  | **Non-Dietary Total** | 2.26 | 0.74 | 9.28 | 5.76 | 7.63 | 11.8 | 7.55 | 19.83 | 14.3 | 10.68 |
|  | **Total Active Mouthing** | 8.09 | 4.83 | 11.05 | 9.37 | 7.63 | 21.26 | 15.64 | 27.82 | 24.68 | 15.61 |
| ***Florida (n=33)*** | Animal | 0 | 0 | 0 | 0 | 0.01 | 0 | 0 | 0 | 0.02 | 0.1 |
|  | Bedding/Towels | 0 | 0 | 0 | 0.04 | 0.13 | 0 | 0 | 0 | 0.41 | 1.28 |
|  | Beverage | 0 | 0 | 0 | 0.15 | 0.58 | 0 | 0 | 0 | 0.69 | 2.01 |
|  | Carpet/Mat | 0 | 0 | 0 | 0 | 0 | 0 | 0 | 0 | 0 | 0 |
|  | Clothes | 0 | 0 | 0.01 | 0.09 | 0.44 | 0 | 0 | 0.3 | 0.26 | 0.61 |
|  | Electronics | 0 | 0 | 0 | 0 | 0.01 | 0 | 0 | 0 | 0.03 | 0.11 |
|  | Fabric_Toy | 0 | 0 | 0 | 0.04 | 0.15 | 0 | 0 | 0 | 0.21 | 0.54 |
|  | Fabric_Wall/Furniture | 0 | 0 | 0 | 0.01 | 0.03 | 0 | 0 | 0 | 0.21 | 0.47 |
|  | Food-Container | 1.01 | 0.11 | 2.14 | 2.68 | 4.67 | 4.45 | 0.92 | 12.04 | 7.82 | 8.64 |
|  | Footwear | 0 | 0 | 0 | 0 | 0 | 0 | 0 | 0 | 0 | 0 |
|  | Hair/Body | 0 | 0 | 0 | 1.28 | 4.94 | 0 | 0 | 0 | 0.69 | 1.69 |
|  | Hands | 0.35 | 0.08 | 1.26 | 0.8 | 1.1 | 3.78 | 1.6 | 6.78 | 4.74 | 4.4 |
|  | Hard_Toy | 0 | 0 | 0 | 0 | 0.02 | 0 | 0 | 0 | 0.11 | 0.55 |
|  | Metal_Tool/Appliance | 0 | 0 | 0 | 0 | 0 | 0 | 0 | 0 | 0 | 0 |
|  | Metal_Wall/Furniture | 0 | 0 | 0 | 0 | 0 | 0 | 0 | 0 | 0.01 | 0.05 |
|  | Nose | 0 | 0 | 0 | 0 | 0 | 0 | 0 | 0 | 0 | 0 |
|  | Nothing | 52.85 | 40.41 | 55.58 | 47.33 | 12.28 | 39.35 | 26.5 | 56.95 | 46.51 | 30.42 |
|  | Other_Food | 2.34 | 0.32 | 3.72 | 2.93 | 3.37 | 6.31 | 1.43 | 12.59 | 8.37 | 7.81 |
|  | Pacifier | 0 | 0 | 0 | 3.33 | 11.53 | 0 | 0 | 0 | 0.63 | 2.5 |
|  | Paper/Wrapper | 0 | 0 | 0.02 | 0.12 | 0.61 | 0 | 0 | 0.32 | 0.56 | 1.64 |
|  | Plastic_Wall/Furniture | 0 | 0 | 0 | 0 | 0.01 | 0 | 0 | 0 | 0.01 | 0.06 |
|  | Plastic-Tool/Appliance | 0 | 0 | 0 | 0 | 0.02 | 0 | 0 | 0 | 0.04 | 0.2 |
|  | Porous-Plastic-Toy | 0 | 0 | 0.02 | 0.48 | 1.79 | 0 | 0 | 0.37 | 2.38 | 6.84 |
|  | Sticky_Food | 0 | 0 | 0.02 | 0.67 | 2.28 | 0 | 0 | 0.37 | 2.9 | 9.08 |
|  | Water | 0 | 0 | 0 | 0 | 0.02 | 0 | 0 | 0 | 0.13 | 0.51 |
|  | Wood_Toy | 0 | 0 | 0 | 0 | 0.01 | 0 | 0 | 0 | 0.01 | 0.06 |
|  | Wood_Wall/Furniture | 0 | 0 | 0 | 0.02 | 0.11 | 0 | 0 | 0 | 0.03 | 0.18 |
|  | **Dietary Total** | 2.96 | 0.41 | 3.95 | 3.75 | 4.77 | 8.47 | 3.74 | 16.06 | 11.99 | 12.81 |
|  | **Non-Dietary Total** | 3.94 | 1.18 | 15.82 | 8.92 | 12.18 | 14.48 | 8.09 | 26.38 | 18.36 | 16.12 |
|  | **Total Active Mouthing** | 7.15 | 4.42 | 19.59 | 12.67 | 12.28 | 25.42 | 14.12 | 42.45 | 30.35 | 24.46 |
| ***North Carolina (n=33)*** | Animal | 0 | 0 | 0 | 0 | 0 | 0 | 0 | 0 | 0.02 | 0.1 |
|  | Bedding/Towels | 0 | 0 | 0.04 | 0.9 | 2.85 | 0 | 0 | 0.68 | 2.32 | 6.61 |
|  | Beverage | 0 | 0 | 0 | 0.09 | 0.31 | 0 | 0 | 0 | 0.32 | 1.15 |
|  | Carpet/Mat | 0 | 0 | 0 | 0 | 0.02 | 0 | 0 | 0 | 0.02 | 0.12 |
|  | Clothes | 0 | 0 | 0.01 | 0.04 | 0.12 | 0 | 0 | 0.5 | 0.43 | 0.8 |
|  | Electronics | 0 | 0 | 0 | 0.01 | 0.06 | 0 | 0 | 0 | 0.08 | 0.26 |
|  | Fabric_Toy | 0 | 0 | 0 | 0 | 0.01 | 0 | 0 | 0 | 0.02 | 0.12 |
|  | Fabric_Wall/Furniture | 0 | 0 | 0 | 0.04 | 0.12 | 0 | 0 | 0 | 0.31 | 0.92 |
|  | Food-Container | 0.82 | 0.13 | 2.16 | 2.15 | 3.47 | 3.75 | 1.42 | 8.59 | 6.02 | 7.09 |
|  | Footwear | 0 | 0 | 0 | 0 | 0 | 0 | 0 | 0 | 0.05 | 0.26 |
|  | Hair/Body | 0 | 0 | 0.03 | 0.7 | 2.92 | 0 | 0 | 0.71 | 0.67 | 1.36 |
|  | Hands | 1.35 | 0.17 | 2.88 | 2.83 | 4.59 | 6.24 | 1.86 | 14.48 | 10.67 | 10.86 |
|  | Hard_Toy | 0 | 0 | 0 | 0.04 | 0.12 | 0 | 0 | 0 | 0.24 | 0.68 |
|  | Metal_Tool/Appliance | 0 | 0 | 0 | 0 | 0 | 0 | 0 | 0 | 0.02 | 0.13 |
|  | Metal_Wall/Furniture | 0 | 0 | 0 | 0 | 0 | 0 | 0 | 0 | 0 | 0 |
|  | Nose | 0 | 0 | 0 | 0 | 0 | 0 | 0 | 0 | 0.02 | 0.08 |
|  | Nothing | 50.34 | 41.94 | 55.64 | 47.28 | 11.35 | 42.1 | 27.8 | 54.01 | 48.28 | 35.5 |
|  | Other_Food | 0.97 | 0 | 3.34 | 2.33 | 3.19 | 3.06 | 0 | 9.04 | 7.4 | 10.03 |
|  | Pacifier | 0 | 0 | 0 | 2.62 | 6.54 | 0 | 0 | 0 | 1.68 | 6.09 |
|  | Paper/Wrapper | 0 | 0 | 0 | 0.08 | 0.32 | 0 | 0 | 0 | 0.7 | 2.01 |
|  | Plastic_Wall/Furniture | 0 | 0 | 0 | 0 | 0 | 0 | 0 | 0 | 0 | 0 |
|  | Plastic-Tool/Appliance | 0 | 0 | 0 | 0.08 | 0.33 | 0 | 0 | 0 | 0.32 | 1.24 |
|  | Porous-Plastic-Toy | 0 | 0 | 0.16 | 0.24 | 0.61 | 0 | 0 | 1.58 | 1.07 | 1.7 |
|  | Sticky_Food | 0 | 0 | 0 | 0.54 | 2.16 | 0 | 0 | 0 | 0.88 | 2.33 |
|  | Water | 0 | 0 | 0 | 0 | 0 | 0 | 0 | 0 | 0 | 0 |
|  | Wood_Toy | 0 | 0 | 0 | 0 | 0 | 0 | 0 | 0 | 0 | 0 |
|  | Wood_Wall/Furniture | 0 | 0 | 0 | 0 | 0 | 0 | 0 | 0 | 0 | 0 |
|  | **Dietary Total** | 2.13 | 0.08 | 4.36 | 2.94 | 3.63 | 5.14 | 0.8 | 12.71 | 8.58 | 9.96 |
|  | **Non-Dietary Total** | 4.3 | 2.44 | 14.8 | 9.93 | 10.47 | 16.68 | 11.52 | 38.28 | 24.51 | 19.77 |
|  | **Total Active Mouthing** | 9.66 | 4.36 | 18.06 | 12.87 | 11.5 | 23.46 | 13.21 | 51.32 | 33.09 | 27.08 |

*Duration in min/h; Frequency in contacts/h. Observation time = 255.00 hours.*

**Supplemental Table S8. Distribution of indoor object/surface hourly mouthing duration and frequency by Sex.**

| **Group** | **Object/Surface** | **Med Dur** | **Q25 Dur** | **Q75 Dur** | **Mean Dur** | **SD Dur** | **Med Freq** | **Q25 Freq** | **Q75 Freq** | **Mean Freq** | **SD Freq** |
| --- | --- | --- | --- | --- | --- | --- | --- | --- | --- | --- | --- |
| ***F (n=47)*** | Animal | 0 | 0 | 0 | 0 | 0.01 | 0 | 0 | 0 | 0.01 | 0.05 |
|  | Bedding/Towels | 0 | 0 | 0 | 0.04 | 0.13 | 0 | 0 | 0 | 0.17 | 0.46 |
|  | Beverage | 0 | 0 | 0.07 | 0.24 | 0.68 | 0 | 0 | 0.62 | 1.28 | 2.97 |
|  | Carpet/Mat | 0 | 0 | 0 | 0 | 0 | 0 | 0 | 0 | 0 | 0 |
|  | Clothes | 0 | 0 | 0 | 0.26 | 1.36 | 0 | 0 | 0.16 | 0.42 | 1.26 |
|  | Electronics | 0 | 0 | 0 | 0.02 | 0.1 | 0 | 0 | 0 | 0.05 | 0.21 |
|  | Fabric_Toy | 0 | 0 | 0 | 0.04 | 0.12 | 0 | 0 | 0 | 0.16 | 0.42 |
|  | Fabric_Wall/Furniture | 0 | 0 | 0 | 0.01 | 0.03 | 0 | 0 | 0 | 0.12 | 0.3 |
|  | Food-Container | 0.69 | 0.06 | 1.86 | 2.23 | 4.33 | 4.13 | 0.86 | 8.16 | 5.2 | 5.55 |
|  | Footwear | 0 | 0 | 0 | 0 | 0 | 0 | 0 | 0 | 0.01 | 0.05 |
|  | Hair/Body | 0 | 0 | 0.02 | 1.45 | 4.62 | 0 | 0 | 0.41 | 0.69 | 1.37 |
|  | Hands | 0.53 | 0.14 | 2.09 | 1.32 | 1.81 | 4.31 | 1.39 | 8.08 | 6.19 | 6.38 |
|  | Hard_Toy | 0 | 0 | 0 | 0.01 | 0.06 | 0 | 0 | 0 | 0.19 | 0.68 |
|  | Metal_Tool/Appliance | 0 | 0 | 0 | 0 | 0 | 0 | 0 | 0 | 0 | 0 |
|  | Metal_Wall/Furniture | 0 | 0 | 0 | 0 | 0.02 | 0 | 0 | 0 | 0.05 | 0.27 |
|  | Nose | 0 | 0 | 0 | 0 | 0 | 0 | 0 | 0 | 0 | 0 |
|  | Nothing | 50.93 | 42.79 | 55.19 | 48.3 | 10.2 | 38.3 | 24.37 | 57.45 | 47.81 | 37.2 |
|  | Other_Food | 1.56 | 0.04 | 3.1 | 2.17 | 2.59 | 4.72 | 0.27 | 8.33 | 6.21 | 6.96 |
|  | Pacifier | 0 | 0 | 0 | 3.29 | 9.49 | 0 | 0 | 0 | 1.39 | 4.44 |
|  | Paper/Wrapper | 0 | 0 | 0.01 | 0.1 | 0.51 | 0 | 0 | 0.3 | 0.45 | 1.5 |
|  | Plastic_Wall/Furniture | 0 | 0 | 0 | 0 | 0.01 | 0 | 0 | 0 | 0.01 | 0.07 |
|  | Plastic-Tool/Appliance | 0 | 0 | 0 | 0 | 0.01 | 0 | 0 | 0 | 0.06 | 0.22 |
|  | Porous-Plastic-Toy | 0 | 0 | 0.14 | 0.19 | 0.5 | 0 | 0 | 1.12 | 1.23 | 3.13 |
|  | Sticky_Food | 0 | 0 | 0 | 0.29 | 0.66 | 0 | 0 | 0 | 1.5 | 4.74 |
|  | Water | 0 | 0 | 0 | 0 | 0 | 0 | 0 | 0 | 0.02 | 0.11 |
|  | Wood_Toy | 0 | 0 | 0 | 0 | 0.01 | 0 | 0 | 0 | 0.01 | 0.05 |
|  | Wood_Wall/Furniture | 0 | 0 | 0 | 0.01 | 0.09 | 0 | 0 | 0 | 0.02 | 0.15 |
|  | **Dietary Total** | 2.36 | 0.35 | 3.58 | 2.68 | 2.8 | 7.75 | 0.99 | 12.23 | 8.98 | 8.33 |
|  | **Non-Dietary Total** | 4 | 1.46 | 15.46 | 9.12 | 10.61 | 12.96 | 8.47 | 20.64 | 16.38 | 12.33 |
|  | **Total Active Mouthing** | 9.03 | 4.8 | 17.21 | 11.8 | 10.33 | 19.56 | 13.74 | 28.94 | 25.36 | 17.78 |
| ***M (n=51)*** | Animal | 0 | 0 | 0 | 0 | 0 | 0 | 0 | 0 | 0.02 | 0.1 |
|  | Bedding/Towels | 0 | 0 | 0.01 | 0.59 | 2.32 | 0 | 0 | 0.33 | 1.68 | 5.45 |
|  | Beverage | 0 | 0 | 0 | 0.11 | 0.29 | 0 | 0 | 0 | 0.98 | 3.84 |
|  | Carpet/Mat | 0 | 0 | 0 | 0 | 0.02 | 0 | 0 | 0 | 0.01 | 0.1 |
|  | Clothes | 0 | 0 | 0.01 | 0.03 | 0.1 | 0 | 0 | 0.45 | 0.35 | 0.68 |
|  | Electronics | 0 | 0 | 0 | 0 | 0.01 | 0 | 0 | 0 | 0.05 | 0.19 |
|  | Fabric_Toy | 0 | 0 | 0 | 0.02 | 0.09 | 0 | 0 | 0 | 0.11 | 0.39 |
|  | Fabric_Wall/Furniture | 0 | 0 | 0 | 0.03 | 0.1 | 0 | 0 | 0 | 0.27 | 0.79 |
|  | Food-Container | 0.82 | 0.28 | 2.13 | 1.91 | 2.84 | 3.81 | 0.96 | 10.91 | 7.13 | 8.41 |
|  | Footwear | 0 | 0 | 0 | 0 | 0 | 0 | 0 | 0 | 0.03 | 0.21 |
|  | Hair/Body | 0 | 0 | 0.01 | 0.53 | 2.3 | 0 | 0 | 0.34 | 0.58 | 1.48 |
|  | Hands | 0.69 | 0.11 | 1.94 | 2.19 | 3.88 | 5.24 | 2.03 | 12.24 | 8.32 | 9.19 |
|  | Hard_Toy | 0 | 0 | 0 | 0.02 | 0.09 | 0 | 0 | 0 | 0.18 | 0.66 |
|  | Metal_Tool/Appliance | 0 | 0 | 0 | 0 | 0 | 0 | 0 | 0 | 0.01 | 0.1 |
|  | Metal_Wall/Furniture | 0 | 0 | 0 | 0 | 0 | 0 | 0 | 0 | 0.01 | 0.04 |
|  | Nose | 0 | 0 | 0 | 0 | 0 | 0 | 0 | 0 | 0.01 | 0.07 |
|  | Nothing | 52.99 | 46.49 | 55.83 | 48.5 | 11.19 | 46.32 | 34.02 | 56.33 | 47.59 | 21.43 |
|  | Other_Food | 2.26 | 0.51 | 4.71 | 3.18 | 3.44 | 6.5 | 2.3 | 12.28 | 8.83 | 8.69 |
|  | Pacifier | 0 | 0 | 0 | 1.66 | 7.35 | 0 | 0 | 0 | 0.79 | 4.7 |
|  | Paper/Wrapper | 0 | 0 | 0 | 0.06 | 0.26 | 0 | 0 | 0 | 0.51 | 1.56 |
|  | Plastic_Wall/Furniture | 0 | 0 | 0 | 0 | 0 | 0 | 0 | 0 | 0 | 0 |
|  | Plastic-Tool/Appliance | 0 | 0 | 0 | 0.06 | 0.27 | 0 | 0 | 0 | 0.25 | 1.03 |
|  | Porous-Plastic-Toy | 0 | 0 | 0.09 | 0.41 | 1.5 | 0 | 0 | 0.76 | 1.43 | 4.93 |
|  | Sticky_Food | 0 | 0 | 0.01 | 0.68 | 2.47 | 0 | 0 | 0.35 | 1.64 | 6.35 |
|  | Water | 0 | 0 | 0 | 0 | 0.02 | 0 | 0 | 0 | 0.08 | 0.41 |
|  | Wood_Toy | 0 | 0 | 0 | 0 | 0 | 0 | 0 | 0 | 0 | 0 |
|  | Wood_Wall/Furniture | 0 | 0 | 0 | 0 | 0 | 0 | 0 | 0 | 0 | 0 |
|  | **Dietary Total** | 3.23 | 0.82 | 4.86 | 3.98 | 4.56 | 8.07 | 3.57 | 15.68 | 11.47 | 12.1 |
|  | **Non-Dietary Total** | 3.68 | 1.31 | 8.64 | 7.52 | 10.13 | 17.07 | 8.4 | 27.91 | 21.83 | 19.11 |
|  | **Total Active Mouthing** | 7.01 | 4.17 | 13.51 | 11.49 | 11.19 | 26.14 | 13.84 | 44.14 | 33.3 | 26.61 |
| ***O (n=1)*** | Animal | 0 | 0 | 0 | 0 | NA | 0 | 0 | 0 | 0 | NA |
|  | Bedding/Towels | 0 | 0 | 0 | 0 | NA | 0 | 0 | 0 | 0 | NA |
|  | Beverage | 0 | 0 | 0 | 0 | NA | 0 | 0 | 0 | 0 | NA |
|  | Carpet/Mat | 0 | 0 | 0 | 0 | NA | 0 | 0 | 0 | 0 | NA |
|  | Clothes | 0 | 0 | 0 | 0 | NA | 0 | 0 | 0 | 0 | NA |
|  | Electronics | 0 | 0 | 0 | 0 | NA | 0 | 0 | 0 | 0 | NA |
|  | Fabric_Toy | 0.02 | 0.02 | 0.02 | 0.02 | NA | 1.18 | 1.18 | 1.18 | 1.18 | NA |
|  | Fabric_Wall/Furniture | 0 | 0 | 0 | 0 | NA | 0 | 0 | 0 | 0 | NA |
|  | Food-Container | 0 | 0 | 0 | 0 | NA | 0 | 0 | 0 | 0 | NA |
|  | Footwear | 0 | 0 | 0 | 0 | NA | 0 | 0 | 0 | 0 | NA |
|  | Hair/Body | 0 | 0 | 0 | 0 | NA | 0 | 0 | 0 | 0 | NA |
|  | Hands | 0 | 0 | 0 | 0 | NA | 0 | 0 | 0 | 0 | NA |
|  | Hard_Toy | 0 | 0 | 0 | 0 | NA | 0 | 0 | 0 | 0 | NA |
|  | Metal_Tool/Appliance | 0 | 0 | 0 | 0 | NA | 0 | 0 | 0 | 0 | NA |
|  | Metal_Wall/Furniture | 0 | 0 | 0 | 0 | NA | 0 | 0 | 0 | 0 | NA |
|  | Nose | 0 | 0 | 0 | 0 | NA | 0 | 0 | 0 | 0 | NA |
|  | Nothing | 48.95 | 48.95 | 48.95 | 48.95 | NA | 35.34 | 35.34 | 35.34 | 35.34 | NA |
|  | Other_Food | 0.88 | 0.88 | 0.88 | 0.88 | NA | 2.36 | 2.36 | 2.36 | 2.36 | NA |
|  | Pacifier | 0 | 0 | 0 | 0 | NA | 0 | 0 | 0 | 0 | NA |
|  | Paper/Wrapper | 0.06 | 0.06 | 0.06 | 0.06 | NA | 2.36 | 2.36 | 2.36 | 2.36 | NA |
|  | Plastic_Wall/Furniture | 0 | 0 | 0 | 0 | NA | 0 | 0 | 0 | 0 | NA |
|  | Plastic-Tool/Appliance | 0 | 0 | 0 | 0 | NA | 0 | 0 | 0 | 0 | NA |
|  | Porous-Plastic-Toy | 0 | 0 | 0 | 0 | NA | 0 | 0 | 0 | 0 | NA |
|  | Sticky_Food | 10.09 | 10.09 | 10.09 | 10.09 | NA | 11.78 | 11.78 | 11.78 | 11.78 | NA |
|  | Water | 0 | 0 | 0 | 0 | NA | 0 | 0 | 0 | 0 | NA |
|  | Wood_Toy | 0 | 0 | 0 | 0 | NA | 0 | 0 | 0 | 0 | NA |
|  | Wood_Wall/Furniture | 0 | 0 | 0 | 0 | NA | 0 | 0 | 0 | 0 | NA |
|  | **Dietary Total** | 10.98 | 10.98 | 10.98 | 10.98 | NA | 14.14 | 14.14 | 14.14 | 14.14 | NA |
|  | **Non-Dietary Total** | 0.08 | 0.08 | 0.08 | 0.08 | NA | 3.53 | 3.53 | 3.53 | 3.53 | NA |
|  | **Total Active Mouthing** | 11.05 | 11.05 | 11.05 | 11.05 | NA | 17.67 | 17.67 | 17.67 | 17.67 | NA |

*Duration in min/h; Frequency in contacts/h. Observation time = 255.00 hours.*

**Supplemental Table S9. Distribution of indoor object/surface hourly mouthing duration and frequency by Age Group.**

| **Group** | **Object/Surface** | **Med Dur** | **Q25 Dur** | **Q75 Dur** | **Mean Dur** | **SD Dur** | **Med Freq** | **Q25 Freq** | **Q75 Freq** | **Mean Freq** | **SD Freq** |
| --- | --- | --- | --- | --- | --- | --- | --- | --- | --- | --- | --- |
| ***12 to <24 months (n=25)*** | Animal | 0 | 0 | 0 | 0 | 0 | 0 | 0 | 0 | 0.02 | 0.12 |
|  | Bedding/Towels | 0 | 0 | 0 | 0.08 | 0.25 | 0 | 0 | 0 | 0.53 | 1.23 |
|  | Beverage | 0 | 0 | 0 | 0.06 | 0.23 | 0 | 0 | 0 | 0.61 | 2.36 |
|  | Carpet/Mat | 0 | 0 | 0 | 0 | 0 | 0 | 0 | 0 | 0 | 0 |
|  | Clothes | 0 | 0 | 0.01 | 0.13 | 0.51 | 0 | 0 | 0.39 | 0.34 | 0.69 |
|  | Electronics | 0 | 0 | 0 | 0.03 | 0.12 | 0 | 0 | 0 | 0.08 | 0.24 |
|  | Fabric_Toy | 0 | 0 | 0 | 0.01 | 0.04 | 0 | 0 | 0 | 0.18 | 0.35 |
|  | Fabric_Wall/Furniture | 0 | 0 | 0 | 0.02 | 0.08 | 0 | 0 | 0 | 0.28 | 0.94 |
|  | Food-Container | 0.93 | 0.11 | 1.36 | 1.85 | 3.33 | 5.27 | 1.06 | 10.84 | 6.49 | 6.23 |
|  | Footwear | 0 | 0 | 0 | 0 | 0 | 0 | 0 | 0 | 0.07 | 0.3 |
|  | Hair/Body | 0 | 0 | 0.13 | 1.68 | 4.05 | 0 | 0 | 2.34 | 1.33 | 2.2 |
|  | Hands | 0.7 | 0.16 | 1.45 | 1.27 | 1.78 | 6.78 | 1.34 | 14.48 | 9.55 | 9.52 |
|  | Hard_Toy | 0 | 0 | 0 | 0.02 | 0.08 | 0 | 0 | 0 | 0.3 | 0.91 |
|  | Metal_Tool/Appliance | 0 | 0 | 0 | 0 | 0 | 0 | 0 | 0 | 0.03 | 0.15 |
|  | Metal_Wall/Furniture | 0 | 0 | 0 | 0.01 | 0.03 | 0 | 0 | 0 | 0.07 | 0.35 |
|  | Nose | 0 | 0 | 0 | 0 | 0 | 0 | 0 | 0 | 0 | 0 |
|  | Nothing | 51.25 | 47.24 | 54.74 | 48.41 | 10.41 | 46.23 | 35.34 | 56.95 | 50.9 | 22.98 |
|  | Other_Food | 2.96 | 0.92 | 4.03 | 2.65 | 1.91 | 7.42 | 3.39 | 14.06 | 9.41 | 7.65 |
|  | Pacifier | 0 | 0 | 0 | 2.73 | 8.38 | 0 | 0 | 0 | 2.67 | 8.26 |
|  | Paper/Wrapper | 0 | 0 | 0 | 0.04 | 0.1 | 0 | 0 | 0 | 0.57 | 1.55 |
|  | Plastic_Wall/Furniture | 0 | 0 | 0 | 0 | 0.02 | 0 | 0 | 0 | 0.02 | 0.08 |
|  | Plastic-Tool/Appliance | 0 | 0 | 0 | 0.11 | 0.37 | 0 | 0 | 0 | 0.46 | 1.42 |
|  | Porous-Plastic-Toy | 0.01 | 0 | 0.22 | 0.19 | 0.37 | 0.34 | 0 | 1.46 | 1.54 | 3.34 |
|  | Sticky_Food | 0 | 0 | 0 | 0.71 | 2.07 | 0 | 0 | 0 | 1.18 | 2.8 |
|  | Water | 0 | 0 | 0 | 0 | 0 | 0 | 0 | 0 | 0.02 | 0.09 |
|  | Wood_Toy | 0 | 0 | 0 | 0 | 0 | 0 | 0 | 0 | 0 | 0 |
|  | Wood_Wall/Furniture | 0 | 0 | 0 | 0 | 0 | 0 | 0 | 0 | 0 | 0 |
|  | **Dietary Total** | 3.24 | 1.04 | 4.37 | 3.39 | 2.64 | 10.41 | 5.99 | 18.31 | 11.14 | 8.01 |
|  | **Non-Dietary Total** | 3.94 | 1.69 | 9.64 | 8.4 | 10.35 | 16.24 | 10.21 | 38.28 | 24.37 | 19.72 |
|  | **Total Active Mouthing** | 8.74 | 5.26 | 12.76 | 11.78 | 10.65 | 27.79 | 16.49 | 51.32 | 35.51 | 25.13 |
| ***24 to <36 months (n=24)*** | Animal | 0 | 0 | 0 | 0 | 0 | 0 | 0 | 0 | 0.02 | 0.09 |
|  | Bedding/Towels | 0 | 0 | 0 | 0.02 | 0.04 | 0 | 0 | 0 | 0.13 | 0.31 |
|  | Beverage | 0 | 0 | 0.19 | 0.34 | 0.77 | 0 | 0 | 1.35 | 2.54 | 5.97 |
|  | Carpet/Mat | 0 | 0 | 0 | 0 | 0 | 0 | 0 | 0 | 0 | 0 |
|  | Clothes | 0 | 0 | 0 | 0.39 | 1.84 | 0 | 0 | 0.08 | 0.5 | 1.6 |
|  | Electronics | 0 | 0 | 0 | 0 | 0 | 0 | 0 | 0 | 0 | 0 |
|  | Fabric_Toy | 0 | 0 | 0 | 0.03 | 0.11 | 0 | 0 | 0 | 0.2 | 0.54 |
|  | Fabric_Wall/Furniture | 0 | 0 | 0 | 0.02 | 0.06 | 0 | 0 | 0.08 | 0.17 | 0.41 |
|  | Food-Container | 0.8 | 0.22 | 2.2 | 1.99 | 2.77 | 3.97 | 1.24 | 8.22 | 5.78 | 6.78 |
|  | Footwear | 0 | 0 | 0 | 0 | 0 | 0 | 0 | 0 | 0 | 0 |
|  | Hair/Body | 0 | 0 | 0 | 0.15 | 0.64 | 0 | 0 | 0 | 0.44 | 1.25 |
|  | Hands | 0.72 | 0.11 | 2.91 | 1.71 | 2.13 | 5.49 | 1.48 | 8.61 | 6.51 | 6.22 |
|  | Hard_Toy | 0 | 0 | 0 | 0.02 | 0.1 | 0 | 0 | 0 | 0.07 | 0.33 |
|  | Metal_Tool/Appliance | 0 | 0 | 0 | 0 | 0 | 0 | 0 | 0 | 0 | 0 |
|  | Metal_Wall/Furniture | 0 | 0 | 0 | 0 | 0.01 | 0 | 0 | 0 | 0.03 | 0.15 |
|  | Nose | 0 | 0 | 0 | 0 | 0 | 0 | 0 | 0 | 0.01 | 0.07 |
|  | Nothing | 51.13 | 46.04 | 55.3 | 48.11 | 12.9 | 38.18 | 32.3 | 55.65 | 47.98 | 31.13 |
|  | Other_Food | 2.08 | 0.05 | 4.44 | 2.48 | 2.36 | 5.5 | 0.48 | 9.15 | 7.26 | 8.14 |
|  | Pacifier | 0 | 0 | 0 | 4.1 | 13.21 | 0 | 0 | 0 | 0.84 | 2.94 |
|  | Paper/Wrapper | 0 | 0 | 0 | 0.11 | 0.37 | 0 | 0 | 0.07 | 0.61 | 1.87 |
|  | Plastic_Wall/Furniture | 0 | 0 | 0 | 0 | 0 | 0 | 0 | 0 | 0 | 0 |
|  | Plastic-Tool/Appliance | 0 | 0 | 0 | 0 | 0.02 | 0 | 0 | 0 | 0.03 | 0.09 |
|  | Porous-Plastic-Toy | 0 | 0 | 0.18 | 0.45 | 1.02 | 0 | 0 | 1.39 | 1.26 | 2.26 |
|  | Sticky_Food | 0 | 0 | 0 | 0.07 | 0.19 | 0 | 0 | 0 | 0.17 | 0.48 |
|  | Water | 0 | 0 | 0 | 0 | 0.02 | 0 | 0 | 0 | 0.13 | 0.58 |
|  | Wood_Toy | 0 | 0 | 0 | 0 | 0 | 0 | 0 | 0 | 0 | 0 |
|  | Wood_Wall/Furniture | 0 | 0 | 0 | 0 | 0 | 0 | 0 | 0 | 0 | 0 |
|  | **Dietary Total** | 2.63 | 0.79 | 4.47 | 2.89 | 2.39 | 8.12 | 2.49 | 12.34 | 10.01 | 10.04 |
|  | **Non-Dietary Total** | 4.79 | 1.37 | 9.64 | 9 | 12.89 | 15.77 | 7.44 | 21.78 | 16.75 | 12.01 |
|  | **Total Active Mouthing** | 8.87 | 4.7 | 13.95 | 11.89 | 12.9 | 22.53 | 12.51 | 35.43 | 26.77 | 19.43 |
| ***36 to <72 months (n=33)*** | Animal | 0 | 0 | 0 | 0 | 0 | 0 | 0 | 0 | 0 | 0 |
|  | Bedding/Towels | 0 | 0 | 0 | 0.71 | 2.78 | 0 | 0 | 0 | 1.9 | 6.57 |
|  | Beverage | 0 | 0 | 0.26 | 0.22 | 0.55 | 0 | 0 | 0.84 | 0.99 | 1.9 |
|  | Carpet/Mat | 0 | 0 | 0 | 0 | 0 | 0 | 0 | 0 | 0 | 0 |
|  | Clothes | 0 | 0 | 0.01 | 0.03 | 0.1 | 0 | 0 | 0.35 | 0.32 | 0.64 |
|  | Electronics | 0 | 0 | 0 | 0 | 0.01 | 0 | 0 | 0 | 0.05 | 0.22 |
|  | Fabric_Toy | 0 | 0 | 0 | 0 | 0 | 0 | 0 | 0 | 0 | 0 |
|  | Fabric_Wall/Furniture | 0 | 0 | 0 | 0.01 | 0.03 | 0 | 0 | 0 | 0.11 | 0.3 |
|  | Food-Container | 0.22 | 0 | 0.69 | 0.59 | 0.96 | 2.03 | 0 | 5.46 | 4.79 | 7.22 |
|  | Footwear | 0 | 0 | 0 | 0 | 0 | 0 | 0 | 0 | 0 | 0 |
|  | Hair/Body | 0 | 0 | 0 | 0.03 | 0.13 | 0 | 0 | 0.27 | 0.22 | 0.44 |
|  | Hands | 0.27 | 0.09 | 1.46 | 1.82 | 3.82 | 3.78 | 2.16 | 6.24 | 6.7 | 9.13 |
|  | Hard_Toy | 0 | 0 | 0 | 0.01 | 0.03 | 0 | 0 | 0 | 0.22 | 0.72 |
|  | Metal_Tool/Appliance | 0 | 0 | 0 | 0 | 0 | 0 | 0 | 0 | 0 | 0 |
|  | Metal_Wall/Furniture | 0 | 0 | 0 | 0 | 0 | 0 | 0 | 0 | 0.01 | 0.05 |
|  | Nose | 0 | 0 | 0 | 0 | 0 | 0 | 0 | 0 | 0.01 | 0.06 |
|  | Nothing | 54.98 | 50.14 | 56.6 | 51.34 | 9.19 | 46.41 | 35.87 | 57.95 | 54.83 | 34.29 |
|  | Other_Food | 2.48 | 0.75 | 4.94 | 3.94 | 4.23 | 4.65 | 1.84 | 8.76 | 7.32 | 8.23 |
|  | Pacifier | 0 | 0 | 0 | 0 | 0 | 0 | 0 | 0 | 0 | 0 |
|  | Paper/Wrapper | 0 | 0 | 0.01 | 0.13 | 0.61 | 0 | 0 | 0.31 | 0.52 | 1.61 |
|  | Plastic_Wall/Furniture | 0 | 0 | 0 | 0 | 0 | 0 | 0 | 0 | 0 | 0 |
|  | Plastic-Tool/Appliance | 0 | 0 | 0 | 0 | 0 | 0 | 0 | 0 | 0 | 0 |
|  | Porous-Plastic-Toy | 0 | 0 | 0 | 0.01 | 0.04 | 0 | 0 | 0 | 0.08 | 0.18 |
|  | Sticky_Food | 0 | 0 | 0.5 | 1.15 | 3.02 | 0 | 0 | 2.95 | 3.69 | 9.14 |
|  | Water | 0 | 0 | 0 | 0 | 0 | 0 | 0 | 0 | 0.02 | 0.13 |
|  | Wood_Toy | 0 | 0 | 0 | 0 | 0 | 0 | 0 | 0 | 0 | 0 |
|  | Wood_Wall/Furn | 0 | 0 | 0 | 0 | 0 | 0 | 0 | 0 | 0 | 0 |
|  | **Dietary Total** | 3.35 | 2.21 | 7.41 | 5.31 | 5.4 | 8.47 | 4.64 | 15.01 | 12.02 | 13.06 |
|  | **Non-Dietary Total** | 1.18 | 0.39 | 3.9 | 3.35 | 5.65 | 9.28 | 5.95 | 17.47 | 14.99 | 14.41 |
|  | **Total Active Mouthing** | 5.02 | 3.4 | 9.86 | 8.66 | 9.19 | 21.26 | 13.5 | 30.55 | 27 | 22.7 |
| ***6 to <12 months (n=17)*** | Animal | 0 | 0 | 0 | 0 | 0.01 | 0 | 0 | 0 | 0.02 | 0.08 |
|  | Bedding/Towels | 0 | 0 | 0.01 | 0.33 | 1.13 | 0 | 0 | 0.33 | 0.84 | 2.25 |
|  | Beverage | 0 | 0 | 0 | 0.01 | 0.03 | 0 | 0 | 0 | 0.07 | 0.24 |
|  | Carpet/Mat | 0 | 0 | 0 | 0.01 | 0.03 | 0 | 0 | 0 | 0.04 | 0.17 |
|  | Clothes | 0 | 0 | 0 | 0.02 | 0.08 | 0 | 0 | 0 | 0.38 | 0.9 |
|  | Electronics | 0 | 0 | 0 | 0.02 | 0.08 | 0 | 0 | 0 | 0.08 | 0.24 |
|  | Fabric_Toy | 0 | 0 | 0.01 | 0.09 | 0.21 | 0 | 0 | 0.37 | 0.29 | 0.6 |
|  | Fabric_Wall/Furniture | 0 | 0 | 0 | 0.04 | 0.12 | 0 | 0 | 0 | 0.26 | 0.67 |
|  | Food-Container | 2.47 | 1.46 | 5.9 | 5.23 | 5.91 | 5.24 | 1.42 | 13.59 | 8.75 | 8.8 |
|  | Footwear | 0 | 0 | 0 | 0 | 0 | 0 | 0 | 0 | 0 | 0 |
|  | Hair/Body | 0 | 0 | 1.45 | 2.86 | 6.83 | 0 | 0 | 0.78 | 0.64 | 1.08 |
|  | Hands | 1.66 | 0.18 | 2.37 | 2.4 | 4.08 | 5.24 | 1.86 | 8.9 | 5.84 | 4.6 |
|  | Hard_Toy | 0 | 0 | 0 | 0.02 | 0.08 | 0 | 0 | 0 | 0.11 | 0.45 |
|  | Metal_Tool/Appliance | 0 | 0 | 0 | 0 | 0 | 0 | 0 | 0 | 0 | 0 |
|  | Metal_Wall/Furniture | 0 | 0 | 0 | 0 | 0 | 0 | 0 | 0 | 0 | 0 |
|  | Nose | 0 | 0 | 0 | 0 | 0 | 0 | 0 | 0 | 0 | 0 |
|  | Nothing | 41.33 | 38.91 | 49.98 | 43.17 | 8.55 | 21.87 | 17.04 | 28.05 | 28.01 | 18.83 |
|  | Other_Food | 0.32 | 0 | 0.89 | 0.54 | 0.66 | 3.37 | 0 | 6.99 | 5.5 | 7.65 |
|  | Pacifier | 0 | 0 | 3.25 | 4.29 | 7.47 | 0 | 0 | 1.52 | 1.1 | 2.25 |
|  | Paper/Wrapper | 0 | 0 | 0.01 | 0.01 | 0.03 | 0 | 0 | 0.26 | 0.2 | 0.5 |
|  | Plastic_Wall/Furniture | 0 | 0 | 0 | 0 | 0.01 | 0 | 0 | 0 | 0.02 | 0.08 |
|  | Plastic-Tool/Appliance | 0 | 0 | 0 | 0.03 | 0.06 | 0 | 0 | 0 | 0.21 | 0.48 |
|  | Porous-Plastic-Toy | 0.03 | 0 | 0.37 | 0.82 | 2.38 | 0.37 | 0 | 1.97 | 3.5 | 8.47 |
|  | Sticky_Food | 0 | 0 | 0 | 0.08 | 0.28 | 0 | 0 | 0 | 0.64 | 2.21 |
|  | Water | 0 | 0 | 0 | 0 | 0 | 0 | 0 | 0 | 0.04 | 0.16 |
|  | Wood_Toy | 0 | 0 | 0 | 0 | 0.01 | 0 | 0 | 0 | 0.02 | 0.08 |
|  | Wood_Wall/Furniture | 0 | 0 | 0 | 0.04 | 0.16 | 0 | 0 | 0 | 0.06 | 0.25 |
|  | **Dietary Total** | 0.32 | 0 | 0.89 | 0.62 | 0.75 | 3.74 | 0 | 9.14 | 6.22 | 7.76 |
|  | **Non-Dietary Total** | 17.66 | 9.79 | 20.56 | 16.21 | 8.43 | 16.68 | 11.52 | 23.46 | 22.4 | 18.27 |
|  | **Total Active Mouthing** | 18.67 | 10.02 | 21.09 | 16.83 | 8.55 | 23.46 | 14.09 | 32.99 | 28.62 | 25.02 |

*Duration in min/h; Frequency in contacts/h. Observation time = 255.00 hours.*

**Supplemental Table S10. Kruskal-Wallis tests for surface mouthing by age group.**

| **Object/Surface** | **Metric** | **Test** | **Chi_squared** | **df** | **p_value** | **Significance** |
| --- | --- | --- | --- | --- | --- | --- |
| **Animal** | duration | Kruskal-Wallis | 3.022 | 3 | 0.3882 | ns |
|  | frequency | Kruskal-Wallis | 1.646 | 3 | 0.6491 | ns |
| **Bedding/Towels** | duration | Kruskal-Wallis | 1.465 | 3 | 0.6904 | ns |
|  | frequency | Kruskal-Wallis | 1.407 | 3 | 0.7038 | ns |
| **Beverage** | duration | Kruskal-Wallis | 5.02 | 3 | 0.1703 | ns |
|  | frequency | Kruskal-Wallis | 5.151 | 3 | 0.1611 | ns |
| **Carpet/Mat** | duration | Kruskal-Wallis | 4.824 | 3 | 0.1852 | ns |
|  | frequency | Kruskal-Wallis | 4.824 | 3 | 0.1852 | ns |
| **Clothes** | duration | Kruskal-Wallis | 0.382 | 3 | 0.9439 | ns |
|  | frequency | Kruskal-Wallis | 0.524 | 3 | 0.9136 | ns |
| **Electronics** | duration | Kruskal-Wallis | 3.296 | 3 | 0.3482 | ns |
|  | frequency | Kruskal-Wallis | 3.23 | 3 | 0.3575 | ns |
| **Fabric_Toy** | duration | Kruskal-Wallis | 10.628 | 3 | 0.0139 | * |
|  | frequency | Kruskal-Wallis | 10.075 | 3 | 0.0179 | * |
| **Fabric_Wall/Furniture** | duration | Kruskal-Wallis | 1.001 | 3 | 0.801 | ns |
|  | frequency | Kruskal-Wallis | 0.888 | 3 | 0.8283 | ns |
| **Food-Container** | duration | Kruskal-Wallis | 22.098 | 3 | < 0.001 | *** |
|  | frequency | Kruskal-Wallis | 5.442 | 3 | 0.1421 | ns |
| **Footwear** | duration | Kruskal-Wallis | 5.98 | 3 | 0.1126 | ns |
|  | frequency | Kruskal-Wallis | 5.98 | 3 | 0.1126 | ns |
| **Hair/Body** | duration | Kruskal-Wallis | 7.289 | 3 | 0.0632 | ns |
|  | frequency | Kruskal-Wallis | 5.763 | 3 | 0.1237 | ns |
| **Hands** | duration | Kruskal-Wallis | 2.404 | 3 | 0.4929 | ns |
|  | frequency | Kruskal-Wallis | 1.483 | 3 | 0.6861 | ns |
| **Hard_Toy** | duration | Kruskal-Wallis | 2.105 | 3 | 0.5508 | ns |
|  | frequency | Kruskal-Wallis | 2.341 | 3 | 0.5046 | ns |
| **Metal_Tool/Appliance** | duration | Kruskal-Wallis | NA | 3 | NA | NA |
|  | frequency | Kruskal-Wallis | 2.96 | 3 | 0.3978 | ns |
| **Metal_Wall/Furniture** | duration | Kruskal-Wallis | 0.723 | 3 | 0.8677 | ns |
|  | frequency | Kruskal-Wallis | 0.723 | 3 | 0.8677 | ns |
| **Nose** | duration | Kruskal-Wallis | NA | 3 | NA | NA |
|  | frequency | Kruskal-Wallis | 1.567 | 3 | 0.6668 | ns |
| **Nothing** | duration | Kruskal-Wallis | 12.124 | 3 | 0.007 | ** |
|  | frequency | Kruskal-Wallis | 16.256 | 3 | 0.001 | ** |
| **Other_Food** | duration | Kruskal-Wallis | 15.866 | 3 | 0.0012 | ** |
|  | frequency | Kruskal-Wallis | 4.717 | 3 | 0.1937 | ns |
| **Pacifier** | duration | Kruskal-Wallis | 12.094 | 3 | 0.0071 | ** |
|  | frequency | Kruskal-Wallis | 11.695 | 3 | 0.0085 | ** |
| **Paper/Wrapper** | duration | Kruskal-Wallis | 0.351 | 3 | 0.9502 | ns |
|  | frequency | Kruskal-Wallis | 0.314 | 3 | 0.9574 | ns |
| **Plastic_Wall/Furniture** | duration | Kruskal-Wallis | 2.903 | 3 | 0.4069 | ns |
|  | frequency | Kruskal-Wallis | 2.903 | 3 | 0.4069 | ns |
| **Plastic-Tool/Appliance** | duration | Kruskal-Wallis | 7.669 | 3 | 0.0534 | ns |
|  | frequency | Kruskal-Wallis | 7.832 | 3 | 0.0496 | * |
| **Porous-Plastic-Toy** | duration | Kruskal-Wallis | 14.578 | 3 | 0.0022 | ** |
|  | frequency | Kruskal-Wallis | 15.122 | 3 | 0.0017 | ** |
| **Sticky_Food** | duration | Kruskal-Wallis | 7.012 | 3 | 0.0715 | ns |
|  | frequency | Kruskal-Wallis | 7.898 | 3 | 0.0482 | * |
| **Water** | duration | Kruskal-Wallis | 0.215 | 3 | 0.9751 | ns |
|  | frequency | Kruskal-Wallis | 0.885 | 3 | 0.8291 | ns |
| **Wood_Toy** | duration | Kruskal-Wallis | 4.824 | 3 | 0.1852 | ns |
|  | frequency | Kruskal-Wallis | 4.824 | 3 | 0.1852 | ns |
| **Wood_Wall/Furniture** | duration | Kruskal-Wallis | 4.824 | 3 | 0.1852 | ns |
|  | frequency | Kruskal-Wallis | 4.824 | 3 | 0.1852 | ns |

*Duration in min/h; Frequency in contacts/h. * p < 0.05, ** p < 0.01, *** p < 0.001.*

**Supplemental Table S11. Distribution of time spent in indoor locations (min/h).**

| **Location** | **Mean** | **SD** | **Min** | **P5** | **P25** | **P50** | **P75** | **P95** | **Max** | **N** |
| --- | --- | --- | --- | --- | --- | --- | --- | --- | --- | --- |
| **Living Room** | 32.14 | 18.23 | 0 | 0.14 | 17.74 | 34.07 | 47.21 | 59 | 60 | 99 |
| **Bedroom** | 14.39 | 19.04 | 0 | 0 | 0 | 2.49 | 28.68 | 52.52 | 60 | 99 |
| **Dining Room** | 6.04 | 8.71 | 0 | 0 | 0 | 1.74 | 8.21 | 22.57 | 43.83 | 99 |
| **Kitchen** | 4.18 | 6.26 | 0 | 0 | 0.54 | 2.22 | 4.9 | 16.33 | 38.06 | 99 |
| **Playroom** | 1.74 | 6.72 | 0 | 0 | 0 | 0 | 0 | 11.82 | 40.62 | 99 |
| **Corridors** | 1.1 | 2.08 | 0 | 0 | 0.01 | 0.36 | 1.13 | 4.34 | 12.42 | 99 |
| **Bathroom** | 0.17 | 0.8 | 0 | 0 | 0 | 0 | 0 | 0.44 | 6.46 | 99 |
| **Den** | 0.13 | 0.92 | 0 | 0 | 0 | 0 | 0 | 0 | 7.85 | 99 |
| **Balcony/Porch** | 0.11 | 0.8 | 0 | 0 | 0 | 0 | 0 | 0.07 | 7.51 | 99 |
| **Laundry Room** | 0 | 0 | 0 | 0 | 0 | 0 | 0 | 0 | 0.01 | 99 |

*Values in min/h. Observation time = 273.10 hours (Location Not-In-View excluded). Sorted by mean (descending).*

**Supplemental Table S12. Distribution of location visit frequency (visits/h).**

| **Location** | **Mean** | **SD** | **Min** | **P5** | **P25** | **P50** | **P75** | **P95** | **Max** | **N** |
| --- | --- | --- | --- | --- | --- | --- | --- | --- | --- | --- |
| **Living Room** | 35.13 | 29.83 | 0 | 0.44 | 13.47 | 27.67 | 46.92 | 100.29 | 143.97 | 99 |
| **Dining Room** | 15.26 | 19.85 | 0 | 0 | 0 | 7.59 | 25.28 | 59.13 | 88.59 | 99 |
| **Bedroom** | 9.94 | 21.12 | 0 | 0 | 0 | 3.35 | 9.96 | 34.94 | 135.17 | 99 |
| **Kitchen** | 9.71 | 11.95 | 0 | 0 | 1.97 | 5.25 | 13.37 | 39.22 | 48.32 | 99 |
| **Corridors** | 7.08 | 11.02 | 0 | 0 | 0.31 | 3.18 | 9.94 | 20.31 | 74.52 | 99 |
| **Playroom** | 1.01 | 3.84 | 0 | 0 | 0 | 0 | 0 | 7.62 | 29.25 | 99 |
| **Bathroom** | 0.23 | 0.69 | 0 | 0 | 0 | 0 | 0 | 1.31 | 5.44 | 99 |
| **Balcony/Porch** | 0.18 | 0.94 | 0 | 0 | 0 | 0 | 0 | 0.44 | 8.18 | 99 |
| **Den** | 0.08 | 0.62 | 0 | 0 | 0 | 0 | 0 | 0 | 5.94 | 99 |
| **Laundry Room** | 0.01 | 0.07 | 0 | 0 | 0 | 0 | 0 | 0 | 0.66 | 99 |

*Values in visits/h. Observation time = 273.10 hours (Location Not-In-View excluded). Sorted by mean (descending).*

**Supplemental Table S13. Distribution of location time and visit frequency by Age Group.**

| **Group** | **Location** | **Med Dur** | **Q25 Dur** | **Q75 Dur** | **Mean Dur** | **SD Dur** | **Med Freq** | **Q25 Freq** | **Q75 Freq** | **Mean Freq** | **SD Freq** |
| --- | --- | --- | --- | --- | --- | --- | --- | --- | --- | --- | --- |
| ***12 to <24 months (n=25)*** | Balcony/Porch | 0 | 0 | 0 | 0.11 | 0.52 | 0 | 0 | 0 | 0.41 | 1.64 |
|  | Bathroom | 0 | 0 | 0 | 0.15 | 0.69 | 0 | 0 | 0 | 0.17 | 0.44 |
|  | Bedroom | 0.33 | 0 | 14.52 | 10.99 | 18.58 | 1.05 | 0 | 6.65 | 6.03 | 11.85 |
|  | Corridors | 0.56 | 0 | 1.34 | 1.4 | 2.58 | 3.3 | 0 | 8.9 | 5.27 | 5.48 |
|  | Den | 0 | 0 | 0 | 0.31 | 1.57 | 0 | 0 | 0 | 0.24 | 1.19 |
|  | Dining Room | 1.77 | 0 | 7.21 | 4.31 | 5.46 | 7.91 | 0 | 24.8 | 15.93 | 19.48 |
|  | Kitchen | 2.67 | 0.99 | 6.45 | 7.06 | 9.85 | 8.35 | 2.64 | 21.26 | 14.67 | 16.08 |
|  | Laundry Room | 0 | 0 | 0 | 0 | 0 | 0 | 0 | 0 | 0 | 0 |
|  | Living Room | 36.81 | 20.13 | 47.95 | 33.5 | 17.9 | 30.44 | 17.88 | 53.53 | 42.79 | 33.9 |
|  | Playroom | 0 | 0 | 0 | 2.16 | 7.92 | 0 | 0 | 0 | 1.01 | 3.51 |
| ***24 to <36 months (n=24)*** | Balcony/Porch | 0 | 0 | 0 | 0.33 | 1.53 | 0 | 0 | 0.29 | 0.28 | 0.9 |
|  | Bathroom | 0 | 0 | 0 | 0.03 | 0.09 | 0 | 0 | 0 | 0.18 | 0.49 |
|  | Bedroom | 0.37 | 0 | 8.55 | 8.04 | 14.58 | 2.17 | 0 | 5.3 | 3.25 | 3.78 |
|  | Corridors | 0.44 | 0.1 | 1.2 | 1.47 | 2.38 | 4.34 | 1.16 | 9.68 | 10.1 | 17.54 |
|  | Den | 0 | 0 | 0 | 0 | 0 | 0 | 0 | 0 | 0.01 | 0.07 |
|  | Dining Room | 3.49 | 0.33 | 7.24 | 6.45 | 9.83 | 10.15 | 1.81 | 17.78 | 14.83 | 19.31 |
|  | Kitchen | 2.64 | 0.73 | 5.46 | 3.93 | 4.33 | 6.83 | 2.68 | 14.64 | 10.22 | 10.31 |
|  | Laundry Room | 0 | 0 | 0 | 0 | 0 | 0 | 0 | 0 | 0.03 | 0.13 |
|  | Living Room | 42.13 | 22.29 | 50 | 37.19 | 16.52 | 30.4 | 22.49 | 40.57 | 36.34 | 25.52 |
|  | Playroom | 0 | 0 | 0 | 2.56 | 9.07 | 0 | 0 | 0 | 1.46 | 5.99 |
| ***36 to <72 months (n=33)*** | Balcony/Porch | 0 | 0 | 0 | 0 | 0.01 | 0 | 0 | 0 | 0.01 | 0.06 |
|  | Bathroom | 0 | 0 | 0.02 | 0.09 | 0.3 | 0 | 0 | 0.3 | 0.25 | 0.52 |
|  | Bedroom | 5.5 | 0.4 | 30.02 | 15.96 | 19.08 | 4.71 | 1.09 | 21.74 | 16.44 | 29.29 |
|  | Corridors | 0.48 | 0.16 | 0.89 | 1.05 | 1.79 | 8.33 | 1.59 | 13.82 | 9.54 | 9.54 |
|  | Den | 0 | 0 | 0 | 0.15 | 0.84 | 0 | 0 | 0 | 0.06 | 0.27 |
|  | Dining Room | 4.16 | 0.02 | 9.15 | 8.06 | 10.28 | 9.76 | 0.34 | 29.52 | 17.22 | 20.25 |
|  | Kitchen | 2.22 | 0.9 | 3.58 | 3 | 3.86 | 6.15 | 2.94 | 10.55 | 9.41 | 10.76 |
|  | Laundry Room | 0 | 0 | 0 | 0 | 0 | 0 | 0 | 0 | 0 | 0 |
|  | Living Room | 33 | 18.02 | 39.84 | 30.14 | 16.82 | 22.38 | 10.89 | 48.8 | 32.84 | 31.64 |
|  | Playroom | 0 | 0 | 0 | 1.54 | 5.42 | 0 | 0 | 0 | 0.89 | 2.69 |
| ***6 to <12 months (n=17)*** | Balcony/Porch | 0 | 0 | 0 | 0 | 0 | 0 | 0 | 0 | 0.02 | 0.07 |
|  | Bathroom | 0 | 0 | 0 | 0.55 | 1.67 | 0 | 0 | 0 | 0.37 | 1.31 |
|  | Bedroom | 28.26 | 0.56 | 43.94 | 25.32 | 21.39 | 5.47 | 1.07 | 14.06 | 12.49 | 24.5 |
|  | Corridors | 0.01 | 0 | 0.07 | 0.26 | 0.89 | 0.34 | 0 | 1.36 | 0.72 | 0.88 |
|  | Den | 0 | 0 | 0 | 0 | 0 | 0 | 0 | 0 | 0 | 0 |
|  | Dining Room | 0 | 0 | 4.48 | 4.08 | 7.27 | 0 | 0 | 3.77 | 11.06 | 21.48 |
|  | Kitchen | 0.46 | 0.06 | 2.6 | 2.55 | 4.39 | 1.6 | 0.33 | 3.87 | 2.29 | 2.41 |
|  | Laundry Room | 0 | 0 | 0 | 0 | 0 | 0 | 0 | 0 | 0 | 0 |
|  | Living Room | 25.82 | 6.87 | 46.84 | 26.88 | 22.77 | 21.3 | 2.03 | 43 | 26.63 | 24.63 |
|  | Playroom | 0 | 0 | 0 | 0.36 | 1.48 | 0 | 0 | 0 | 0.6 | 2.37 |

*Duration in min/h; Frequency in visits/h. Observation time = 273.10 hours (Location Not-In-View excluded).*

**Supplemental Table S14. Distribution of location time and visit frequency by Region.**

| **Group** | **Location** | **Med Dur** | **Q25 Dur** | **Q75 Dur** | **Mean Dur** | **SD Dur** | **Med Freq** | **Q25 Freq** | **Q75 Freq** | **Mean Freq** | **SD Freq** |
| --- | --- | --- | --- | --- | --- | --- | --- | --- | --- | --- | --- |
| ***Arizona (n=33)*** | Balcony/Porch | 0 | 0 | 0 | 0.01 | 0.03 | 0 | 0 | 0 | 0.06 | 0.16 |
|  | Bathroom | 0 | 0 | 0 | 0.06 | 0.25 | 0 | 0 | 0 | 0.11 | 0.31 |
|  | Bedroom | 1.93 | 0 | 14.52 | 10.18 | 14.41 | 2.2 | 0 | 6.72 | 4.76 | 6.4 |
|  | Corridors | 0.72 | 0.28 | 1.42 | 1.65 | 2.63 | 5.62 | 1.58 | 10.16 | 7.92 | 9.53 |
|  | Den | 0 | 0 | 0 | 0.24 | 1.37 | 0 | 0 | 0 | 0.19 | 1.03 |
|  | Dining Room | 6.95 | 0.98 | 12.19 | 8.2 | 8.36 | 13.36 | 3.77 | 28.97 | 19.09 | 19.5 |
|  | Kitchen | 3.06 | 1.8 | 6.52 | 5.44 | 6.21 | 7.04 | 3.4 | 17.13 | 12.19 | 12.7 |
|  | Laundry Room | 0 | 0 | 0 | 0 | 0 | 0 | 0 | 0 | 0.02 | 0.11 |
|  | Living Room | 33.01 | 23.03 | 43.89 | 32.36 | 13.65 | 27.83 | 14.55 | 39.47 | 29.4 | 19.87 |
|  | Playroom | 0 | 0 | 0 | 1.87 | 5.02 | 0 | 0 | 0 | 1.13 | 3.11 |
| ***Florida (n=33)*** | Balcony/Porch | 0 | 0 | 0 | 0.31 | 1.37 | 0 | 0 | 0 | 0.4 | 1.59 |
|  | Bathroom | 0 | 0 | 0.02 | 0.43 | 1.33 | 0 | 0 | 0.34 | 0.4 | 1.04 |
|  | Bedroom | 6.99 | 0.4 | 30.02 | 16.01 | 17.68 | 4.32 | 1.78 | 11.76 | 9.84 | 18.62 |
|  | Corridors | 0.12 | 0 | 0.76 | 0.7 | 1.64 | 1.82 | 0 | 8.33 | 6.64 | 9.95 |
|  | Den | 0 | 0 | 0 | 0.01 | 0.04 | 0 | 0 | 0 | 0.02 | 0.11 |
|  | Dining Room | 1.67 | 0 | 7.98 | 6.58 | 9.88 | 8.52 | 0 | 20.62 | 14.95 | 19.58 |
|  | Kitchen | 2.22 | 0.75 | 4.24 | 3.75 | 4.8 | 5.25 | 2.27 | 10.38 | 9.71 | 13.28 |
|  | Laundry Room | 0 | 0 | 0 | 0 | 0 | 0 | 0 | 0 | 0 | 0 |
|  | Living Room | 31.88 | 17.68 | 40.55 | 30.08 | 17.34 | 25.99 | 10.89 | 52.99 | 34.09 | 29.38 |
|  | Playroom | 0 | 0 | 0 | 2.13 | 8.54 | 0 | 0 | 0 | 1.15 | 5.17 |
| ***North Carolina (n=33)*** | Balcony/Porch | 0 | 0 | 0 | 0.01 | 0.04 | 0 | 0 | 0 | 0.07 | 0.22 |
|  | Bathroom | 0 | 0 | 0 | 0.02 | 0.08 | 0 | 0 | 0 | 0.19 | 0.51 |
|  | Bedroom | 0.56 | 0 | 43.68 | 16.99 | 23.7 | 1.07 | 0 | 14.4 | 15.21 | 30.37 |
|  | Corridors | 0.18 | 0 | 0.68 | 0.97 | 1.77 | 2.19 | 0 | 8.75 | 6.69 | 13.46 |
|  | Den | 0 | 0 | 0 | 0.15 | 0.84 | 0 | 0 | 0 | 0.04 | 0.25 |
|  | Dining Room | 0.05 | 0 | 2.97 | 3.34 | 7.23 | 1.38 | 0 | 11.8 | 11.74 | 20.39 |
|  | Kitchen | 0.93 | 0.08 | 3.07 | 3.34 | 7.47 | 3.37 | 0.67 | 12.87 | 7.23 | 9.34 |
|  | Laundry Room | 0 | 0 | 0 | 0 | 0 | 0 | 0 | 0 | 0 | 0 |
|  | Living Room | 41.24 | 12.04 | 54.75 | 33.98 | 22.88 | 26.95 | 16.85 | 68.24 | 41.9 | 37.21 |
|  | Playroom | 0 | 0 | 0 | 1.21 | 6.3 | 0 | 0 | 0 | 0.74 | 2.96 |

*Duration in min/h; Frequency in visits/h. Observation time = 273.10 hours.*

**Supplemental Table S15. Distribution of location time and visit frequency by Race.**

| **Group** | **Location** | **Med Dur** | **Q25 Dur** | **Q75 Dur** | **Mean Dur** | **SD Dur** | **Med Freq** | **Q25 Freq** | **Q75 Freq** | **Mean Freq** | **SD Freq** |
| --- | --- | --- | --- | --- | --- | --- | --- | --- | --- | --- | --- |
| ***Asian (n=6)*** | Balcony/Porch | 0 | 0 | 0 | 0.01 | 0.02 | 0 | 0 | 0 | 0.06 | 0.14 |
|  | Bathroom | 0 | 0 | 0.07 | 0.59 | 1.4 | 0 | 0 | 0.43 | 0.21 | 0.34 |
|  | Bedroom | 14.24 | 1.81 | 29.16 | 15.55 | 15.47 | 2.82 | 2.03 | 5.34 | 7.59 | 11.16 |
|  | Corridors | 0.46 | 0.01 | 0.89 | 0.5 | 0.55 | 4.48 | 0.16 | 11.55 | 7.45 | 9.27 |
|  | Den | 0 | 0 | 0 | 0.04 | 0.1 | 0 | 0 | 0 | 0.1 | 0.25 |
|  | Dining Room | 6.07 | 1.71 | 8.01 | 6.43 | 6.41 | 22.01 | 6.91 | 50.8 | 32.48 | 34.63 |
|  | Kitchen | 2.3 | 1.23 | 2.6 | 1.89 | 1.02 | 4.5 | 3.46 | 8.15 | 6.26 | 4.43 |
|  | Laundry Room | 0 | 0 | 0 | 0 | 0 | 0 | 0 | 0 | 0 | 0 |
|  | Living Room | 35.22 | 20.82 | 49.01 | 34.97 | 15.67 | 37.87 | 26.23 | 50.58 | 35.45 | 18.53 |
|  | Playroom | 0 | 0 | 0 | 0.02 | 0.04 | 0 | 0 | 0 | 0.05 | 0.13 |
| ***Black or African American (n=25)*** | Balcony/Porch | 0 | 0 | 0 | 0 | 0 | 0 | 0 | 0 | 0.02 | 0.09 |
|  | Bathroom | 0 | 0 | 0 | 0.12 | 0.57 | 0 | 0 | 0 | 0.1 | 0.26 |
|  | Bedroom | 1.99 | 0 | 43.94 | 19.94 | 24.3 | 5.35 | 0 | 14.4 | 20.73 | 37.37 |
|  | Corridors | 0.22 | 0 | 0.61 | 0.91 | 1.93 | 2.19 | 0 | 12.12 | 7.13 | 11.48 |
|  | Den | 0 | 0 | 0 | 0.19 | 0.97 | 0 | 0 | 0 | 0.06 | 0.29 |
|  | Dining Room | 0.02 | 0 | 4.8 | 4.74 | 9.48 | 0.34 | 0 | 17.68 | 11.57 | 19.24 |
|  | Kitchen | 0.9 | 0.09 | 1.8 | 2.67 | 7.5 | 3.28 | 0.69 | 6.88 | 6.37 | 8.06 |
|  | Laundry Room | 0 | 0 | 0 | 0 | 0 | 0 | 0 | 0 | 0 | 0 |
|  | Living Room | 24.67 | 4.43 | 49.15 | 28.63 | 23.1 | 30.12 | 3.68 | 56.79 | 38.83 | 38.57 |
|  | Playroom | 0 | 0 | 0 | 2.78 | 9.77 | 0 | 0 | 0 | 1.42 | 5.93 |
| ***Mixed (n=15)*** | Balcony/Porch | 0 | 0 | 0 | 0.02 | 0.06 | 0 | 0 | 0 | 0.06 | 0.17 |
|  | Bathroom | 0 | 0 | 0 | 0.03 | 0.1 | 0 | 0 | 0 | 0.23 | 0.58 |
|  | Bedroom | 0.4 | 0 | 4.68 | 6.61 | 12.93 | 0.3 | 0 | 2.76 | 3.19 | 6.06 |
|  | Corridors | 0.39 | 0 | 2.95 | 1.72 | 2.36 | 2.93 | 0.15 | 12.29 | 10.34 | 18.99 |
|  | Den | 0 | 0 | 0 | 0 | 0 | 0 | 0 | 0 | 0 | 0 |
|  | Dining Room | 1.67 | 0 | 7.62 | 4.3 | 5.7 | 3.34 | 0 | 12.19 | 10.14 | 13.38 |
|  | Kitchen | 3.75 | 1.81 | 6.2 | 5.2 | 5.53 | 9.67 | 2.92 | 24.28 | 14.8 | 14.79 |
|  | Laundry Room | 0 | 0 | 0 | 0 | 0 | 0 | 0 | 0 | 0 | 0 |
|  | Living Room | 43.03 | 29.41 | 53.83 | 39.46 | 17.15 | 25.87 | 18.06 | 42.81 | 33.76 | 26.43 |
|  | Playroom | 0 | 0 | 0 | 2.67 | 9.31 | 0 | 0 | 0 | 1.63 | 4.3 |
| ***Other (n=3)*** | Balcony/Porch | 0 | 0 | 0 | 0 | 0 | 0 | 0 | 0 | 0 | 0 |
|  | Bathroom | 0 | 0 | 0.01 | 0.01 | 0.01 | 0 | 0 | 0.88 | 0.59 | 1.02 |
|  | Bedroom | 4.66 | 2.5 | 9.6 | 6.52 | 7.28 | 7.12 | 5.91 | 9.72 | 8.05 | 3.89 |
|  | Corridors | 0.01 | 0.01 | 0.22 | 0.15 | 0.25 | 0.34 | 0.17 | 3.11 | 2.07 | 3.3 |
|  | Den | 0 | 0 | 0 | 0 | 0 | 0 | 0 | 0 | 0 | 0 |
|  | Dining Room | 20.3 | 18.87 | 21.41 | 20.08 | 2.55 | 25.77 | 22.45 | 49.6 | 39.44 | 29.62 |
|  | Kitchen | 11.64 | 6.46 | 13.84 | 9.65 | 7.58 | 8.48 | 5.71 | 28.4 | 19.91 | 24.75 |
|  | Laundry Room | 0 | 0 | 0 | 0 | 0 | 0 | 0 | 0 | 0 | 0 |
|  | Living Room | 26.27 | 16.57 | 31.95 | 23.59 | 15.55 | 23.54 | 12.78 | 38.53 | 26.37 | 25.86 |
|  | Playroom | 0 | 0 | 0.01 | 0.01 | 0.01 | 0 | 0 | 0.17 | 0.11 | 0.2 |
| ***White (n=50)*** | Balcony/Porch | 0 | 0 | 0 | 0.21 | 1.12 | 0 | 0 | 0 | 0.31 | 1.31 |
|  | Bathroom | 0 | 0 | 0 | 0.2 | 0.94 | 0 | 0 | 0 | 0.28 | 0.87 |
|  | Bedroom | 3.71 | 0.1 | 28.32 | 14.28 | 17.97 | 3.67 | 0.35 | 8.94 | 6.96 | 10.05 |
|  | Corridors | 0.42 | 0.11 | 1.19 | 1.15 | 2.23 | 3.54 | 1.38 | 8.86 | 6.34 | 7.79 |
|  | Den | 0 | 0 | 0 | 0.16 | 1.11 | 0 | 0 | 0 | 0.13 | 0.84 |
|  | Dining Room | 2.56 | 0.06 | 8.23 | 6.32 | 8.95 | 8.22 | 0.94 | 23.95 | 15.12 | 17.63 |
|  | Kitchen | 2.44 | 0.76 | 6.11 | 4.57 | 5.97 | 5.32 | 2.02 | 13.78 | 9.66 | 11.96 |
|  | Laundry Room | 0 | 0 | 0 | 0 | 0 | 0 | 0 | 0 | 0.01 | 0.09 |
|  | Living Room | 34.62 | 20.09 | 44.09 | 31.87 | 16.01 | 28.15 | 12.82 | 44.44 | 34.18 | 27.86 |
|  | Playroom | 0 | 0 | 0 | 1.25 | 4.15 | 0 | 0 | 0 | 0.78 | 2.58 |

*Duration in min/h; Frequency in visits/h. Observation time = 273.10 hours.*

**Supplemental Table S16. Distribution of location time and visit frequency by Ethnicity.**

| **Group** | **Location** | **Med Dur** | **Q25 Dur** | **Q75 Dur** | **Mean Dur** | **SD Dur** | **Med Freq** | **Q25 Freq** | **Q75 Freq** | **Mean Freq** | **SD Freq** |
| --- | --- | --- | --- | --- | --- | --- | --- | --- | --- | --- | --- |
| ***Hispanic (n=39)*** | Balcony/Porch | 0 | 0 | 0 | 0.26 | 1.26 | 0 | 0 | 0 | 0.35 | 1.47 |
|  | Bathroom | 0 | 0 | 0 | 0.23 | 1.05 | 0 | 0 | 0 | 0.32 | 0.96 |
|  | Bedroom | 4.66 | 0.05 | 30.96 | 17.35 | 19.65 | 3.93 | 0.27 | 12.37 | 8.23 | 11.2 |
|  | Corridors | 0.23 | 0.02 | 0.5 | 0.69 | 1.6 | 1.89 | 0.64 | 5.75 | 5.35 | 8.43 |
|  | Den | 0 | 0 | 0 | 0.2 | 1.26 | 0 | 0 | 0 | 0.16 | 0.95 |
|  | Dining Room | 4.01 | 0.31 | 16.03 | 8.92 | 10.87 | 13.04 | 1.6 | 27.8 | 19.28 | 21.5 |
|  | Kitchen | 1.51 | 0.39 | 3.6 | 3.53 | 4.93 | 3.94 | 1.43 | 9.43 | 8.54 | 11.6 |
|  | Laundry Room | 0 | 0 | 0 | 0 | 0 | 0 | 0 | 0 | 0.02 | 0.11 |
|  | Living Room | 28.19 | 12.38 | 38.43 | 27.21 | 17.16 | 20.18 | 10.84 | 39.62 | 28.91 | 26.09 |
|  | Playroom | 0 | 0 | 0 | 1.6 | 5.65 | 0 | 0 | 0 | 0.76 | 2.21 |
| ***Non-Hispanic (n=60)*** | Balcony/Porch | 0 | 0 | 0 | 0.01 | 0.04 | 0 | 0 | 0 | 0.07 | 0.19 |
|  | Bathroom | 0 | 0 | 0 | 0.14 | 0.58 | 0 | 0 | 0 | 0.18 | 0.44 |
|  | Bedroom | 1.96 | 0 | 18.43 | 12.47 | 18.54 | 2.3 | 0 | 7.05 | 11.05 | 25.63 |
|  | Corridors | 0.55 | 0 | 1.36 | 1.37 | 2.31 | 4.45 | 0 | 12.05 | 8.21 | 12.36 |
|  | Den | 0 | 0 | 0 | 0.08 | 0.62 | 0 | 0 | 0 | 0.03 | 0.2 |
|  | Dining Room | 0.94 | 0 | 7.01 | 4.17 | 6.4 | 3.93 | 0 | 19.12 | 12.64 | 18.42 |
|  | Kitchen | 2.36 | 0.89 | 5.6 | 4.59 | 7 | 5.76 | 2.15 | 15.05 | 10.47 | 12.21 |
|  | Laundry Room | 0 | 0 | 0 | 0 | 0 | 0 | 0 | 0 | 0 | 0 |
|  | Living Room | 39.69 | 19.61 | 49.57 | 35.34 | 18.33 | 30.68 | 17.62 | 49.39 | 39.18 | 31.58 |
|  | Playroom | 0 | 0 | 0 | 1.83 | 7.38 | 0 | 0 | 0 | 1.17 | 4.62 |

*Duration in min/h; Frequency in visits/h. Observation time = 273.10 hours.*

**Supplemental Table S17. Distribution of location time and visit frequency by Sex.**

| **Group** | **Location** | **Med Dur** | **Q25 Dur** | **Q75 Dur** | **Mean Dur** | **SD Dur** | **Med Freq** | **Q25 Freq** | **Q75 Freq** | **Mean Freq** | **SD Freq** |
| --- | --- | --- | --- | --- | --- | --- | --- | --- | --- | --- | --- |
| ***F (n=47)*** | Balcony/Porch | 0 | 0 | 0 | 0.17 | 1.1 | 0 | 0 | 0 | 0.13 | 0.65 |
|  | Bathroom | 0 | 0 | 0 | 0.3 | 1.13 | 0 | 0 | 0 | 0.3 | 0.9 |
|  | Bedroom | 1.93 | 0 | 19.41 | 13.07 | 18.92 | 2.14 | 0 | 9.96 | 7.44 | 15.94 |
|  | Corridors | 0.36 | 0.01 | 1.25 | 1.09 | 1.86 | 2.93 | 0.31 | 8.82 | 7.32 | 13.38 |
|  | Den | 0 | 0 | 0 | 0 | 0 | 0 | 0 | 0 | 0 | 0 |
|  | Dining Room | 1.39 | 0 | 7.54 | 5.47 | 8.78 | 4.28 | 0 | 19.87 | 13.13 | 18.63 |
|  | Kitchen | 2.6 | 1.2 | 6.27 | 4.35 | 4.94 | 5.28 | 1.97 | 17 | 12.56 | 15.19 |
|  | Laundry Room | 0 | 0 | 0 | 0 | 0 | 0 | 0 | 0 | 0 | 0 |
|  | Living Room | 39.23 | 18 | 49.11 | 34.06 | 18.06 | 28.47 | 13.79 | 45.25 | 34.35 | 27.11 |
|  | Playroom | 0 | 0 | 0 | 1.48 | 6.58 | 0 | 0 | 0 | 0.97 | 4.66 |
| ***M (n=51)*** | Balcony/Porch | 0 | 0 | 0 | 0.06 | 0.36 | 0 | 0 | 0 | 0.23 | 1.15 |
|  | Bathroom | 0 | 0 | 0 | 0.05 | 0.21 | 0 | 0 | 0 | 0.17 | 0.43 |
|  | Bedroom | 3.62 | 0.19 | 30.36 | 15.89 | 19.31 | 3.93 | 0.29 | 10.02 | 12.43 | 25.03 |
|  | Corridors | 0.33 | 0.01 | 0.89 | 0.89 | 1.64 | 3.18 | 0.3 | 11.12 | 6.62 | 8.37 |
|  | Den | 0 | 0 | 0 | 0.25 | 1.28 | 0 | 0 | 0 | 0.16 | 0.85 |
|  | Dining Room | 2.97 | 0.03 | 10.1 | 6.68 | 8.74 | 10.85 | 0.34 | 27.8 | 17.52 | 20.96 |
|  | Kitchen | 1.27 | 0.35 | 3.89 | 4.1 | 7.35 | 5.25 | 2.36 | 9.39 | 7.28 | 7.23 |
|  | Laundry Room | 0 | 0 | 0 | 0 | 0 | 0 | 0 | 0 | 0.01 | 0.09 |
|  | Living Room | 30.74 | 16.83 | 43.21 | 30.06 | 18.41 | 26.14 | 11.88 | 48.98 | 35.66 | 32.63 |
|  | Playroom | 0 | 0 | 0 | 2.01 | 6.97 | 0 | 0 | 0 | 1.06 | 3 |
| ***O (n=1)*** | Balcony/Porch | 0 | 0 | 0 | 0 | NA | 0 | 0 | 0 | 0 | NA |
|  | Bathroom | 0 | 0 | 0 | 0 | NA | 0 | 0 | 0 | 0 | NA |
|  | Bedroom | 0 | 0 | 0 | 0 | NA | 0 | 0 | 0 | 0 | NA |
|  | Corridors | 12.42 | 12.42 | 12.42 | 12.42 | NA | 19.25 | 19.25 | 19.25 | 19.25 | NA |
|  | Den | 0 | 0 | 0 | 0 | NA | 0 | 0 | 0 | 0 | NA |
|  | Dining Room | 0 | 0 | 0 | 0 | NA | 0 | 0 | 0 | 0 | NA |
|  | Kitchen | 0 | 0 | 0 | 0 | NA | 0 | 0 | 0 | 0 | NA |
|  | Laundry Room | 0 | 0 | 0 | 0 | NA | 0 | 0 | 0 | 0 | NA |
|  | Living Room | 47.58 | 47.58 | 47.58 | 47.58 | NA | 44.92 | 44.92 | 44.92 | 44.92 | NA |
|  | Playroom | 0 | 0 | 0 | 0 | NA | 0 | 0 | 0 | 0 | NA |

*Duration in min/h; Frequency in visits/h. Observation time = 273.10 hours.*

**Supplemental Table S18. Location time and visit frequency statistical tests by all demographics.**

| **Location** | **Demographic** | **Metric** | **Test** | **Statistic** | **df** | **p_value** | **Sig** |
| --- | --- | --- | --- | --- | --- | --- | --- |
| **Bedroom** | Age | Duration | Kruskal-Wallis | 9.326 | 3 | 0.0253 | * |
|  | Age | Frequency | Kruskal-Wallis | 8.776 | 3 | 0.0324 | * |
|  | Region | Duration | Kruskal-Wallis | 3.06 | 2 | 0.2166 | ns |
|  | Region | Frequency | Kruskal-Wallis | 2.588 | 2 | 0.2742 | ns |
|  | Sex | Duration | Kruskal-Wallis | 2.504 | 2 | 0.286 | ns |
|  | Sex | Frequency | Kruskal-Wallis | 2.456 | 2 | 0.2929 | ns |
|  | Race | Duration | Kruskal-Wallis | 3.821 | 4 | 0.4308 | ns |
|  | Race | Frequency | Kruskal-Wallis | 5.087 | 4 | 0.2785 | ns |
|  | Ethnicity | Duration | Mann-Whitney U | 1300 | — | 0.3476 | ns |
|  | Ethnicity | Frequency | Mann-Whitney U | 1281 | — | 0.4235 | ns |
| **Corridors** | Age | Duration | Kruskal-Wallis | 13.971 | 3 | 0.0029 | ** |
|  | Age | Frequency | Kruskal-Wallis | 18.73 | 3 | < 0.001 | *** |
|  | Region | Duration | Kruskal-Wallis | 10.562 | 2 | 0.0051 | ** |
|  | Region | Frequency | Kruskal-Wallis | 5.202 | 2 | 0.0742 | ns |
|  | Sex | Duration | Kruskal-Wallis | 3.248 | 2 | 0.1971 | ns |
|  | Sex | Frequency | Kruskal-Wallis | 2.293 | 2 | 0.3178 | ns |
|  | Race | Duration | Kruskal-Wallis | 4.446 | 4 | 0.349 | ns |
|  | Race | Frequency | Kruskal-Wallis | 1.717 | 4 | 0.7876 | ns |
|  | Ethnicity | Duration | Mann-Whitney U | 963 | — | 0.1367 | ns |
|  | Ethnicity | Frequency | Mann-Whitney U | 1040 | — | 0.3507 | ns |
| **Dining Room** | Age | Duration | Kruskal-Wallis | 5.517 | 3 | 0.1377 | ns |
|  | Age | Frequency | Kruskal-Wallis | 4.879 | 3 | 0.1809 | ns |
|  | Region | Duration | Kruskal-Wallis | 10.472 | 2 | 0.0053 | ** |
|  | Region | Frequency | Kruskal-Wallis | 5.842 | 2 | 0.0539 | ns |
|  | Sex | Duration | Kruskal-Wallis | 2.756 | 2 | 0.252 | ns |
|  | Sex | Frequency | Kruskal-Wallis | 3.115 | 2 | 0.2107 | ns |
|  | Race | Duration | Kruskal-Wallis | 10.897 | 4 | 0.0277 | * |
|  | Race | Frequency | Kruskal-Wallis | 9.09 | 4 | 0.0589 | ns |
|  | Ethnicity | Duration | Mann-Whitney U | 1512.5 | — | 0.013 | * |
|  | Ethnicity | Frequency | Mann-Whitney U | 1441.5 | — | 0.049 | * |
| **Kitchen** | Age | Duration | Kruskal-Wallis | 6.259 | 3 | 0.0997 | ns |
|  | Age | Frequency | Kruskal-Wallis | 13.633 | 3 | 0.0034 | ** |
|  | Region | Duration | Kruskal-Wallis | 9.935 | 2 | 0.007 | ** |
|  | Region | Frequency | Kruskal-Wallis | 5.434 | 2 | 0.0661 | ns |
|  | Sex | Duration | Kruskal-Wallis | 4.817 | 2 | 0.0899 | ns |
|  | Sex | Frequency | Kruskal-Wallis | 3.006 | 2 | 0.2224 | ns |
|  | Race | Duration | Kruskal-Wallis | 12.797 | 4 | 0.0123 | * |
|  | Race | Frequency | Kruskal-Wallis | 5.509 | 4 | 0.2389 | ns |
|  | Ethnicity | Duration | Mann-Whitney U | 1068 | — | 0.467 | ns |
|  | Ethnicity | Frequency | Mann-Whitney U | 1040 | — | 0.3534 | ns |
| **Living Room** | Age | Duration | Kruskal-Wallis | 3.537 | 3 | 0.3159 | ns |
|  | Age | Frequency | Kruskal-Wallis | 3.886 | 3 | 0.274 | ns |
|  | Region | Duration | Kruskal-Wallis | 1.348 | 2 | 0.5096 | ns |
|  | Region | Frequency | Kruskal-Wallis | 0.738 | 2 | 0.6913 | ns |
|  | Sex | Duration | Kruskal-Wallis | 2.034 | 2 | 0.3617 | ns |
|  | Sex | Frequency | Kruskal-Wallis | 0.702 | 2 | 0.704 | ns |
|  | Race | Duration | Kruskal-Wallis | 4.168 | 4 | 0.3838 | ns |
|  | Race | Frequency | Kruskal-Wallis | 0.511 | 4 | 0.9725 | ns |
|  | Ethnicity | Duration | Mann-Whitney U | 844.5 | — | 0.0199 | * |
|  | Ethnicity | Frequency | Mann-Whitney U | 911.5 | — | 0.0647 | ns |
| **Playroom** | Age | Duration | Kruskal-Wallis | 0.582 | 3 | 0.9005 | ns |
|  | Age | Frequency | Kruskal-Wallis | 0.535 | 3 | 0.911 | ns |
|  | Region | Duration | Kruskal-Wallis | 1.616 | 2 | 0.4458 | ns |
|  | Region | Frequency | Kruskal-Wallis | 1.49 | 2 | 0.4748 | ns |
|  | Sex | Duration | Kruskal-Wallis | 1.222 | 2 | 0.5428 | ns |
|  | Sex | Frequency | Kruskal-Wallis | 1.248 | 2 | 0.5358 | ns |
|  | Race | Duration | Kruskal-Wallis | 1.199 | 4 | 0.8783 | ns |
|  | Race | Frequency | Kruskal-Wallis | 1.334 | 4 | 0.8556 | ns |
|  | Ethnicity | Duration | Mann-Whitney U | 1228 | — | 0.4676 | ns |
|  | Ethnicity | Frequency | Mann-Whitney U | 1225 | — | 0.4912 | ns |

*Duration = time spent in location (min/h); Frequency = visit frequency (visits/h).*

*Kruskal-Wallis for ≥3 groups; Mann-Whitney U for 2 groups. * p < 0.05, ** p < 0.01, *** p < 0.001.*

**Supplemental Table S19. Location post-hoc pairwise comparisons (Wilcoxon rank-sum with Bonferroni correction).**

| **Location** | **Demographic** | **Metric** | **Group1** | **Group2** | **p_value** | **Sig** |
| --- | --- | --- | --- | --- | --- | --- |
| **Bedroom** | Age | Duration | 12 to <24 months | 24 to <36 months | 1 | ns |
|  | Age | Duration | 12 to <24 months | 36 to <72 months | 0.3115 | ns |
|  | Age | Duration | 12 to <24 months | 6 to <12 months | 0.2154 | ns |
|  | Age | Duration | 24 to <36 months | 36 to <72 months | 0.2228 | ns |
|  | Age | Duration | 24 to <36 months | 6 to <12 months | 0.1384 | ns |
|  | Age | Duration | 36 to <72 months | 6 to <12 months | 1 | ns |
|  | Age | Frequency | 12 to <24 months | 24 to <36 months | 1 | ns |
|  | Age | Frequency | 12 to <24 months | 36 to <72 months | 0.1354 | ns |
|  | Age | Frequency | 12 to <24 months | 6 to <12 months | 0.7032 | ns |
|  | Age | Frequency | 24 to <36 months | 36 to <72 months | 0.1478 | ns |
|  | Age | Frequency | 24 to <36 months | 6 to <12 months | 0.2885 | ns |
|  | Age | Frequency | 36 to <72 months | 6 to <12 months | 1 | ns |
| **Corridors** | Age | Duration | 12 to <24 months | 24 to <36 months | 1 | ns |
|  | Age | Duration | 12 to <24 months | 36 to <72 months | 1 | ns |
|  | Age | Duration | 12 to <24 months | 6 to <12 months | 0.0353 | * |
|  | Age | Duration | 24 to <36 months | 36 to <72 months | 1 | ns |
|  | Age | Duration | 24 to <36 months | 6 to <12 months | 0.0074 | * |
|  | Age | Duration | 36 to <72 months | 6 to <12 months | 0.0022 | * |
|  | Age | Frequency | 12 to <24 months | 24 to <36 months | 1 | ns |
|  | Age | Frequency | 12 to <24 months | 36 to <72 months | 0.4502 | ns |
|  | Age | Frequency | 12 to <24 months | 6 to <12 months | 0.0301 | * |
|  | Age | Frequency | 24 to <36 months | 36 to <72 months | 1 | ns |
|  | Age | Frequency | 24 to <36 months | 6 to <12 months | 0.0046 | * |
|  | Age | Frequency | 36 to <72 months | 6 to <12 months | < 0.001 | * |
|  | Region | Duration | Arizona | Florida | 0.0093 | * |
|  | Region | Duration | Arizona | North Carolina | 0.0257 | * |
|  | Region | Duration | Florida | North Carolina | 1 | ns |
| **Dining Room** | Region | Duration | Arizona | Florida | 0.3681 | ns |
|  | Region | Duration | Arizona | North Carolina | 0.0029 | * |
|  | Region | Duration | Florida | North Carolina | 0.3881 | ns |
|  | Race | Duration | Asian | Black or African American | 1 | ns |
|  | Race | Duration | Asian | Mixed | 1 | ns |
|  | Race | Duration | Asian | Other | 0.5281 | ns |
|  | Race | Duration | Asian | White | 1 | ns |
|  | Race | Duration | Black or African American | Mixed | 1 | ns |
|  | Race | Duration | Black or African American | Other | 0.1647 | ns |
|  | Race | Duration | Black or African American | White | 0.5759 | ns |
|  | Race | Duration | Mixed | Other | 0.1128 | ns |
|  | Race | Duration | Mixed | White | 1 | ns |
|  | Race | Duration | Other | White | 0.1568 | ns |
| **Kitchen** | Age | Frequency | 12 to <24 months | 24 to <36 months | 1 | ns |
|  | Age | Frequency | 12 to <24 months | 36 to <72 months | 1 | ns |
|  | Age | Frequency | 12 to <24 months | 6 to <12 months | 0.0262 | * |
|  | Age | Frequency | 24 to <36 months | 36 to <72 months | 1 | ns |
|  | Age | Frequency | 24 to <36 months | 6 to <12 months | 0.0053 | * |
|  | Age | Frequency | 36 to <72 months | 6 to <12 months | 0.0089 | * |
|  | Region | Duration | Arizona | Florida | 0.3954 | ns |
|  | Region | Duration | Arizona | North Carolina | 0.0069 | * |
|  | Region | Duration | Florida | North Carolina | 0.2193 | ns |
|  | Race | Duration | Asian | Black or African American | 1 | ns |
|  | Race | Duration | Asian | Mixed | 1 | ns |
|  | Race | Duration | Asian | Other | 1 | ns |
|  | Race | Duration | Asian | White | 1 | ns |
|  | Race | Duration | Black or African American | Mixed | 0.0467 | * |
|  | Race | Duration | Black or African American | Other | 0.528 | ns |
|  | Race | Duration | Black or African American | White | 0.0682 | ns |
|  | Race | Duration | Mixed | Other | 1 | ns |
|  | Race | Duration | Mixed | White | 1 | ns |
|  | Race | Duration | Other | White | 1 | ns |

*Only shown for significant Kruskal-Wallis results with ≥3 groups. * p < 0.05 after Bonferroni correction.*

**Supplemental Table S20. Complete surface × location mouthing overlap.**

| **Object/Surface** | **Location** | **Duration_sec** | **Duration_min** | **Frequency** | **N_Subjects** |
| --- | --- | --- | --- | --- | --- |
| **Pacifier** | Living Room | 21909 | 365.15 | 113 | 10 |
| **Other_Food** | Dining Room | 15183 | 253.05 | 757 | 44 |
|  | Living Room | 13636 | 227.27 | 672 | 55 |
| **Hands** | Living Room | 13331 | 222.18 | 881 | 73 |
| **Food-Container** | Living Room | 13188 | 219.8 | 654 | 51 |
| **Pacifier** | Bedroom | 11940 | 199 | 54 | 7 |
| **Food-Container** | Bedroom | 11361 | 189.35 | 142 | 19 |
| **Hands** | Bedroom | 9357 | 155.95 | 458 | 34 |
| **Hair/Body** | Bedroom | 7916 | 131.93 | 58 | 13 |
|  | Living Room | 7103 | 118.38 | 106 | 21 |
| **Other_Food** | Kitchen | 5586 | 93.1 | 214 | 33 |
| **Food-Container** | Dining Room | 4474 | 74.57 | 597 | 35 |
| **Other_Food** | Bedroom | 4190 | 69.83 | 170 | 12 |
| **Porous-Plastic-Toy** | Living Room | 4189 | 69.82 | 226 | 28 |
| **Sticky_Food** | Living Room | 2943 | 49.05 | 164 | 15 |
|  | Bedroom | 2679 | 44.65 | 58 | 6 |
| **Hands** | Dining Room | 2642 | 44.03 | 291 | 40 |
| **Bedding/Towels** | Bedroom | 2150 | 35.83 | 89 | 7 |
| **Food-Container** | Kitchen | 1765 | 29.42 | 136 | 27 |
| **Bedding/Towels** | Living Room | 1748 | 29.13 | 100 | 16 |
| **Pacifier** | Kitchen | 1572 | 26.2 | 26 | 2 |
| **Clothes** | Living Room | 1298 | 21.63 | 39 | 13 |
| **Beverage** | Living Room | 1193 | 19.88 | 127 | 17 |
| **Paper/Wrapper** | Living Room | 1177 | 19.62 | 94 | 17 |
| **Hands** | Kitchen | 759 | 12.65 | 105 | 38 |
| **Sticky_Food** | Dining Room | 747 | 12.45 | 51 | 9 |
| **Beverage** | Dining Room | 648 | 10.8 | 59 | 12 |
| **Sticky_Food** | Corridors | 604 | 10.07 | 31 | 5 |
| **Hands** | Playroom | 598 | 9.97 | 31 | 7 |
| **Beverage** | Kitchen | 556 | 9.27 | 71 | 8 |
| **Sticky_Food** | Kitchen | 504 | 8.4 | 37 | 13 |
| **Fabric_Toy** | Living Room | 458 | 7.63 | 29 | 10 |
| **Clothes** | Bedroom | 445 | 7.42 | 25 | 11 |
| **Food-Container** | Playroom | 321 | 5.35 | 20 | 4 |
| **Pacifier** | Dining Room | 297 | 4.95 | 20 | 5 |
| **Fabric_Wall/Furniture** | Living Room | 281 | 4.68 | 43 | 16 |
| **Other_Food** | Corridors | 262 | 4.37 | 27 | 11 |
| **Hard_Toy** | Living Room | 259 | 4.32 | 32 | 7 |
| **Porous-Plastic-Toy** | Dining Room | 255 | 4.25 | 21 | 8 |
| **Other_Food** | Playroom | 200 | 3.33 | 8 | 1 |
| **Plastic-Tool/Appliance** | Living Room | 199 | 3.32 | 21 | 5 |
| **Porous-Plastic-Toy** | Playroom | 198 | 3.3 | 22 | 5 |
| **Food-Container** | Corridors | 188 | 3.13 | 31 | 10 |
| **Porous-Plastic-Toy** | Bedroom | 160 | 2.67 | 37 | 5 |
| **Pacifier** | Corridors | 133 | 2.22 | 22 | 5 |
| **Plastic-Tool/Appliance** | Bedroom | 119 | 1.98 | 6 | 2 |
| **Porous-Plastic-Toy** | Kitchen | 112 | 1.87 | 20 | 8 |
| **Wood_Wall/Furniture** | Bedroom | 112 | 1.87 | 3 | 1 |
| **Electronics** | Living Room | 106 | 1.77 | 5 | 3 |
| **Clothes** | Dining Room | 100 | 1.67 | 11 | 5 |
| **Plastic-Tool/Appliance** | Dining Room | 88 | 1.47 | 14 | 4 |
| **Paper/Wrapper** | Dining Room | 76 | 1.27 | 26 | 11 |
| **Hands** | Corridors | 74 | 1.23 | 26 | 15 |
| **Electronics** | Bedroom | 65 | 1.08 | 8 | 4 |
| **Paper/Wrapper** | Kitchen | 58 | 0.97 | 5 | 4 |
| **Hands** | Den | 56 | 0.93 | 1 | 1 |
| **Clothes** | Kitchen | 55 | 0.92 | 10 | 5 |
| **Hair/Body** | Dining Room | 41 | 0.68 | 4 | 2 |
| **Fabric_Wall/Furniture** | Dining Room | 32 | 0.53 | 2 | 2 |
| **Metal_Wall/Furniture** | Dining Room | 31 | 0.52 | 7 | 2 |
| **Hard_Toy** | Playroom | 28 | 0.47 | 9 | 1 |
| **Porous-Plastic-Toy** | Corridors | 24 | 0.4 | 8 | 4 |
| **Hair/Body** | Kitchen | 23 | 0.38 | 4 | 3 |
| **Clothes** | Corridors | 22 | 0.37 | 6 | 1 |
| **Fabric_Toy** | Bedroom | 21 | 0.35 | 8 | 4 |
| **Hair/Body** | Balcony/Porch | 16 | 0.27 | 2 | 1 |
| **Plastic_Wall/Furniture** | Dining Room | 14 | 0.23 | 1 | 1 |
| **Beverage** | Corridors | 13 | 0.22 | 5 | 4 |
| **Carpet/Mat** | Living Room | 12 | 0.2 | 1 | 1 |
| **Fabric_Wall/Furniture** | Bedroom | 11 | 0.18 | 7 | 4 |
| **Hard_Toy** | Bedroom | 11 | 0.18 | 6 | 3 |
| **Bedding/Towels** | Dining Room | 10 | 0.17 | 1 | 1 |
| **Wood_Toy** | Bedroom | 10 | 0.17 | 1 | 1 |
| **Other_Food** | Balcony/Porch | 9 | 0.15 | 1 | 1 |
| **Animal** | Bedroom | 8 | 0.13 | 1 | 1 |
| **Bedding/Towels** | Corridors | 8 | 0.13 | 2 | 2 |
| **Metal_Wall/Furniture** | Kitchen | 7 | 0.12 | 2 | 1 |
| **Plastic_Wall/Furniture** | Living Room | 6 | 0.1 | 1 | 1 |
| **Footwear** | Living Room | 5 | 0.08 | 5 | 2 |
| **Pacifier** | Bathroom | 5 | 0.08 | 3 | 1 |
| **Water** | Dining Room | 5 | 0.08 | 3 | 3 |
| **Beverage** | Bedroom | 4 | 0.07 | 1 | 1 |
| **Bedding/Towels** | Kitchen | 3 | 0.05 | 1 | 1 |
| **Paper/Wrapper** | Corridors | 3 | 0.05 | 1 | 1 |
| **Water** | Kitchen | 3 | 0.05 | 3 | 2 |
| **Pacifier** | Balcony/Porch | 2 | 0.03 | 2 | 1 |
| **Water** | Bedroom | 2 | 0.03 | 2 | 1 |
|  | Living Room | 2 | 0.03 | 1 | 1 |
| **Animal** | Living Room | 1 | 0.02 | 1 | 1 |
| **Hair/Body** | Playroom | 1 | 0.02 | 1 | 1 |
| **Hands** | Balcony/Porch | 1 | 0.02 | 1 | 1 |
| **Other_Food** | Laundry Room | 1 | 0.02 | 1 | 1 |
| **Paper/Wrapper** | Bedroom | 1 | 0.02 | 1 | 1 |
| **Plastic-Tool/Appliance** | Kitchen | 1 | 0.02 | 1 | 1 |
| **Animal** | Dining Room | 0 | 0 | 1 | 1 |
| **Hard_Toy** | Dining Room | 0 | 0 | 1 | 1 |
| **Metal_Tool/Appliance** | Bedroom | 0 | 0 | 1 | 1 |
| **Nose** | Living Room | 0 | 0 | 2 | 2 |

*Duration and frequency calculated from active mouthing events. Sorted by duration (descending).*

**Supplemental Table S21. Statistical difference of surface mouthing BY location (Kruskal-Wallis).**

| **Object/Surface** | **Test** | **Statistic** | **df** | **p_value** | **Significance** |
| --- | --- | --- | --- | --- | --- |
| **Other_Food** | Kruskal-Wallis | 129.569 | 5 | < 0.001 | *** |
| **Pacifier** | Kruskal-Wallis | 6.777 | 4 | 0.1482 | ns |
| **Food-Container** | Kruskal-Wallis | 85.715 | 5 | < 0.001 | *** |
| **Hands** | Kruskal-Wallis | 135.919 | 5 | < 0.001 | *** |
| **Hair/Body** | Kruskal-Wallis | 41.193 | 4 | < 0.001 | *** |
| **Sticky_Food** | Kruskal-Wallis | 8.127 | 4 | 0.087 | ns |
| **Porous-Plastic-Toy** | Kruskal-Wallis | 50.54 | 5 | < 0.001 | *** |
| **Bedding/Towels** | Kruskal-Wallis | 32.418 | 4 | < 0.001 | *** |
| **Beverage** | Kruskal-Wallis | 21.61 | 4 | < 0.001 | *** |
| **Clothes** | Kruskal-Wallis | 13.96 | 4 | 0.0074 | ** |

*Tests whether mouthing duration for each surface differs across top 6 locations. * p < 0.05, ** p < 0.01, *** p < 0.001.*

**Supplemental Table S22. Statistical difference of location mouthing BY surface (Kruskal-Wallis).**

| **Location** | **Test** | **Statistic** | **df** | **p_value** | **Significance** |
| --- | --- | --- | --- | --- | --- |
| **Living Room** | Kruskal-Wallis | 211.249 | 9 | < 0.001 | *** |
| **Bedroom** | Kruskal-Wallis | 77.678 | 9 | < 0.001 | *** |
| **Dining Room** | Kruskal-Wallis | 191.391 | 9 | < 0.001 | *** |
| **Kitchen** | Kruskal-Wallis | 143.648 | 9 | < 0.001 | *** |
| **Corridors** | Kruskal-Wallis | 24.413 | 8 | 0.002 | ** |
| **Playroom** | Kruskal-Wallis | 7.785 | 4 | 0.0998 | ns |

*Tests whether mouthing duration for each location differs across top 10 surfaces. * p < 0.05, ** p < 0.01, *** p < 0.001.*
